# Rapid Assessment of Ecosystem Service Co-Benefits of Biodiversity Priority Areas in Madagascar

**DOI:** 10.1371/journal.pone.0168575

**Published:** 2016-12-22

**Authors:** Rachel A. Neugarten, Miroslav Honzák, Pierre Carret, Kellee Koenig, Luciano Andriamaro, Carlos Andres Cano, Hedley S. Grantham, David Hole, Daniel Juhn, Madeleine McKinnon, Andriambolantsoa Rasolohery, Marc Steininger, Timothy Max Wright, Will R. Turner

**Affiliations:** 1 Conservation International, Arlington, Virginia, United States of America; 2 Critical Ecosystem Partnership Fund, Arlington, Virginia, United States of America; 3 Conservation International, Antananarivo, Madagascar; 4 Wildlife Conservation Society, Byron Bay, Australia; 5 Vulcan, Inc. Seattle, Washington, United States of America; 6 Department of Geographical Sciences, University of Maryland, College Park, Maryland, United States of America; Centre for Cellular and Molecular Biology, INDIA

## Abstract

The importance of ecosystems for supporting human well-being is increasingly recognized by both the conservation and development sectors. Our ability to conserve ecosystems that people rely on is often limited by a lack of spatially explicit data on the location and distribution of ecosystem services (ES), the benefits provided by nature to people. Thus there is a need to map ES to guide conservation investments, to ensure these co-benefits are maintained. To target conservation investments most effectively, ES assessments must be rigorous enough to support conservation planning, rapid enough to respond to decision-making timelines, and often must rely on existing data. We developed a framework for rapid spatial assessment of ES that relies on expert and stakeholder consultation, available data, and spatial analyses in order to rapidly identify sites providing multiple benefits. We applied the framework in Madagascar, a country with globally significant biodiversity and a high level of human dependence on ecosystems. Our objective was to identify the ES co-benefits of biodiversity priority areas in order to guide the investment strategy of a global conservation fund. We assessed key provisioning (fisheries, hunting and non-timber forest products, and water for domestic use, agriculture, and hydropower), regulating (climate mitigation, flood risk reduction and coastal protection), and cultural (nature tourism) ES. We also conducted multi-criteria analyses to identify sites providing multiple benefits. While our approach has limitations, including the reliance on proximity-based indicators for several ES, the results were useful for targeting conservation investments by the Critical Ecosystem Partnership Fund (CEPF). Because our approach relies on available data, standardized methods for linking ES provision to ES use, and expert validation, it has the potential to quickly guide conservation planning and investment decisions in other data-poor regions.

## 1. Introduction

In recent decades, the conservation movement has increasingly focused on conserving ecosystems not only for their biodiversity values, but also for their role in providing benefits to people, known as ecosystem services (ES) [1]. This new focus has been driven by a recognition of the magnitude of ecosystem benefits to people and economies, as well as the consequences of their loss [2]. Governments, development agencies, and the private sector are also increasingly recognizing the importance of ecosystems in sustainability and development policies [3,4]. The United Nations Sustainable Development Goals, for example, explicitly recognize the role of ecosystems in supporting social and economic development [5].

In order to target scarce conservation resources to the places where they are most needed, decision makers need spatial information about where ES are produced and how they flow to people [6]. Numerous approaches for spatial assessment of ES have been developed for application in business [7], policy [8], and conservation [9] contexts. Many spatially explicit ES assessment tools have been developed, including InVEST [9], TESSA [10], ARIES [11], MIMES [12], Co$ting Nature [13], and for fresh water, WaterWorld [14], among others. Existing tools have varying requirements in terms of input data, time to conduct the assessment, and the level of specialized expertise required. Using these and other methods, ES assessments have been conducted at scales ranging from global [15] to national [16] and sub-national [17]. Many countries still lack spatial information about most ES, however [18]. Typically, information is available only for a few services and only at the sub-national scale [19]. Information on a broader array of provisioning, regulating, and cultural services, at a scale relevant for national-scale planning, is lacking in many countries.

ES assessments are inherently complex, due to the variety of biophysical characteristics and processes that produce services, tradeoffs between services, and the complexity of socioeconomic and political factors that influence ES use, such as access, equity, and values held by different beneficiaries [20]. At the same time, decision-making processes that could benefit from ES data tend to be rapid, with limited time and resources available for conducting assessments. Thus there is a need for ES assessments that represent the complexity of ES, at appropriate spatial scales, but are not so costly or time-consuming that they are impractical.

Existing rapid approaches to ES assessment are not usually tailored to the specific links between nature and people in a given context, which can make them less relevant for decision making. Tailored approaches are typically time consuming, requiring collection of primary data or sophisticated modeling. Our objective in this study was to develop an approach to ES assessment that strikes a balance between the inherent complexity of ES and the need for practical approaches. Unlike existing approaches, our approach strikes this balance by: 1) prioritizing a key set of ES to include in the analysis, tailored to the local context; 2) relying on available (global or national-scale) spatial data; 3) requiring rapid, relatively simple spatial analyses, and 5) providing easy-to understand products, including maps and tables, which rank sites based on their relative importance for ES. Our approach relies on expert and stakeholder consultation to identify the priority set of ES to be included in the analysis, the appropriate methods and data sets, and validation of results. As such, it represents a compromise between complexity and practicality.

We applied our approach to assess key provisioning, regulating, and cultural services provided by biodiversity priority areas in Madagascar, a biodiversity-rich country characterized by a high level of human dependence on ecosystems, in order to understand the potential co-benefits of conservation investments. This information was used to guide the investment strategy of the Critical Ecosystem Partnership Fund [CEPF]. CEPF is a global conservation fund that provides grants to civil society organizations for conservation within biodiversity hotspots–areas that harbor a significant fraction of global biodiversity as endemics, yet are highly threatened [21]. Between 2001 and 2012, CEPF invested USD 5.65 million in grants for biodiversity conservation in Madagascar [22]. In 2013, CEPF initiated an updated assessment to guide a five-year, USD 8.4 million investment strategy [22]. This level of funding is insufficient to conserve all of Madagascar’s biodiversity priority areas, which number more than 200, thus selection of priority sites was necessary.

To guide its investment decisions, CEPF develops an “Ecosystem Profile” that summarizes the biological, socioeconomic, and political context of each hotspot, and identifies priority sites for investment based on criteria including biodiversity significance, protection status, funding need, urgency of threat, and opportunity for influence by the civil society interventions [22]. CEPF Profiles must typically be completed in six to twelve months within a constrained budget. CEPF identifies biodiversity priority sites based on the Key Biodiversity Areas (KBA) methodology, using criteria related to vulnerability and irreplaceability [23]. In the past, information about ecosystem services has not been included in CEPF Profiles. Recognizing the importance of addressing the needs of local communities in its conservation investments, in Madagascar CEPF sought to incorporate information on ES benefits of KBAs into its site prioritization for the first time.

Historically, conservation efforts in Madagascar have been focused on large forest blocks [24]. In the past, biodiversity data has been used to inform conservation investment decisions, but ecosystem services data has not [16]. Existing research on ES in Madagascar generally focused on a limited set of services, such as carbon storage [25] or flood alleviation [26], or was limited to a single site [27]. Two studies, based on the same ES assessment, explored the links between biodiversity priority areas, hydrological services and biomass carbon [16,28]. These studies are insufficient to comprehensively assess multiple ES (e.g. hunting and non-timber forest products, fisheries, coastal protection, and tourism) at a national scale. Our analysis builds on this earlier assessment, adding several additional ES, new spatial analysis methods, more recent data, and expert validation. Our results were used to guide investment decisions by CEPF [22]. Our approach has the potential to be applied in other data-poor regions where rapid, spatially explicit ES assessment would inform conservation investment decisions.

## 2. Materials and Methods

**Fig 1:**
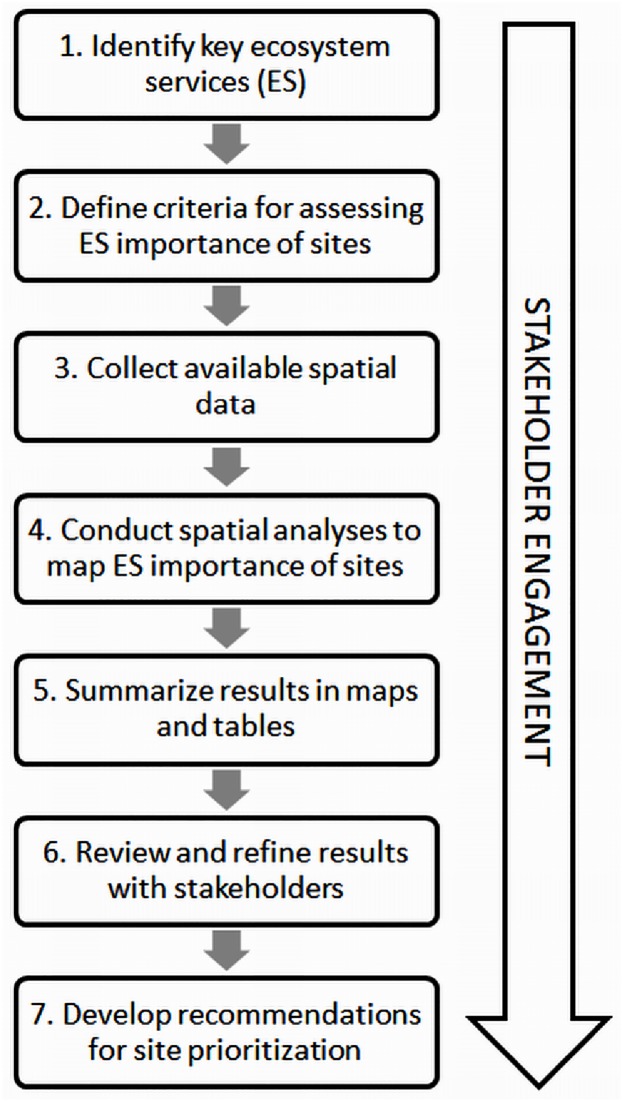
Steps for rapid ES assessment for site prioritization.

Our approach consists of seven steps (Fig 1), beginning with identifying the key ES that are relevant in a given context; defining criteria for assessing the importance of sites for ES; collecting available spatial data, conducting spatial analyses to map the importance of ES provided by sites; summarizing the results in a set of easy-to-understand maps and tables; reviewing and refining the results with experts and stakeholders; and making recommendations for site prioritization to guide conservation investments. These steps are consistent with existing frameworks for spatially explicit ES assessments [6,19,29]. Existing frameworks, including ours, stress the importance of engagement with experts and stakeholders at each step in the process. Stakeholders and experts such as representatives from local conservation and development NGOs, government agencies, research institutions, and local communities are critical for identifying key ES to include in the assessment, defining criteria for assessing the importance of sites for ES, providing relevant data, validating results, and applying the resulting information in decision making [30].

### Study area

**Fig 2:**
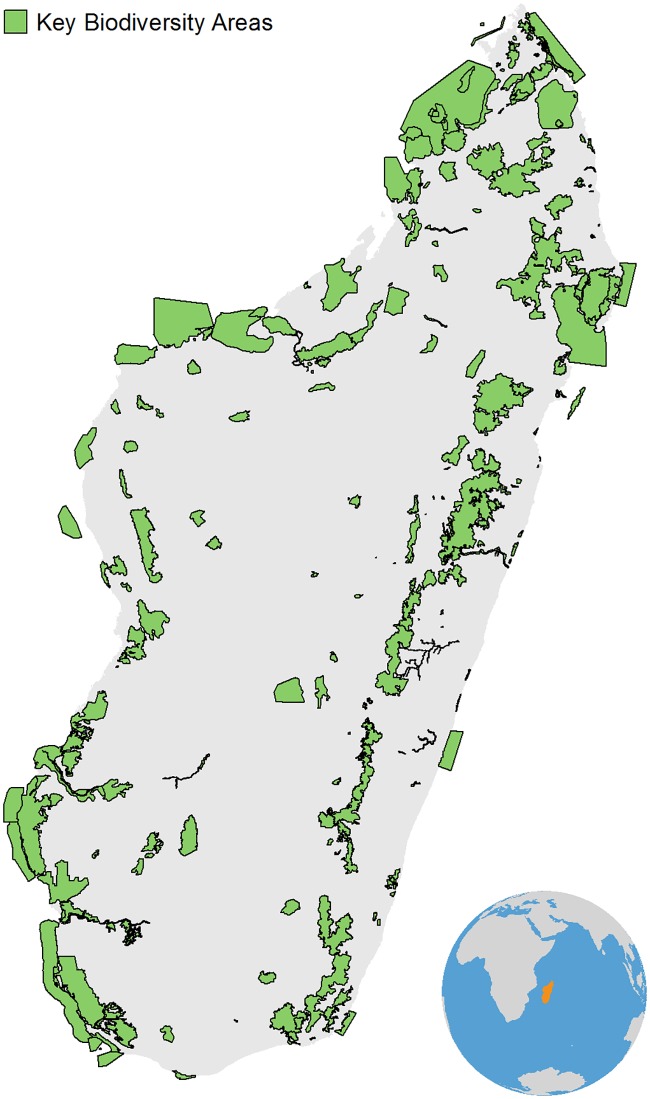
Key Biodiversity Areas (KBAs) in Madagascar.

The island nation of Madagascar, with an area of 592,040 km^2^, makes up most of the land area of the Madagascar and Indian Ocean Island Biodiversity Hotspot. It contains unparalleled numbers of endemic species which are highly threatened, making it a global conservation priority [21,31]. Over 200 biodiversity priority sites (KBAs) were identified during the development of the CEPF Ecosystem Profile, encompassing an area of 107,309 km^2^ including both terrestrial and offshore sites (Fig 2) [22].

In addition to their global significance, Madagascar’s wildlife and ecosystems are also important for its economy, human well-being, and national culture. Charismatic species such as lemurs have important cultural significance [32], and are also a major driver of tourism [33]. Madagascar has high rates of poverty, with over 80% of people living on less than USD 1.25 per day [34], therefore many people directly depend on natural ecosystems for food, water, fuel, fodder for livestock, and housing materials [35]. Coastal ecosystems such as mangroves and coral reefs support fisheries, protect coastal populations from cyclones, and support the cultural identity of certain coastal groups [36,37]. Rivers and ponds provide water for drinking and the production of rice, a critical part of the Malagasy diet [35].

Madagascar’s forests and the resources they provide are also an important part of the traditional culture of its people [38]. Forests also capture sediment and reduce erosion [39], and can reduce the severity of inland flooding [26]. Unfortunately, the nation’s forest cover decreased by almost 40% from the 1950s to 2000 [40], and the rate of loss has continued or increased since 2000 [41]. Forest loss causes erosion and sedimentation of the nation’s rivers and irrigation systems [39] and drives biodiversity loss [42,43]. Despite significant conservation efforts, habitat loss and degradation in Madagascar has continued, driven by poverty and food insecurity, and exacerbated by a severe political crisis from 2009–2014 [44].

### Stakeholder and expert engagement

While not a step in and of itself, stakeholder and expert engagement was critical for completion of all the other steps in the ES assessment process. We held two ES workshops in Antananarivo, Madagascar with eight local experts from conservation organizations, in September and November of 2013. The objective of the first workshop was to better understand the specific benefits provided to people by natural ecosystems in Madagascar, agree on a set of ES to include in the analysis, and come up with a proposed set of methods for the analysis. The second workshop was held to review and refine preliminary maps and results with feedback from the experts. We consulted with the experts periodically throughout the process, via e-mail and phone. Results from the ES analyses were also presented at a final national stakeholder consultation meeting which included 90 representatives from government, conservation and development NGOs, universities, and research institutions. These stakeholders included the key government agencies and NGOs who are collectively responsible for managing most of Madagascar’s KBAs as well as engaging with local communities in and around KBAs. Experts and stakeholders made suggestions which were used to refine the maps, such as recommending the use of more recent land cover data or adding an important irrigated rice production area to the maps. The ES-focused consultations were part of a much larger stakeholder consultation process led by CEPF, which involved approximately 200 people from 130 organizations and institutions (56 organizations from Madagascar) [22]. The broader consultation helped to identify and prioritize threats to biodiversity, underlying drivers of the threats, funding gaps and barriers to conservation success. See S2 Text for a list of stakeholder affiliations. Under our organization’s research ethics policy (S1 Text), workshops are not considered human subjects research, and therefore we were not required to seek approval from an ethics committee.

### 2.1 Identify key ecosystem services

Based on a literature review (S3 Text) and input from the first expert workshop, we identified a set of key ES in Madagascar (Table 1). Specific ES were identified based on their relevance in Madagascar; for example, rice is a staple food crop, and is highly dependent on surface water flows for irrigation; therefore, freshwater ES that support rice production were prioritized. The complete list of ES relevant in Madagascar include provisioning services related to food, fresh water, and energy; regulating services such as climate mitigation and regulation of flooding; and cultural services including nature tourism and spiritual values.

**Table 1:** Key ecosystem services in Madagascar.

Section	Division	Ecosystem Service
**Provisioning**	Nutrition	Animal products (fish, bushmeat)
		Plant products (edible plants, medicine)
		Water flows for household use
		Water flows for irrigation
	Materials	Construction materials (wood, thatch)
		Materials for artisanal products (wood, sedges)
		Water flows for mining
	Energy	Fuelwood, charcoal
		Water flows for hydropower
**Regulation & Maintenance**	Mediation of waste, toxics and other nuisances	Water quality for household use
		Water quality for irrigation
		Water quality for hydropower
	Mediation of flows	Flood regulation
		Drought regulation
	Maintenance of physical, chemical, biological conditions	Carbon storage and sequestration
		Coastal protection
		Genetic material
**Cultural**	Physical and intellectual interactions with ecosystems and land-/seascapes	Nature tourism
		Existence value (biodiversity)
	Spiritual, symbolic and other interactions with ecosystems and land-/seascapes	Cultural and spiritual identity

Key ecosystem services identified based on expert consultation and literature review; organized using the Common International Classification of Ecosystem Services (CICES) framework [45].

### 2.2 Define criteria for assessing ES importance of sites

From the complete list of ES relevant in Madagascar, we identified a sub-set of ES to be included in our spatial analyses, based on their perceived importance by local experts, their demonstrated importance in the literature, and the availability of adequate spatial data. This shorter list of services includes: hunting and non-timber forest products, commercial fisheries, small-scale fisheries, provision of fresh water, climate mitigation, coastal protection, inland flood regulation, and nature tourism. Our analyses focused on realized ES, which are the flows of ES that people are positioned to capture, either locally (e.g. fresh water) or globally (e.g. mitigation of climate change). We defined criteria for mapping areas of importance for each ES, based on the level of supply of the service, the level of demand for the service, or both. Level of threat to ecosystems providing ES was not included in our criteria for importance, as this was a criterion already included in prioritization of sites by CEPF. Criteria and methods used to assess importance for each ES is summarized in Table 2 and in the following sections. To inform site prioritization, we focused on the importance of each site relative to other sites within Madagascar. In all cases, the importance of sites varied continuously, from relatively more important to relatively less important. We did not conduct monetary valuation of ES due to data limitations, and because this was not required for site prioritization. Methods used to analyze each service are summarized briefly below, along with data limitations associated with each analysis. For additional details see S4 Text.

**Table 2:** Criteria and data used for spatial analyses of ecosystem services.

Ecosystem service	Criteria for importance	Spatial data (resolution) [source]
Hunting and non-timber forest products	Natural ecosystems within 10 km of relatively large numbers of food-insecure people, compared to other sites (ecosystems included in analysis: open water, mangroves, western dry forest, south western dry spiny forest-thicket, wetlands, western humid forest, humid forest, littoral forest, south western coastal bushland, western sub-humid forest, and tapia forest)	Land cover (30 m) [46]
		Population (1 km) [47]
		Food insecurity rates (commune level) [48]
Commercial Fisheries	Coastal and marine areas with higher landed fisheries catch values, relative to other sites	Landed fisheries catch values (0.5 degree) [49]
Small scale fisheries	Mangroves and coral reef ecosystems within 10 km of relatively large numbers of food-insecure people, compared to other sites	Mangroves (30 m) [50]
		Coral Reefs (500 m) [51]
		Population (1 km) [47]
		Food insecurity rates (commune level) [48]
Provision of freshwater	Areas providing high levels of water quantity (runoff) that were also located upstream of areas with high demand for water for domestic consumption (per capita water demand multiplied by population), rice production (per hectare water demand multiplied by hectares of rice), or hydropower (production capacity in megawatt hours used as a proxy for demand), compared to other sites	Modeled water flow (1 km) [14]
		Population (1 km) [47]
		Rice paddies (unknown) [52]
		Hydropower facilities (point shapefile) [JIRAMA, 2014 unpublished data]
Climate mitigation	Forests containing relatively high levels of biomass carbon stock (analysis 1) and vulnerable to deforestation (analysis 2), compared to other sites	Forest cover (30 m) [53]
		Biomass carbon stock (1 km) [54]
		Deforestation (30 m) [53]
Coastal protection	Mangroves within 2 km of relatively larger numbers of people vulnerable to cyclone storm surge, compared to other sites	Mangroves (30 m) [50]
		Population vulnerable to cyclone storm surge (1 km) [55]
Inland flood reduction	Areas providing higher levels of water quantity (runoff) upstream of areas with relatively large numbers of people vulnerable to flooding, compared to other sites	Modeled water flow (1 km) [14]
		Population vulnerable to flooding (1 km) [55]
Nature tourism	National parks with relatively high numbers of visitors, compared to other parks	Visitation in 2012 (per park) [Madagascar National parks 2012, unpublished data]

### 2.3 Collect available spatial data

The set of ES possible to analyze was constrained by the availability of spatial data. We collected and reviewed available data and selected those that were 1) consistent at a national scale, 2) had a spatial resolution fine enough to show sub-national variation (1 km^2^ or finer, when possible), and 3) most recently available (2005 or newer, when possible). We also reviewed computer-based tools for spatial ES assessment, in order to identify those that were feasible to apply given the constraints of our project. Criteria for feasibility included: 1) the tool models one or more of the key ES identified in our study area, 2) it is possible to apply the tool using available data, 3) the tool provides results at a national scale (in order to assess multiple sites simultaneously), and 4) the tool does not require extensive time or specialized expertise to apply. Cost was also a consideration; we focused primarily on tools which are free or low-cost for non-commercial users, with the exception of GIS software, for which free alternatives are available.

Tools included in our review included the Toolkit for Ecosystem Service Site-based Assessment (TESSA) [10], the Integrated Valuation of Ecosystem Services and Tradeoffs (InVEST) [9], Artificial Intelligence for Ecosystem Services (ARIES) [56], the Multiscale Integrated Model of Ecosystem Services (MIMES) [12], Co$ting Nature [13] and WaterWorld [14]. For a more complete review of ES assessment tools, see Bagstad et al. 2013 [56]. While all these tools have strengths, most did not meet our needs. TESSA is designed to be applied on a site-by-site basis, and was therefore not feasible to apply to the large number of biodiversity priority sites (over 200) in Madagascar. InVEST and ARIES have greater requirements for input data, and require more time to run than was feasible within the timeframe of our project (six months). ARIES and MIMES both require specialized expertise, including coding, and therefore also were not feasible for this analysis. Co$ting Nature met our requirements and was used for exploratory analyses. The tool does not allow customization, such as selection of specific ES that are relevant in a given context, and therefore wasn’t used for the final analyses. WaterWorld met all our criteria and therefore was used for the freshwater ES analyses. For other services, we conducted spatial analyses using GIS software including ArcGIS [57] and IDRISI Selva [58]. For certain key ES (such as cultural identity provided by ecosystems), we were unable to identify adequate existing data, nor computer-based assessment tools; therefore, the final set of ES we were able to include was limited to those which met these conditions.

### 2.4 Conduct spatial analyses to map ES importance of sites

#### 2.4.1 Hunting and non-timber forest products

We identified potential hunting and non-timber forest product (NTFP) supply areas using the locations of terrestrial and freshwater ecosystems from national land cover data [46]. The land cover classes that we included were: water, mangroves, western dry forest, south western dry spiny forest-thicket, wetlands, western humid forest, humid forest, littoral forest, south western coastal bushland, western sub-humid forest, and tapia forest. We estimated human demand for NTFPs based on global population data from LandScan [47] combined with national-level data on food insecurity [48]. We conducted a spatial analysis to identify ecosystems located within 10 km of populations of food-insecure people; this distance was based on estimated travel distances of people collecting NTFPs in Madagascar [59]. We assigned ecosystems near relatively higher numbers of food-insecure people higher importance for NTFPs than ecosystems near lower numbers or no food-insecure people. We were unable to analyze the specific products harvested, or the possible depletion of these in natural areas, due to lack of data. However, local experts reasoned that intact habitats which are near large populations of food-insecure people are both more important for provisioning services, and are also more threatened, and therefore should be prioritized for conservation investment. Global population data were used because they are the most recent available; the most recent national census available at the time of this analysis was from 1993.

#### 2.4.2 Commercial and small-scale fisheries

To identify areas important for commercial fisheries, we relied on global data on the landed values of commercial fisheries [49]. KBAs with higher average values for landed fisheries were assumed to be more important for commercial fisheries. Commercial fisheries data are known to underestimate the importance of small-scale (subsistence) fisheries in Madagascar, however. For example, total fish catch from 1950 to 2008 were estimated to be twice the volume reported by national fisheries agencies [60]. Therefore, to identify important areas for small-scale coastal fisheries, we identified areas of mangroves and coral reefs located near populations of food-insecure people. We used global data on the location of mangroves and coral reefs [50,51] and the same population and food insecurity datasets cited above for NTFPs. We assigned areas near relatively higher numbers of food-insecure people higher importance for small scale fisheries than areas near relatively lower numbers. We did not take into account the potential depletion of fisheries due to data limitations. Nationally consistent data are not available on fisheries in Madagascar, nor are data on mangroves and coral reefs, which is why we relied on global data.

#### 2.4.3 Provision of fresh water

To estimate the relative importance of KBAs for providing freshwater flows, we modeled annual water flows using WaterWorld, a web-based freshwater ecosystem service modeling tool [14]. First, we used the WaterWorld outputs and spatial analyses in a GIS to calculate the relative contribution of each 1 km^2^ grid cell to the overall surface water availability in each watershed. Second, we identified areas of water use for three primary users of water in Madagascar: 1) irrigated agriculture, 2) domestic (household) consumption, and 3) hydropower production. For each user type, we created an estimated water demand layer. For domestic (household) water use, we estimated the level of use of fresh water using population data from LandScan [47], multiplied by an average water use of 15.2 cubic meters per year per person (42.3 liters per day per person); this figure is based on household surveys from one city in Madagascar, and was the only published figure we could find [61]. We estimated water demand for irrigation using a national land use map of areas of irrigable agriculture [52]. Three agricultural land classes were included: 1) rice paddies, 2) monoculture crops, and 3) mosaic crops. We multiplied each 1 km^2^ cell of irrigable agriculture by an average water demand of 2000 mm per year, adjusted for annual rainfall; this figure is based on estimated water demand for irrigated rice from an earlier study [27]. We used hydropower dam production capacity data (megawatt-hours generated by each hydropower plant), provided by the national water authority, JIRAMA, as a proxy for actual water demand. The cumulative sum of demand for each water user was calculated, resulting in maps of cumulative water demand for domestic use, for agriculture, and for hydropower. Finally, we linked the areas providing freshwater flows generated in the first step to the cumulative water demand maps, using information on surface water flow directions obtained from HydroSHEDS [62] and a multiplication function. This final step resulted in three maps that show areas of relatively higher importance for freshwater provision for domestic, irrigation, and hydropower uses.

#### 2.4.4 Climate mitigation

We used global data on biomass carbon stocks [54] and national forest cover data [53] to identify forest areas with high quantities of carbon, relative to other sites. We also identified the high-carbon stock areas most vulnerable to deforestation by estimating future deforestation based on recent deforestation from 2005 to 2010 [53]. Forested areas near areas with higher rates of recent deforestation were assumed to be vulnerable to future deforestation. National data on biomass carbon stocks was not available at the time of this analysis, but could be used for future analyses.

#### 2.4.5 Coastal protection and inland flood reduction

We used global data on the location of mangrove ecosystems [50] and people vulnerable to flooding from coastal storm surge [55] to identify mangrove areas within two kilometers of vulnerable people. We chose two kilometers as a threshold based on studies that show declining, non-linear effects of distance from mangrove edge to shoreline on reducing wave height [63]. We omitted mangrove patches smaller than one hectare, as patch size also influences the ability of ecosystems to mitigate storm surge [63]. We assigned KBAs containing mangroves near relatively larger numbers of vulnerable people higher importance for coastal protection. We recognize the inherent uncertainty in our results due to the complex, non-linear interactions of mangrove structural characteristics, wind and wave action, coastal geomorphology, and other factors which influence the ability of mangroves to provide protective services [63].

To identify areas providing inland flood regulation services, we linked areas providing relatively larger amounts of water flows (based on modeling using WaterWorld and HydroSHEDs, see above) to the location of people vulnerable to flooding [55]. The location of vulnerable people was used to create a demand layer for flood regulation services. We also mapped forested areas within these more flood-prone areas [53]. We assigned KBAs as more important for flood regulation when they a) contained forests; b) contributed relatively more to water flows in a given watershed; and c) were located upstream of relatively larger numbers of vulnerable people. Again, we recognize the limitations of this analysis given the uncertain and complex dynamics that influence a forests’ ability to reduce flooding [26].

#### 2.4.6 Nature tourism

To estimate the tourism benefits of KBAs, we used park visitation data provided by Madagascar National Parks (unpublished data) as a proxy for overall importance for tourism. At the time of this analysis, data were available from 32 parks throughout the country for the time period 2000–2010 (average visitation) and 2012. No data on visitation are available for the rest of Madagascar’s KBAs, as this data is only collected in legally designated parks. Experts indicated that nature tourism in Madagascar is centered on the national park system; therefore, the park visitation data is thought to represent the importance of sites for nature tourism relatively well.

#### 2.4.7 Multi-criteria analysis

We conducted a multi-criteria analysis (MCA) [64] to identify areas that were important for multiple ES. We conducted a weighted linear combination MCA that included the terrestrial and freshwater ES (hunting and non-timber forest products, fresh water, climate mitigation, and nature tourism.) Each map layer representing a single ES was first rescaled to values ranging from 0 to 255. We then assigned weights to each layer based on input from the expert workshops. We tested different weightings (including equal weightings) to see how they affected the results (S5 Text). The ES we included, and their assigned weightings, are summarized in Table 3 as MCA1. Biomass carbon stocks, hunting and non-timber forest products, and fresh water services (collectively) were assigned equal weights; nature tourism was assigned a lower weight because the spatial extent of this dataset does not match the other services (data exist only for national parks). Because coastal and marine services had different spatial extents from one another and from the other services (coastal protection and small-scale fisheries were restricted to mangroves and coral reef habitats, commercial fisheries included offshore areas), it did not make sense to include them in an MCA. We conducted a second multi-criteria analysis, MCA2, excluding biomass carbon stock (a global service) to assess those ES that more directly support local communities. In testing (S5 Text), we found that equal weighting of fresh water services and NTFPs resulted in very high values near population centers in the eastern humid forests of the highlands. This pattern was driven by the NTFP results, which were in turn driven by population as habitats near larger populations of food-insecure people were given more importance. A more geographically dispersed result was desired for site prioritization by CEPF, as they wished to identify priorities in dry forest habitats in western Madagascar. Therefore, for MCA2 we assigned a higher weight to freshwater services, followed by NTFPs, then nature tourism. This resulted in a map with priority areas that were more geographically dispersed.

**Table 3:** Summary of ecosystem services included in the multi-criteria analyses.

Service	Weight	
	MCA1	MCA2
Biomass carbon stock (tC)	30	0
Hunting and non-timber forest products (# of food insecure people within 10km of terrestrial & freshwater ecosystems)	30	30
Relative importance for fresh water for domestic use	7.5	15
Relative importance for fresh water for irrigation	7.5	15
Relative importance for fresh water for hydropower	7.5	15
Relative importance for fresh water for flood protection	7.5	15
Nature tourism (# of visitors to Madagascar National Parks in 2012)	10	10
**TOTAL**	**100**	**100**

Different weights were applied in MCA1 and MCA2; see S5 Text for details.

### 2.5 Summarize results in maps and tables

We overlaid all the resulting maps with maps of KBAs. For each ecosystem service, we calculated an average value of the service provided by each KBA. We ran the same calculations for MCA1 and MCA2 results.

### 2.6 Review and refine results with stakeholders

As described above, results from the spatial analyses were presented at a national stakeholder consultation meeting which included 90 representatives from multiple institutions [22]. The stakeholders reviewed the analyses and where possible, validated the maps and other results. For example, fisheries experts were able to verify that sites which the spatial analyses indicated were important for commercial fisheries were indeed important based on their own knowledge. See S2 Text for a list of stakeholder affiliations.

### 2.7 Develop recommendations for site prioritization

We ranked all KBAs in a set of tables, and color-coded them on maps, to indicate their relative importance for each individual ES, and for multiple services as indicated by the multi-criteria analyses. To identify KBAs presenting the best opportunities for investment, CEPF used the results of our analyses alongside other factors including biodiversity significance, protection status, funding need, urgency of threat, and opportunity for influence by the civil society interventions [22].

## 3. Results

Results from our analyses are summarized below, with examples of maps from selected analyses; see S4 Text for a complete set of maps and S1 Table for detailed tabular results.

### 3.1 Provisioning services

**Fig 3:**
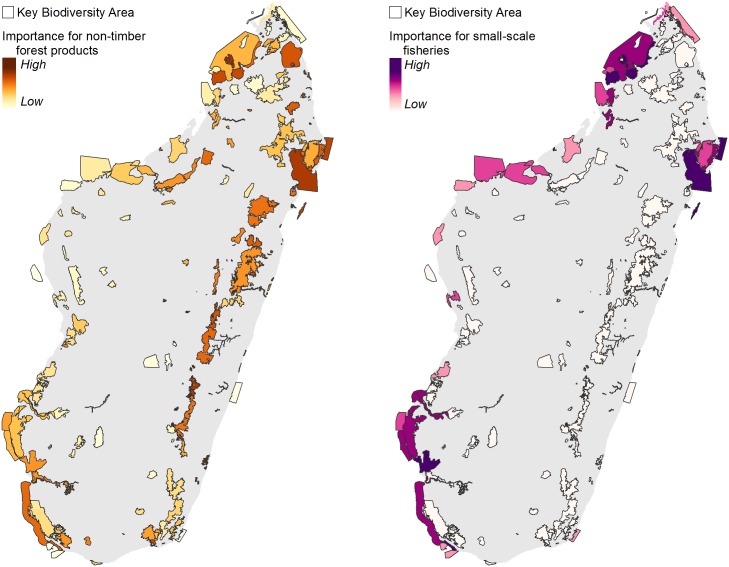
Importance of KBAs for hunting and non-timber forest products (left), and small-scale fisheries (right).

We found that most of Madagascar’s KBAs (193 out of 221) contain natural ecosystems within 10 km of food-insecure people, and are therefore likely providing important sources of hunting and non-timber forest products, such as game animals, fuel wood and edible plants to these populations. KBAs in the east and north are near the largest numbers of such people (Fig 3). Additionally, 42 coastal/marine KBAs contain coral reef or mangrove ecosystems that likely serve as important sources of fish and marine products for nearby food-insecure populations (Fig 3). We also found that 21 coastal/marine KBAs are important for commercial fisheries, with certain KBAs in the northeast, northwest, and west of Madagascar exhibiting higher values of landed fish (S4 Text).

**Fig 4:**
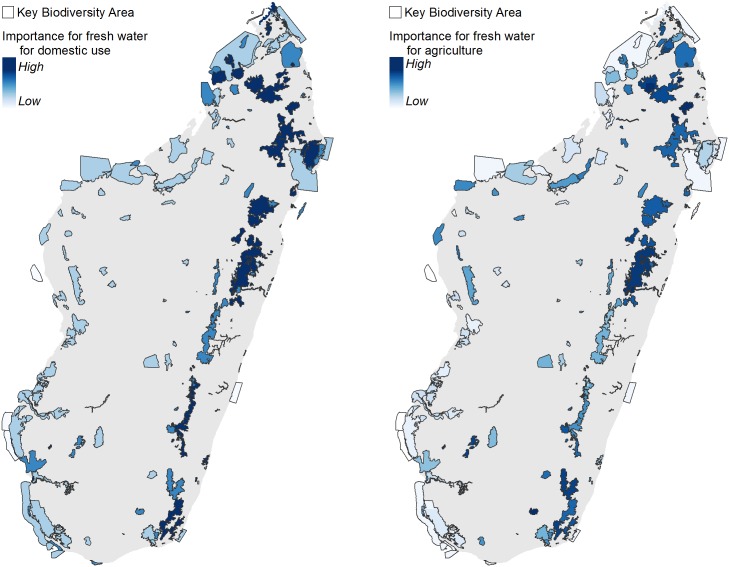
Importance of KBAs for fresh water for domestic use (left), and agriculture (right).

In terms of provision of freshwater flows, the majority of KBAs in Madagascar (203 of 221) are upstream of people and are therefore providing sources of water for drinking and other domestic uses (Fig 4). KBAs in the eastern highlands, upstream of the largest numbers of people, had the highest importance. While scarcity was not a criterion for importance, it should be noted that sites in the arid northeast and southwest, where water is scarce, are also very important to the people that live there, even if they appear less important at a national level. Many KBAs (184 out of 221) are also upstream of irrigated agricultural areas. Those with the highest relative importance are again located in the eastern highlands, as this is where the largest area of irrigated rice is located (Fig 4). There are also important areas in the east, north, and west of Madagascar; these drier regions, while more sparsely populated, also contain large areas of irrigated rice. Thirty-eight KBAs are upstream of hydroelectric dams (S4 Text); KBAs in the east, north, and northwest are relatively more important, based on the quantity of water flows and the production capacity of the dams.

### 3.2 Regulating services

**Fig 5:**
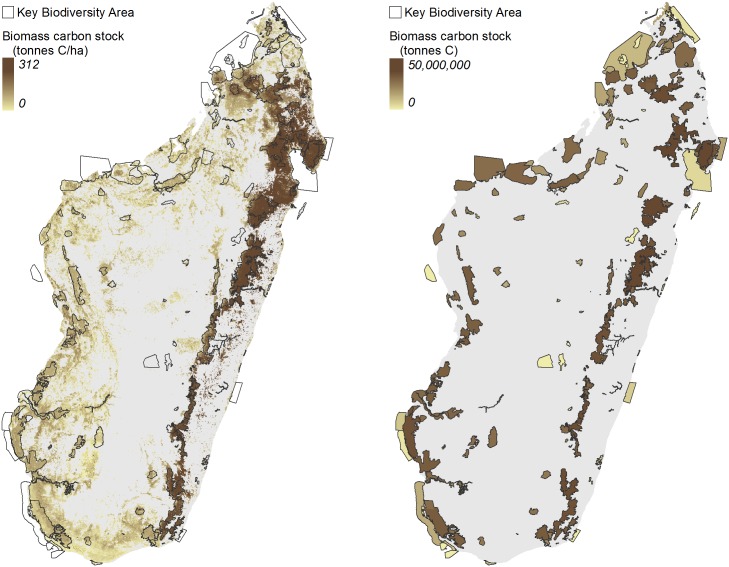
Forest carbon density (tons carbon per hectare) overlaid with KBAs (left); total forest carbon per KBA (tons C) (right).

Virtually all of Madagascar’s remaining forests occur within KBAs; thus all forested KBAs (180 out of 221 total KBAs) contain relatively high levels of biomass carbon stock. KBAs containing humid forests, concentrated in the eastern highlands, have the highest biomass carbon density, measured in tons of carbon per hectare (Fig 5). Of the forested KBAs, over half (92 of 180) experienced deforestation from 2005–2010 (S4 Text). If future deforestation is stopped, these sites have the highest potential for avoiding future emissions from deforestation.

In Madagascar, cyclones primarily hit from the east and north [65]; however mangroves exist primarily in the west. Nonetheless, 28 coastal KBAs contain mangroves within two kilometers of populations vulnerable to coastal storm surge, and therefore likely provide protection from coastal flooding (S4 Text). In terms of inland flood regulation, our analysis indicates that 123 out of 221 terrestrial KBAs, particularly those in the eastern and northeastern highlands, contain forests which likely provide flood regulation benefits to vulnerable people downstream.

### 3.3 Nature tourism

KBAs with the largest number of visitors from 2000–2010 include: Isalo National Park (averaging approximately 27,000 visitors per year), Andasibe-Mantadia National Park (19,000 visitors per year), Ranomafana National Park (18,000), Montagne d’Ambre National Park (13,000), and Ankarana Special Reserve (8,000).

### 3.4 Multi-criteria analysis

**Fig 6:**
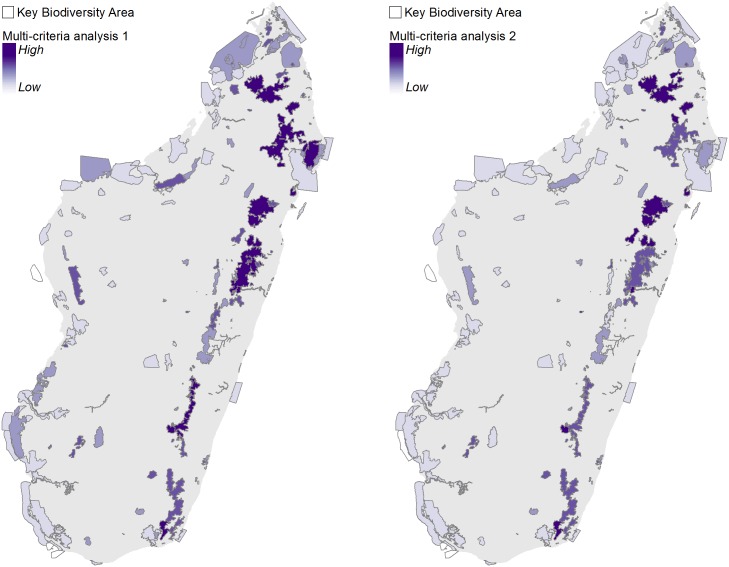
Results from multi-criteria analysis 1 (MCA1, left) and MCA2 (right). MCA1 includes carbon, hunting and non-timber forest products, fresh water, and nature tourism; MCA2 includes the same variables with the exception of carbon.

The first multi-criteria analysis (MCA1) highlighted KBAs in the northeast and eastern highlands as having the highest values for multiple ecosystem services, with additional high-value areas in the northwest and southwest (Fig 6). Results of MCA2 (which excluded forest carbon to focus on ES that benefit local people) were very similar, with slightly less emphasis on the eastern highlands, due to the exclusion of high carbon stock forests in these areas (Fig 6). For additional results see S5 Text.

### 3.5 Site prioritization

**Fig 7:**
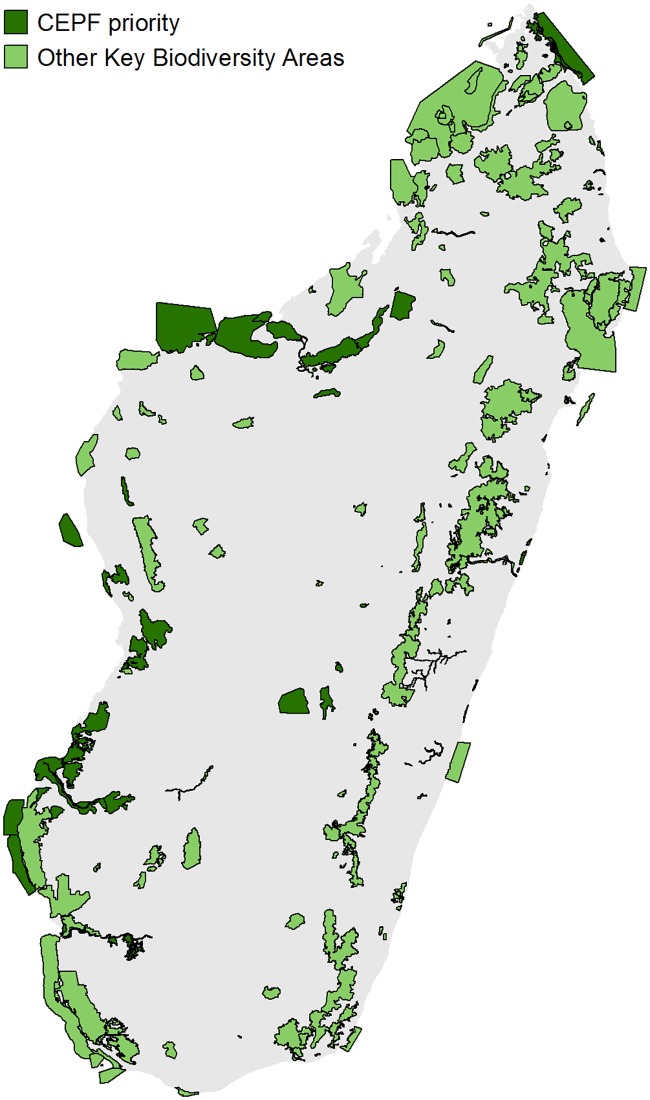
Final CEPF priority sites and other Key Biodiversity Areas. Sites in the eastern humid forests were not prioritized by CEPF, as these sites already have some level of conservation investment.

The stakeholder consultation and site prioritization process led to an agreement to focus CEPF investments on 39 sites, with a focus on three types of habitats: terrestrial wetlands and water bodies, western dry forests, and coastal and near-shore marine areas (Fig 7) [22]. These habitats are important for biodiversity and ES, yet have received relatively little conservation attention when compared with humid forests in the eastern highlands [66].

**Fig 8:**
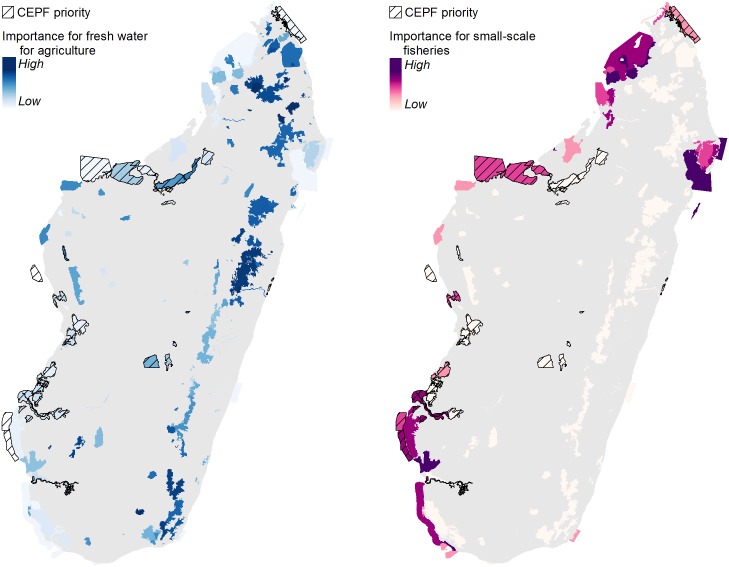
CEPF priority sites and relative importance for freshwater for agriculture (left) and local fisheries (right).

The ES analysis demonstrated the importance of these habitats for human well-being and livelihoods. While the contribution of these habitat types to global ES (carbon stock) is limited when compared to the eastern humid forests, their local importance for Madagascar’s population, particularly for food insecure and vulnerable coastal populations, was made clear by the ES analysis. For example, ES considerations led CEPF to prioritize conservation of fish stocks through sustainable management of coastal ecosystems for populations along Madagascar’s western coast, which are heavily dependent on protein from the sea (Fig 8). The ES maps were therefore used by CEPF during the stakeholder consultation to refine the selection of sites and select the sub-set of priority KBAs for CEPF investment within the three focal habitat types.

For example, our analyses of freshwater ecosystem services helped identify several priority river basins for CEPF investment in western Madagascar (Fig 8). Our analyses of small-scale coastal fisheries was also an important consideration for the selection of specific priority coastal and marine sites along the western coast (Fig 8). For example, a group of sites in southwestern Madagascar (Mikea landscape) were prioritized in part due to the presence of mangroves, which provide protection against cyclones and are an essential element for the resilience of local communities. Nearby marine areas are among the most important in Madagascar in terms of fish and seafood production. These sites were considered potential opportunities for conservation action by local civil society organizations, who are well-positioned to raise awareness and improve the sustainability of small-scale fishing. Terrestrial forests in the Mikea landscape also have relatively high carbon stocks, especially when compared to other dry western forests, and are threatened by relatively high rates of deforestation. Finally, forests and wetlands in the area play a role of regulating water supply for household use and agriculture in this densely populated region. For more details on the ways in which ES considerations informed site prioritization, see CEPF 2014 [22].

## 4. Discussion

Our approach provides a spatially explicit, data-driven ranking of the importance of sites for ES benefits. It strikes a balance between complexity and practicality by identifying relevant ES and beneficiaries to analyze, relying on available data, and providing results in easy-to-understand formats. Unlike site-based tools such as TESSA, our approach can be applied at a national scale, enabling assessment of multiple sites simultaneously. Unlike rapid ES modeling tools such as Co$ting Nature, our framework is designed to be tailored to the specific set of ecosystem services, beneficiaries, and data available in a given country or region, and incorporates stakeholder consultation to ensure local relevance. It relies on the best available spatial data, which is sometimes insufficient for application of tools such as InVEST. Finally, unlike more sophisticated modeling approaches such as ARIES or MIMES, our framework can be applied within a relatively constrained time frame (six to nine months) by team that has expertise in geographic information systems (GIS), but does not require specialized expertise in ES modeling. Because all of our analyses relied on global or commonly available national-scale data (e.g. land cover, population, socioeconomic data from census), models that rely on global data (e.g. WaterWorld), and relatively simple spatial analyses, they can be applied in other countries. In total, development and application of our ES assessment in Madagascar required approximately 155 days of staff time, divided amongst 10 people, with a budget of approximately USD 125,000. Our analysis relied on a network of in-country experts as well as a complementary process led by CEPF to identify KBAs using biological criteria and stakeholder consultation; the time and resources required for the KBA identification was not included in the above totals.

Our results indicate that all KBAs in Madagascar provide benefits to people, however different sites are important for different services and beneficiaries. Coastal and marine ecosystems provide fish stocks for small-scale and commercial fisheries. Mangrove and coral reef ecosystems also protect coastal populations from storms. Both dry and humid forests harbor charismatic wildlife and provide important recreational, tourism, and cultural values. The humid forests of the eastern highlands are important for climate mitigation, flood control, and provision of fresh water for household use, rice production, and generation of hydropower. Dry forests and wetlands in the arid north and west of the country are also critical for provision and regulation of fresh water in these water-scarce regions.

Identifying the co-benefits of biodiversity priority areas informed CEPF’s investment strategy, providing information about the overlap between biodiversity significance and benefits to people. In the absence of ES information, the important co-benefits of nature conservation for local people would not have been considered in investment decisions. This information is urgently needed, as Madagascar’s natural assets continue to be subjected to unsustainable levels of harvesting and degradation. Understanding the multiple benefits of biodiversity priority areas can help identify appropriate conservation interventions which are best suited to effectively manage these services, such as protection, community-based management, restoration, or payments for ecosystem services (PES) schemes. Identifying ecosystems that are near large numbers of food-insecure people, for example, could help managers promote sustainable levels of use through regulation, monitoring and enforcement. These ecosystems are also highly threatened, thus they were considered a high conservation priority by CEPF, both due to the services they provide and because, without intervention, they may be lost or degraded. Such areas are also critical for the people relying on them, therefore conservation efforts will have to balance local needs for food, water, and resources with long-term conservation goals. Areas that have already been converted or degraded could be prioritized for restoration, to increase their ability to reduce flooding, provide food and fuel, support biodiversity and associated tourism revenue, and store and sequester carbon.

Information about the importance of sites for ES should not be used as the sole criterion in site prioritization for conservation planning; additional criteria such as those used by CEPF (biodiversity significance, level of threat, existing investment, and opportunities for interventions) should be included in parallel with ES importance when final priority sites are selected. Also, our maps did not replace local knowledge of sites, but rather facilitated the process of identifying appropriate priorities among KBAs nationally. Stakeholders who participated in workshops reported that color-coding of the maps to indicate the relative importance of sites for ES made them effective for communication, even when there was limited time to present complex scientific information. Tabular results (S1 Table) were important references during and after the group discussions.

Because our maps of ES cover the entire country, they can be used for other analyses, such as measuring the contribution of protected areas to the provision of ES, and identifying important areas for ES that are currently unprotected. While our approach was tested in Madagascar, it can be applied anywhere there is a need for rapid, spatially explicit information about ES. We have applied the approach at the national scale in Cambodia, and across a nine-country region in Amazonia (unpublished data).

Our analysis has several limitations. Due to lack of adequate national-level data, we often had to rely on global sources, which may be inaccurate at finer spatial scales. Given more time and resources, we would have validated modeling results with primary data. For example, field sampling of forest biomass carbon would be required for more accurate assessment, such as those required for feasibility analyses for Reducing Emissions from Deforestation and forest Degradation (REDD) or carbon credit programs. Another key limitation was our reliance on proximity between ecosystems and people as an indicator of ES importance. Proximity might not be a good indicator in all cases; for example, local experts told us that fishers in Madagascar frequently travel long distances to catch fish, therefore proximity of marine habitat to human populations is not necessarily a strong indicator of the magnitude of the benefit provided. Primary data collection on the relationship between population, food insecurity, proximity to habitats, and other factors such as accessibility and sustainability of use would improve our spatial analyses. In addition, the results of our multi-criteria analysis were dependent on the weighting of the input layers (S5 Text). We recommend using MCA results with caution, in combination with maps of individual ES and local knowledge, for a more complete understanding of the importance of sites. Finally, our expert consultation focused on a small number of conservation scientists; given more time we would have consulted a broader set of experts such as social scientists, hydrologists, experts on food security, as well as representatives from local communities. The larger stakeholder consultation workshop, as well as a broader consultation conducted by CEPF as part of the KBA identification and final site prioritization process, was critically important for getting feedback and buy-in from a wider set of partners. Due to Madagascar’s large size and large number of KBAs, it was not feasible to consult with local communities in and around all of the sites. We relied on government and NGO representatives who engage with local communities to represent the local perspective; this influenced the final selection of services, for example, of those believed to be most important for local communities (e.g. provision of fish, hunting and non-timber forest products, and water) over those important at a global scale (e.g. carbon storage). Nonetheless, we recognize that any national-scale assessment will not adequately represent the interests of all stakeholders.

The lack of information about ES points to a broader need for collection of ES data, particularly in countries such as Madagascar, where many people directly depend on ecosystems. There is also a need for guidance to help project teams navigate the dizzying array of ES modeling tools that have been developed. Based on our review, we feel that toolkits like TESSA are appropriate at the site scale. Rapid, spatially-explicit ES modeling tools such as WaterWorld, Co$ting Nature, and InVEST show promise for providing information about ES across multiple sites, to support decision making. With more time, expertise, and data, ARIES and MIMES can provide more granular information about the complex temporal and spatial dynamics of ES. Even the best available analytical tools must be applied with consideration of the local context and in consultation with experts and local stakeholders, however.

## 5. Conclusions

There is a growing demand for rapid, low-cost approaches to ES assessment to inform conservation investment decisions. The specific suite of ES that are relevant in a given context will vary from place to place, as will the beneficiaries who rely on them. We have derived an approach that combines a tailored selection of ES based on the local context, existing global and national data for spatial analyses, and stakeholder consultation for review and validation of the results. Our approach is relatively rapid, detailed enough to support decision making, but has minimal requirements for local data and specialized expertise. Our approach is also limited by the lack of primary data on many services. In the Madagascar case, it provided the best available information to planners tasked with rapidly guiding conservation investments to the places where they could have multiple benefits. Without information about ES, site prioritization would have been conducted without consideration of the benefits KBAs provide to local people, which could have resulted in decisions that were less acceptable to local communities. Guiding investments to sites that provide relatively high levels of co-benefits such as food and water security, protection from climate impacts, and important recreational values will help ensure the ongoing provision of these benefits over time. Identifying and managing Madagascar’s natural assets can therefore support both conservation and sustainable development goals. Despite its limitations, we believe that rapid, spatially explicit ES assessment in data-poor contexts can provide valuable information about the links between nature and people, ultimately resulting in better outcomes for both.

## Supporting Information

S1 Table Tabular results. (XLSX)

S1 Text Conservation International Research Ethics Policy. (PDF)

S2 Text Expert consultation. (DOCX)

S3 Text Literature review. (DOCX)

S4 Text Detailed methods and results. (DOCX)

S5 Text Multi-criteria analyses. Results of different weights.
(DOCX)
