# Supplementary material for: Rapid Assessment of Ecosystem Service Co-Benefits of Biodiversity Priority Areas in Madagascar
Source: PLoS One. 2016 Dec 22;11(12):e0168575. doi: 10.1371/journal.pone.0168575 (PMC5179119; doi:10.1371/journal.pone.0168575)

CONSERVATION INTERNATIONAL

RIGHTS-BASED APPROACH
RESEARCH ETHICS POLICY

Document URL: tbd

Contact: Kristen Walker-Painemilla (kwalker@conservation.org)
Janet Edmond (jedmond@conservation.org)

I. POLICY SUMMARY

Conservation International (CI) strives to achieve excellence while pursuing its mission to empower societies to responsibly and sustainably care for nature, our global biodiversity, for the well-being of humanity. An integral part of CI's strategic plan is to link science and action to guide conservation of nature worldwide. CI is committed to creating a research climate that promotes faithful adherence to high ethical standards in the conduct of research and scholarship without inhibiting the productivity and creativity of persons involved in research. Research ethics is a cornerstone of public trust and critical for advancement to international prominence and excellence in research.

To further emphasize its commitment to excellence, CI has developed this "Research Ethics Policy" to ensure that research carried out and/or funded by the organization is subject to an appropriate ethical review and approval process.

II. PRINCIPLES

CI recognizes that research has the potential to impact, directly or indirectly, not only its subjects but also individuals or institutions discussed in the study, researchers, communities, and/or the sponsors of the research. The impact can be social, financial, physical or psychological.

Therefore, for all research involving human subjects and regardless of the source of funding, CI will apply the following principles:

1. The design and implementation of research studies must ensure integrity and quality;
2. Research participants, both staff and subjects, must be adequately informed of the purposes, methods and future uses of the results prior to the implementation of the study;
3. Research participants, both staff and subjects, must be adequately informed about the scope of the study, as well as its potential risks and benefits;
4. The researchers must respect the confidentiality of information provided by and the anonymity of the participants, unless otherwise agreed upon by the parties;
5. Participation in research studies must be fully free and voluntary, and consent must be obtained by researchers before implementation begins;
6. Researchers must avoid causing harm to the participants; and
7. Any conflicts of interest or partiality must be explicitly disclosed before the implementation begins.

III. COMMITMENT

To guarantee that research studies involving human subjects respect these principles, CI will implement an independent ethics review and approval process.

The objectives of this process are:

1. Identify and disclose potential direct or indirect research risks to human subjects;
2. Identify the appropriate risk avoidance and mitigation measures to be implemented by the study;
3. Obtain free, prior, and informed consent (FPIC) from research subjects;
4. Ensure confidentiality of personal information supplied by research subjects and the anonymity of respondents;
5. Identify and disclose any potential conflicts of interest;
6. Ensure investigator's accountability; and
7. Ensure that investigators and key personnel have completed training and secured certification on research ethics provided by accredited programs.

This process also fulfills current U.S. Federal Regulations[i] and requirements for funding provided by the National Science Foundation (NSF).[ii]

IV. ACTION

Effective January 1, 2013, every research study conducted and/or supported by CI that involves human subjects must undergo a review and approval process conducted by an independent Institutional Review Committee (IRC).

The review and approval process must be completed after funding for the research study has been secured but before the implementation of activities begins.

---

[i] The current U.S. federal policy on protecting human subjects in research comes from the Department of Health and Human Services (DHHS) and is codified as title 45, part 46 of the Code of Federal Regulations (45 CFR 46). This policy, also know as the Common Rule, defines human subject research and provides detailed requirements for the protection of human research participants for federally funded institutions.

[ii] Congress passed Public Law 110-69, The America COMPETES Act on August 9, 2007. This law mandates that institutions receiving funds from the National Science Foundation (NSF) provide appropriate training and oversight in the responsible conduct of Research for students (undergraduate and graduate) and postdoctoral researchers who are supported by the NSF.

V. KEY TERMS AND DEFINITIONS

1. **Research:** any form of disciplined inquiry that aims to contribute to a body of knowledge or theory.[iii]

Examples of research activities:

a. Formal investigations, including socio-economic surveys

b. Pilot research projects

c. Exploratory studies

d. Student studies, theses, or dissertations

e. Some demonstration activities (see next section for details)

Examples of activities that are generally NOT research:

a. Training courses and workshops

b. Conservation and/or development field demonstration projects and programs

2. **Human subject:** a living individual about whom an investigator conducting research obtains: a) data through intervention or interaction with the individual; or b) identifiable private information.

a. Intervention: includes both physical procedures by which data are gathered and manipulations of the subject or the subject's environment that are performed for research purposes; communication or interpersonal contact between investigator and subject.[iv]

b. Private information: includes information about behavior that occurs in a context in which an individual can reasonably expect that no observation or recording is taking place, and information which has been provided for specific purposes by an individual and which the individual can reasonably expect will not be made public. Private information must be individually identifiable (i.e., the identity of the subject is or may readily be ascertained by the investigator or associated with the information) in order for obtaining the information to constitute research involving human subjects.[iv]

c. Research risk: the potential of research projects to create physical or psychological harm, discomfort or stress to human subjects (the participants). Research risks also include potential harm to the participant's social or occupational status, privacy, values and beliefs, and relationships with family members and the community as a whole.[v]

4. **Personal Identifiable Information (PII):** data elements that are associated with a specific individual and that can be accessed and used in such a way that the identity of the individual who submitted the PII is known.

[iii] Economic & Social Research Council (ESRC): Research Ethics Framework

[iv] U.S. Federal Common Rule [45 CFR 46.102(f)]

[v] CI must certify on all proposals to the National Science Foundation (NSF) that it has a plan to provide appropriate RCR training. To comply with this requirement, CI is requiring that all students and post doctoral researchers paid by salary, stipend, or grant funded by the NSF complete an approved RCR training program.

VI. RESEARCH CATEGORIES

For the purpose of this policy, human subject research studies at CI are classified into two categories:

1. **Exempt research:** this category includes human subject research studies that might not require full review and approval by the IRC. This category includes, but is not limited to, the following research activities:

    a. Research conducted in established or commonly accepted educational settings, involving normal educational practices;

    b. Research involving the use of educational tests or observation of public behavior where individuals cannot be identified; and

    c. Research involving the collection or study of existing data, documents, records, or specimens, if these sources are publicly available or if the information is recorded by the investigator in such a manner that subjects cannot be identified, directly or through identifiers linked to the subjects.

2. **Research requiring full IRC review and approval:** this category includes research studies that must undergo a full review and approval by the IRC. This category includes, but is not limited to, the following research activities:

    a. Research involving the collection and/or use of coded data, images or specimens in which the researcher does have access to a code or link to re-identify the source of the data, images or specimens;

    b. Research involving information collected for purposes other than the research being conducted (e.g. medical, school, or correction records);

    c. Research involving vulnerable groups (e.g. children and young people, people with a learning disability or cognitive impairment, or individuals in a dependent or unequal relationship);

    d. Research involving sensitive topics, such as beliefs and religion, behavior, illegal or political activities, abuse or exploitation, mental health, gender, or ethnic status;

    e. Research involving groups where permission of a leader or formally recognized authority is normally required for initial access to members (e.g. ethnic or cultural groups, native peoples or indigenous communities);

    f. Research involving access to records of personal or confidential information, including behavioral, genetic or other biological and medical information, concerning identifiable individuals;

    g. Research involving intrusive interventions (physical procedures and manipulation of subject's environment) or interaction with living individuals (interpersonal contact, surveys, and other forms of communication).

VII. PROCEDURES FOR COMPLIANCE

The responsibility for assuring compliance is a cooperative effort involving the principal investigator (PI), research assistants and key personnel, the Betty and Gordon Moore Center for Ecosystem Science and Economics (the Moore Center), the Institutional Review Committee (IRC), the Grants Policy and Management (GPM) group and the Human Resources (HR) Division. The following procedures define the roles and responsibilities of each of these parties:

1. **Principal Investigator (PI)**
   The PI is responsible for fostering an environment of and providing guidance in the ethical and responsible conduct of research for individuals under his/her supervision. The PI is responsible for assessing whether a research study qualifies as exempt research or must be subject to a full IRC review and approval process (see Decision-making Flow Chart). The PI is responsible for submitting the appropriate protocols to the IRC to obtain an exempt status or a full review and final approval to implement the research project.

   The PI is also responsible for submitting the IRC's certification of compliance with this policy and the training certification (see below for more information).

2. **Research assistants and key personnel**
   Research assistants (field staff, students, post doctoral researchers) and key personnel are responsible for completing the required training in a timely manner and availing themselves of opportunities to further inform themselves regarding the responsible conduct of research in their specific area of inquiry.

3. **Betty and Gordon Moore Center for Ecosystem Science and Economics**
   The Moore Center will be available to advise the PI on issues related to complying with the requirements of this policy. Upon receipt of a qualifying award, the Moore Center will remind the PI that they must comply with the requirements of this policy.

4. **Institutional Review Committee (IRC)**
   This is a multi-divisional, multidisciplinary and independent body charged with reviewing and approving research involving human subjects to ensure that their dignity, rights, and welfare are protected. The independence of the IRC is founded on its membership, on strict rules regarding conflicts of interest, and on regular monitoring of and accountability for its decisions.

5. **Grants Policy and Management (GPM)**
   GPM is responsible for providing oversight and monitoring compliance with this plan. GPM will perform routine audits of training records to help ensure institutional compliance with the RCR requirements.

VIII. PROTECTION OF PERSONAL IDENTIFIABLE INFORMATION (PII)

To ensure confidentiality of information and anonymity of human subjects, the PI must address in the full review protocol how PII will be treated, including but not limited to compliance with local data protection legislation as well as methods and timeframe for PII collection, retention, storage, de-identification, and final disposal. When local legislation is not available, the PI will be responsible for fully complying with CI's "Security of Research Subjects' Personally Identifiable Data Policy" (to be developed).

IX. REQUIRED TRAINING AND CERTIFICATION OF TRAINING

As part of this policy, CI requires that the PI, research assistants and all key personnel affected by human subject research studies complete an approved Responsible Conduct of Research (RCR) training program. Online training is available through the Collaborative Institutional Training Initiative (CITI). The CITI public access course in the Responsible Conduct of Research is available without charge to the research community through the following Website: www.citiprogram.org

The required training must be completed within the first three months of award or employment or earlier if circumstances dictate (e.g. short term agreement/employment).

Proof of completion of this requirement must be either uploaded in GEM under the "Compliance" tab for sub-recipients (external grants) or submitted to Human Resources. A copy of the certificate should be kept by the trainee and Science and Knowledge .

X. PROCEDURES FOR INSTITUTIONAL EVALUATION AND MONITORING

The IRC will establish the appropriate procedures to evaluate and monitor the implementation of research that has received ethical review and approval, throughout the life of the study. Procedures will be proportionate to the nature and degree of risks entailed in the research.

XI. COMPLAINTS PROCEDURES

CI will establish the appropriate mechanisms for receiving and addressing complaints or concerns about the research study.

XII. NON COMPLIANCE

Failure to comply may result in sanctions imposed by CI, such as restricting access to grant funds, or revocation of the award by the sponsoring agency.

XIII. FORMS AND PROTOCOLS

This policy is accompanied by the following forms and protocols (to be developed)

1. Determining if an activity is research
2. Determining if a research activity includes human subjects and the type of protocol needed
3. Consent form
4. Exempt research protocol
5. Full review protocol
6. Certification of compliance with this policy
7. Certification of training completion

XIV. RELATED LINKS AND SUPPLEMENTAL READING

- Economic & Social Research Council. Research Ethics Framework. United Kingdom.
- University of Wisconsin-Madison. Human Research Protection Program Policies – http://www.grad.wisc.edu/research/hrpp/HRPPpolicy.html
- The National Commission for the Protection of Human Subjects of Biomedical and Behavioral Research. The Belmont Report: Ethical Principles and Guidelines for the Protection of Human Subjects of Research.
- U.S. Department of Health and Human Services Guidelines for the Conduct of Research Involving Human Subjects in the National Institutes of Health. 2004
- Conservation International. Responsible Conduct of Research Policy. 2010
- Oxford Brookes University. Research Ethics – http://www.brookes.ac.uk/res/ethics/procedures

DECISION-MAKING FLOW CHART
REVIEW AND APPROVAL OF RESEARCH
INVOVLVING HUMAN SUBJECTS

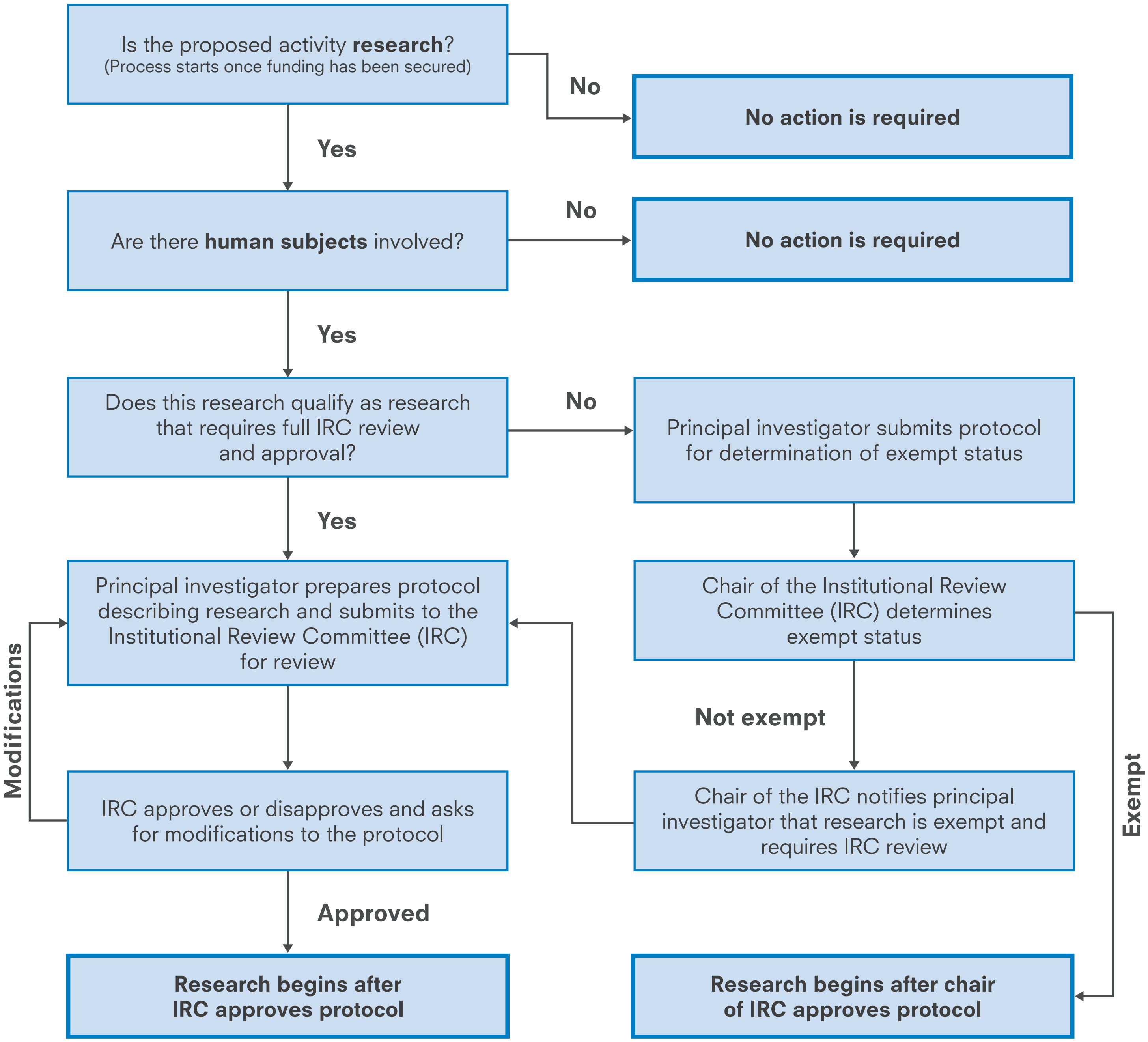

Supplement: S1 Text — (PDF) [file pone.0168575.s002.pdf]
