# Supplementary material for: Rapid Assessment of Ecosystem Service Co-Benefits of Biodiversity Priority Areas in Madagascar
Source: PLoS One. 2016 Dec 22;11(12):e0168575. doi: 10.1371/journal.pone.0168575 (PMC5179119; doi:10.1371/journal.pone.0168575)
Supplement: S2 Text — (DOCX) [file pone.0168575.s003.docx]

**S2 Text. Expert and stakeholder consultation**

We consulted with local experts from conservation organizations throughout this process, beginning with the collection of relevant literature, identification of relevant ecosystem services, methods for the desktop analyses (below), and collection of spatial datasets. We held two half-day expert workshops in Antananarivo on September 10 and November 18, 2013 to review the conceptual framework for the analysis, identify key ecosystem services for Madagascar, agree on methods for the desktop analyses, review preliminary results, and refine the analyses. Results from the ES analyses were also presented at a final national stakeholder consultation meeting which included 90 representatives from government, conservation and development NGOs, universities, and research institutions. Our organization’s research ethics policy prohibits us from publishing the names of the experts/stakeholders consulted during the workshops, as this is personally identifying information, however participant affiliations are listed below.

*Participants in local experts’ workshops, September 10 and November 18, 2013*

| **Participant** | **Institution** |
| --- | --- |
| 1 | Asity Madagascar |
| 2 | Rebioma, Wildlife Conservation Socieity |
| 3 | Conservation International Madagascar |
| 4 | Conservation International Madagascar |
| 5 | ARFIE |
| 6 | Rebioma |
| 7 | Conservation International Madagascar |
| 8 | Conservation International Madagascar |

*Participants in the CEPF Consultation Workshop: “Ecosystem Profile: Madagascar and Indian Ocean Islands Hotspot”, September 4 2013. Not all participants registered so we are missing affiliations for 17 participants.*

| **Participant** | **Institution** |
| --- | --- |
| 1 | Ministère de l'Eau |
| 2 | Ministère des Eaux et Forêts |
| 3 | Ministère des Eaux et Forêts |
| 4 | Ministère des Eaux et Forêts |
| 5 | Ministère des Mines |
| 6 | Ministère de la Météorologie |
| 7 | INSTAT |
| 8 | ANAE |
| 9 | ONE |
| 10 | Madagascar National Parks |
| 11 | SAGE |
| 12 | ARSIE |
| 13 | Université d'Antananarivo |
| 14 | Université d'Antananarivo |
| 15 | Université d'Antananarivo |
| 16 | Laboratoire de Recherche Appliquée (LRI) |
| 17 | REPC |
| 18 | Vahatra |
| 19 | Wealth Accounting Valuation and Ecosystem Services (WAVES) |
| 20 | Union Européenne |
| 21 | AFD |
| 22 | USAID |
| 23 | JICA |
| 24 | GIZ |
| 25 | FAPBM |
| 26 | Blue Ventures |
| 27 | Ambatovy |
| 28 | Alliance Voahary Gasy |
| 29 | ASG |
| 30 | Fondation Tany Meva |
| 31 | World Wildlife Fund |
| 32 | World Wildlife Fund |
| 33 | Wildlife Conservation Society |
| 34 | REBIOMA |
| 35 | Peregrine Fund |
| 36 | Durrell Wildlife |
| 37 | MAVOA |
| 38 | Missouri Botanical Garden |
| 39 | Missouri Botanical Garden |
| 40 | Royal Botanical Garden- Kew |
| 41 | Royal Botanical Garden- Kew |
| 42 | FANAMBY |
| 43 | Homme et Environnement |
| 44 | Homme et Environnement |
| 45 | ASITY |
| 46 | CAS |
| 47 | CAS |
| 48 | Comité Consultatif |
| 49 | Conservation International |
| 50 | Conservation International |
| 51 | Conservation International |
| 52 | Conservation International |
| 53 | Consultant |
| 54 | Consultant |
| 55 | Consultant |
| 56 | Consultant |
| 57 | Biotope |
| 58 | Naturevolution |
| 59 | Conservation International |
| 60 | Oceanographe |
| 61 | TSA |
| 62 | VIF |
| 63 | CE/VPDAT |
| 64 | Voahary Salama |
| 65 | CIRAD |
| 66 | IRD |
| 67 | C3EDM |
| 68 | Wildlife Conservation Society |
| 69 | GSDM |
| 70 | Comité Consultatif |
| 71 | Comité Consultatif |
| 72 | Comité Consultatif |
| 73 | Comité Consultatif |
| 74 | (Not stated) |
| 75 | (Not stated) |
| 76 | (Not stated) |
| 77 | (Not stated) |
| 78 | (Not stated) |
| 79 | (Not stated) |
| 80 | (Not stated) |
| 81 | (Not stated) |
| 82 | (Not stated) |
| 83 | (Not stated) |
| 84 | (Not stated) |
| 85 | (Not stated) |
| 86 | (Not stated) |
| 87 | (Not stated) |
| 88 | (Not stated) |
| 89 | (Not stated) |
| 90 | (Not stated) |
