# Supplementary material for: Rapid Assessment of Ecosystem Service Co-Benefits of Biodiversity Priority Areas in Madagascar
Source: PLoS One. 2016 Dec 22;11(12):e0168575. doi: 10.1371/journal.pone.0168575 (PMC5179119; doi:10.1371/journal.pone.0168575)
Supplement: S3 Text — (DOCX) [file pone.0168575.s004.docx]

**S3 Text. Literature Review**

There have been a number of assessments of ecosystem services in Madagascar at national and sub-national scales. There have also been numerous studies of the links between ecosystems and people in the region, even if they are not framed in the language of ecosystem services. We conducted a literature review of relevant publications to provide context for our assessment, as well as to identify existing information and analyses to be included in our current assessment. Our objective was to update these past analyses with more recent data, to fill gaps, and to use the results to inform the current CEPF ecosystem profile. Relevant articles were requested from key experts within MCSO, from CI Madagascar and partner organizations, and external researchers, or were identified using web searches (e.g. Google Scholar search for “climate adaptation Madagascar.”)

In total, we reviewed 125 articles, primarily scientific papers and some unpublished reports (see table below). These included numerous studies on themes relevant to ecosystem services: biodiversity (46), water (13), climate mitigation and deforestation (12), climate adaptation (13), food (34), cultural services (9), and human well-being and poverty (33). Many articles (45) included reference to multiple themes. Below we highlight several studies that addressed multiple ecosystem services at the national scale (considered most relevant for the current analysis), and a number of sub-national studies, many of which addressed only a single theme.

*Ecosystem Services and Human Well-being in Madagascar*

The people of Madagascar, particularly its rural and poorer populations, have a high level of dependence on natural resources and a strong relation to nature and environment (Kiefer et al. 2010). According to the World Bank, 50% of Madagascar’s wealth is in the form of natural capital (World Bank 2013). Overall GDP is driven by income from mining, tourism, and services. However, agriculture, livestock, and fishery sectors provide 95% of Madagscar’s food supply and source of livelihoods for 75% of the rural population (IISD 2011). Biodiversity and ecosystem services are therefore intricately linked to poverty, political stability, and other elements of well-being:

Madagascar’s economy depends to a great extent on exported ecosystem goods such as seafood and spices, and increasingly on minerals derived by extractive industries... The condition and availability of biodiversity and ecosystem services seems to be interlinked with political stability... Global environmental and socio-economic changes, such as climate change or high population growth rates, increasingly have an influence on human wellbeing, which makes the access to, and availability of, ecosystem services a major concern. The integrity of biodiversity, hence, contributes to the extent of vulnerability of Madagascar’s population and the reduction of dependences and poverty (Kiefer et al. 2010).

The poverty rate is very high, and increased from 70% (1993) to 77% (2010). Rural poverty is even higher (82.2%) in part due to environmental deterioration, exacerbated by the current political crisis. GDP per capita was well below the average of the poorest sub-Saharan countries in 2009 (Rabarison 2013). The households that have the most persistent poverty are largely not members of the dominant ethnic group; are land poor; live in remote areas; and are headed by uneducated individuals, most commonly women (Stifel et al. 2010). Poverty drives unsustainable land and natural resource use. According to one study, “Small scale agricultural households were hit particularly hard in the 1990s, and the data suggest that these are the very households that have been extending their land use by clearing and cultivating increasingly fragile lands” (Paternostro et al. 2001). These practices are driving the conversion and degradation of the very ecosystems that the Malagasy people depend upon.

*National-scale ecosystem service assessments in Madagascar*

Two studies have explored ecosystem service values at the national scale, with a specific focus on the links between ecosystem services and biodiversity priority areas. They are therefore the most relevant for this current analysis. For example, there is an existing assessment of the relative priority of unprotected KBAs based on data on human related threats, ecosystem services, and biological values (Rogers et al. 2010). The study focused on 70 KBAs that were unprotected at the time. The authors found that sixteen key biodiversity areas emerged as particularly important for both biodiversity and ecosystem services. This assessment focused only on hydrological services (provision of drinking water to downstream populations and irrigation of rice paddies), thus our current KBA+ analysis substantially adds to this past work by including numerous additional ecosystem services.

The Rogers et al. (2010) assessment incorporated hydrological information from a more complete assessment that explored opportunities for bundling biodiversity conservation with carbon and water services (Wendland et al. 2010). (One of the authors of this report, Miroslav Honzák, was also a co-author of both the Rogers et al. and Wendland et al. studies.) The Wendland et al. (2010) analysis identified approximately 30,000 km^2^ of natural habitat (out of a total of 134,301 km^2^) that could potentially meet biodiversity conservation goals and also protect water and carbon services. Results of this analysis were incorporated into our current KBA+ analysis; however, the information on carbon was updated using more recent (2010) forest cover data, and the fresh water analysis was updated using a more sophisticated, process-based ecohydrological modeling tool (WaterWorld).

The Missouri Botanic Garden (2013) conducted a review of ecosystem service values of 51 Important Plant Areas (APAPCs for their French acronym), based on expert opinion. Results indicate that such sites provide a wide range of services to local communities: timber, wood for canoes and posts, fuel, food, materials for handicrafts, wild silk, traditional medicines, water for crop irrigation, cattle refuge, breeding grounds for fish and shrimp, social cohesion and identity, and religious values (Missouri Botanic Garden 2013).

Panegos (2011) conducted a review of ecosystem service values of Madagascar’s 46 protected areas, based on a literature review, interviews with park officials, and a review of planning documents, websites, and other materials. She assessed whether or not the protected areas were important for providing hydrological services, climate regulation, genetic reservoirs, ecological connectivity, scenic beauty, cultural value, and nature tourism. The role of water was highlighted in most of the protected areas (30 out of 46). Fourteen protected areas were considered important for nature tourism. Eleven for ecological connectivity, eleven for scenic beauty, eleven for cultural services, eight were considered genetic reserves, and six were presented as having a role in regulating climate. This analysis, while useful, is based on a combination of quantitative analyses (past ecosystem service assessments) and qualitative information based on subjective opinion (interviews, planning documents, etc.) The results are therefore difficult to interpret and not applicable outside the boundaries of existing protected areas.

An economic evaluation of the value of ecosystem services of Madagascar’s protected areas found that the national benefits of biodiversity conservation and nature tourism could be valued at US$5/hectare of protected areas per year (Carret and Loyer 2003). The same study found that water users downstream of protected areas (rice growers and consumers of drinking water) were willing to pay US$3 per hectare of protected area, increasing over time. This study is also useful but also does not apply outside of existing protected areas.

*Sub-national & thematic assessments*

There have been numerous studies about the relationship between nature and human well-being in Madagascar, many of them conducted at sub-national scales and around specific themes (such as the role of biodiversity in food security.) Below is a summary organized by thematic area: food, fresh water, climate adaptation, climate mitigation, and cultural values.

*Provisioning services: food security*

Food security is a major issue in Madagascar. Overall, 84 percent of households experience a time during the year when they don’t have enough cash or food (World Food Programme and UNICEF 2011). The Southern region of Madagascar showed the highest prevalence of households (92 percent) reporting this problem, followed by the West‐South Western region (89 percent). The national population growth rate is 2.8% (Rabarison 2013), twice the rate of India, meaning every year there are even more hungry mouths to feed. There has been an overall decline in fish stocks and agricultural production, making it even harder for Malagasy people to feed themselves (IISD 2011). Natural ecosystems play a key role in food security, by providing wild sources of food (fisheries, e.g. Le Manach et al. 2012, and wildlife hunting, e.g. Brashares et al. 2011) as well as services that support agriculture, such as fresh water for irrigation (e.g. Bakoariniaina et al. 2006), soil quality, climate regulation, pest and pathogen control, and pollination (e.g. Bodin et al. 2006).

The Malagasy diet is based mainly on rice (on average, rice is consumed 6.2 times a week), vegetables (4.4 times a week) and tubers (mainly cassava, 3.9) (World Food Programme and UNICEF 2011). Vegetable and animal proteins are rarely consumed (once and 2.3 times respectively). Fish is the most popular animal protein, with an average weekly consumption of 1.3 days. The Southern zone appears to have the poorest diet. Here households eat cassava almost every day (6.1), followed by cereals, and vegetables (2.8).

*Fisheries*

Wild food sources, including fisheries, bushmeat, and wild plants, are critical to food security in Madagascar. One third of the population (34%) lives within 100 km of the coast (Rabarison 2013). It has been estimated that fish and fish products contribute about 20 percent of animal protein consumption of the total population (FAO 2008). The fisheries sector also plays a major socio-economic role in the country. Fisheries including aquaculture contribute 7 percent to the gross Domestic Product (GDP) and is also a provider of employment (FAO 2008). Approximately 194,000 direct jobs in the primary sector of which 33,365 as fish farmers and 3,000 indirect jobs have been created. In rural communities, fishing is the main source of income.

Thus coastal and marine fisheries provide critical sources of food as well as livelihoods, but are threatened by overharvest (Le Manach et al. 2012). Shrimps have been exploited industrially since the mid-1960s, while other invertebrates (notably octopus, lobster, crab and sea cucumber) and sharks are exploited for subsistence or on a semi-industrial scale. Sea turtles and small fish species are caught by small-scale fishers for local consumption. Several species of tuna (e.g., yellowfin Thunnus albacares, big-eye Thunnus obesus) are heavily targeted by illegal Asian and legal European fleets (Le Manach et al. 2012).

Small-scale fisheries are of fundamental importance to coastal communities, especially in the arid south-western region of the country where agriculture is largely unviable, and where many communities rely on food assistance (Le Manach et al. 2012). Data on small scale fisheries is limited and therefore the importance of this resource has historically been unreported or underestimated. The reconstruction of total catches by all Malagasy fisheries showed that total catches between 1950 and 2008 were twice the volume reported by national fisheries agencies, but signs of decline have been observed in several stocks (Le Manach et al. 2012). Recent studies suggest that traditional fishers are now migrating in unprecedented numbers to increasingly remote and isolated regions of the west coast as a coping mechanism in direct response to declining catches (Le Manach et al. 2012).

Data from a small-scale traditional invertebrate fishery in southwestern Madagascar showed that more than 34 taxa were caught, both for export and local consumption, but there are indications that the resources are being over-exploited (Barnes and Rawlinson 2009). A survey of 11 villages within the vicinity of Kirindy-Mite Marine Protected Area (Jones 2012) showed a high dependence on harvesting of marine resources, and low diversification of livelihood strategies. The traditional fishery of the Kirindy-Mite area is in marked decline, as a result of environmental stressors, such as cyclones and sustained high water temperatures leading to mass coral bleaching, as well as sustained fishing pressure from traditional, artisanal and industrial fishers. This situation leaves fishing communities of the Kirindy-Mite area highly susceptible to a potential future collapse of the traditional fishery.

Freshwater fisheries are also critical sources of food and income. Data from a RAMSAR site in western Madagascar, Manambolomaty Lakes, indicates that fisheries management has increased the annual local revenue by an estimated US$1,562 per fisherman per season, and the tax from the fish sales makes up 56% of the budgets of two local communes (Rabearivony et al. 2008). Endemic freshwater crayfish species are harvested for both subsistence use and small-scale trade, one study from eastern Madagascar indicated that more than half of households were directly involved in the harvest, contributing substantially to local incomes (Jones et al. 2006).

*Wildlife hunting & edible plants*

Tubers such as wild yams are considered a “famine food,” critically important during the lean season between rice harvests (Ackerman 2004, Damson et al. 2010). Wildlife hunting and consumption (bushmeat) has been shown to increase when alternative livelihoods collapse in Madagascar and several other African nations, providing a safety net in times of crisis (Brashares et al. 2011). However, this net only exists for people who live near harvestable wildlife, and only lasts as long as the wildlife isn’t hunted to extinction. Interviews with 1,154 households in 12 communes in eastern Madagascar showed that the majority of meals contain no animal protein, and bushmeat is not preferred over fish and domestic animals (Jenkins et al. 2011). Nonetheless respondents consumed a wide range of wild species; 95% of respondents had eaten at least one protected species, and nearly 45% had eaten more than 10. Traditional taboos have protected certain species, such as the Endangered Indri lemur, but there is evidence that such taboos are rapidly eroding. Wildlife consumption is also common in urban areas; a study from western Madagascar showed that bushmeat accounted for 10% of the meat consumed on a given day, including six wild mammals and five wild bird species, although fish and domestic animals were preferred and more affordable (Randrianandrianina et al. 2010). The authors state that it is likely that wildlife consumption is underestimated because of reluctance of interviewees to admit illegal activities.

Interviews from northeastern Madagascar revealed that 23 mammal species were hunted for consumption in that region (Golden 2009), but modeling results suggest that hunting is unsustainable. Evidence from a temporary camp in a national park in the northwest indicates that at least 49 unique wild animals were consumed for food, the majority of which are protected by law and are endemic to the island (Garcia and Goodman 2003). Even bats are consumed during periods of food shortage, but the level of collection surpasses the bats’ breeding potential, which will likely result in extirpation of local populations over time (Goodman 2006). Frogs are also commonly collected for domestic consumption in restaurants as well as overseas export, providing important income for individual hunters, but potentially impacting frog populations (Jenkins et al. 2009). A study from a single restaurant in eastern Madagascar showed a delivery of 3,233 frogs over a five-month period, or an average of 249 per week, including one IUCN-listed critically endangered species (Jenkins et al. 2009). Wildlife consumption has also been shown to be a critical source of nutrition; one study in northeastern Madagascar links wildlife consumption to a 29% decrease in children suffering from anemia, with associated long-term health benefits (Golden et al. 2011). However, long-term depletion of the wildlife would lead to extinctions and therefore a loss of this critical resource.

Overharvesting of wildlife can lead to ecological changes to the forest with broader impacts on the services provided by these ecosystems. Past lemur extinctions brought about by humans likely had significant ecological ramifications for the ecosystems of southern and southwestern Madagascar (Crowley et al. 2011), and future losses may have far-reaching ecological consequences including impacts on forest structure and dynamics (Moses and Semple 2011).

*Materials*

Natural ecosystems also provide critical sources of materials and fuel that indirectly support food security. The majority of Madagascar’s people are still reliant on wood from nearby forests for cooking and heating, even in the cities (Bertrand et al. 2010). Wood energy is used daily by more than 90% of the population and accounts for over 75% of primary energy consumption in the country, and is considered the cause of about 100,000 ha of deforestation (Ministry of Environment and Forestry, cited in Rabarison 2013).

Mangroves have particular importance for providing a diversity of materials in Madagascar (Jones 2013). A study from fishing areas along the northwest and western coasts shows that mangrove wood is used for making fishing traps, canoes, processing prawn and fish catch, and for domestic use including fencing, housing, and fuel for cooking (Rasolofo 1997). However, overexploitation has led to an increasing scarcity of forest species.

*Medicinal plants*

Ecosystems also provide numerous plant species that have huge current or potential value for medicine. One study from a single protected area indicated that 241 species are used as ethnomedicines (including 113 agricultural or weed species), providing an estimated value equivalent to 43-63% of median household income to local communities (Golden et al. 2012). The potential for developing novel biomedicines is even greater: the potential value from this watershed alone was estimated to be US$0.3-5.7 billion for American pharmaceutical companies. A separate study from forested areas in western and south-eastern Madagascar indicated 45 morphospecies, more than half of which were endemic to Madagascar, were used for medicine for gastrointestinal disorders, malaria/fever, rheumatisms, cold, skin illnesses, and inflammations (Norscia and Borgognini-Tarli 2006). Yet another study indicated that 68 plant species were used in traditional medicine in eastern Madagascar, many of which remain to be chemically tested and were in danger of being lost due to slash and burn agriculture (Novy 1997). Despite increasing access to western medicine, traditional healing using medicinal plants remains important and is complementary (Lyon and Hardesty 2005).

*Ecosystems supporting agriculture*

In Madagascar, the majority of the population is engaged in small-scale agriculture (75% of total population), and agriculture makes up 26% of GDP (World Bank 2013). The number of poor people engaged in farming is even higher:nine out of ten (Stifel et al. 2003). Natural ecosystems provide critical services that support agriculture, such as crop pollination (Bodin et al. 2006). Even the smallest forest patches were found to be essential for providing pollination services, as well as harboring lemurs that provide seed dispersal services critical for biodiversity maintenance (Bodin et al. 2006, Moses and Semple 2011). Such small patches are highly vulnerable to ongoing forest destruction, threatening the ongoing provision of ecosystem services.

*Provisioning and regulating services: Fresh water*

Freshwater services (including water quantity, water quality, and flow regulation) are among the most important ecosystem services in Madagascar, and they link to other services such as food provision and protection from floods. Below is a brief summary of types of freshwater services that were frequently referenced in the literature: fresh water for domestic use, rice irrigation and sediment regulation, and flood regulation.

*Domestic use*

Many households in Madagascar, particularly the poorest households, are reliant on unimproved sources of fresh water (i.e. rivers, streams, ponds, and lakes). A 2000 survey of 552 households in the city of Fianarantsoa (Razafindralambo et al. 2004) showed that:

28% of households rely on private taps, 33% rely on public taps, and 22% use natural sources, with a few households using wells (6%) or a private connection in some other household (8%). Not surprisingly, the higher income categories rely on private connections (54% for income category 4 and 76% of income category 5). Households in the middle income category 3 rely most on public taps (44%), although some substantial portion rely on private connections (22%) and natural sources (30%). The poorest households rely on pubic taps (36%) and natural sources (54%).

Another study of household water use indicates that, similar to many places in the world, women and girls spend the most time gathering water (Boone et al. 2011).

*Rice irrigation & sediment reduction*

Irrigation schemes supply water to about 40 percent of all cultivated lands, but many are poorly maintained so crop yields are low (World Food Programme and UNICEF 2011). This is mainly because farmers are pushing into the hills in a bid to compensate for stagnating yields in lowland areas. But upper watershed land use is often based on unsustainable management practices leading to upland soil erosion and water surface run‐off, causing sedimentation for downstream irrigation infrastructure and contributing to the flooding of crop fields in the rainy season and water shortages in the dry season (WFP and UNICEF 2011). Typically, households that grow rice as the main crop have more irrigated land (around 50% irrigated land) than households that cultivate cassava, maize, or yams as the main crop (15-20%).

Ecosystems such as Madagascar’s largest lake, Lake Alaotra, supports the country’s most fertile and productive rice fields (Bakoariniaina et al. 2006). However, past deforestation has reduced the original forest cover of Madagascar by 90%, causing siltation, which impacts agriculture and hydraulic infrastructure such as hydropower dams (Bakoariniaina et al. 2006, Rakotoarison 2003). In the past 30 years, silt from upstream deforestation has clogged the streams and rivers in the Lake Alaotra Basin, filling in most of the lake, causing it to shrink to 20% of its former size in 2000 (Bakoariniaina et al. 2006). This reduction has caused crop productivity in the basin to drop to about 40% of its former level. Existing GIS modeling and image analysis point to areas that are contributing the largest amount of silt so that remedial action can be taken.

Forests in Madagascar provide hydrological services that are important for agriculture. Not only do they produce water for the irrigation of rice fields and human consumption but they can also control the sedimentation and the erosion of watersheds (Rakotoarison 2003). Deforestation has been shown to cause siltation which decreasing productivity of irrigated agriculture, reduces the availability of drinking water, and raises costs of maintaining infrastructure such as hydroelectric dams. Collectively, these impacts result in “catastrophic economic consequences” for many economic sectors and communities (Rakotoarison 2003).

Alternatively, conservation of forests provides hydrological benefits that can be measured in monetary terms. Rakotoarison (2003) estimated economic benefits from forest conservation over 15 years to agricultural productivity $222-290 million, reduced repair costs of hydroelectric dams ($7 million), and improved drinking water quality ($6-102 million) (Rakotoarison 2003). A study of the ecosystem service values of the Ankeniheny-Zahamena Corridor (CAZ) (Portela et al. 2012) indicates that the forested corridor:

Demonstrated the potential to sustain much greater water demand than did a non-forested comparison site, which already faces critical levels of water demand. In addition, water quality, measured as reduced sediment load, was estimated to be significantly better in CAZ than in a non-conservation area. These results clearly highlight the role of forested areas such as CAZ in retaining precipitation in the form of usable water and in preventing sediment contamination of the water supply.

*Flood regulation*

One study examined flood alleviation benefits resulting from protection of upland forests in Eastern Madagsacar (Kramer et al. 1997). The authors looked at the relationship between changes in land use practices and the extent of flooding immediately downstream, as well as the impact of increased flooding on crop production in terms of lost producer surplus. They found that flooding (stormflow volume) was three times greater for a secondary forest catchment than for a same sized primary forest catchment. Catchments dominated by swidden (slash and burn) agriculture produced approximately 1.5 times more stormflow than secondary catchments. The authors concluded that land conversion from primary forests to swidden is likely to result in as much as 4.5 times more stormflow. However, forests have a decreasing ability to mitigate larger floods, and the largest (100 and 200-year) floods are less affected by land use.

*Hydropower generation*

Only 17.4% of people in Madagascar have access to electricity (World Bank 2013). For those that do, production of electrical energy is from hydropower (50.4%) and conventional thermal (49.6%) ([reegle.info](http://www.reegle.info/countries/madagascar-energy-profile/MG#sources), no date). The hydroelectric potential of the country is around 7800MW, but only about 3% are operated with a national coverage rate of approximately 21% (Rabarison 2013).

*Disaster Risk Reduction and Climate adaptation*

Five million people, a quarter of the population, live in zones at risk of natural disasters (World Bank 2013). Tropical cyclones already occur at a rate of 3-4 per year, and on average 250,000 persons are affected and US$50 million worth of damage is caused by each event (World Bank 2013). Flooding often follows cyclones; between 1990 and 2011, five major flood events were recorded, affecting more than 135,000 people; however these figures do not account for the many smaller scale events, which can cumulatively impact many more lives (World Bank 2013). The southern region of Madagascar already has very arid conditions, with less than 500mm of rainfall per year; between 1988 and 2011, 5 major drought events (each lasting two to three years) were recorded that affected at least 2.5 million persons (World Bank 2013). A single event in 2010 caused conditions that led to 720,000 people to be in a state of food insecurity.

Climate change scenarios indicate that Madagascar will be subjected to increasingly frequent and intense cyclones, floods, and droughts, which will impact biodiversity, ecosystem services and human well-being (IISD 2011, MEWF 2010, World Bank 2013). Preliminary modeling of cyclone tracks for 2100 indicates that while the total number of cyclones affecting Madagascar is unlikely to significantly increase, the frequency of intense cyclones is likely to rise. There is evidence of increasing average temperatures over recent decades, and rainfall has become less predictable, with wetter wet seasons and prolonged periods of drought (IISD 2011). Preliminary modeling indicates that in the next 40 years, the national average annual temperature will increase by up to 3°C (World Bank 2013). Key concerns related to climate change in Madagascar are related to negative impacts on agriculture and livestock, public health (especially diseases such as malaria), freshwater resources, coastal resources, and the forestry sector (IISD 2011, MEWF 2010).

There are existing efforts to identify areas within Africa that are most vulnerable to climate change at the most detailed scale possible, using existing data on physical, socio-economic, and political insecurities (Busby et al. 2010). These past efforts can be used to identify areas within Madagascar that are also most vulnerable. This information can in turn be used to identify ecosystems that are potentially reducing impacts from climate change.

Some ecosystems, such as mangroves, have demonstrated value in terms of ameliorating specific climate change impacts, such as protection from storms and shoreline stabilization (Jones 2013). As described above, mangroves provide a variety of critical services that can also help local communities adapt to climate change, including ‘provisioning’: food (e.g., fisheries and aquaculture), fuel (e.g., wood) and alternative energies (e.g., wind and wave), natural products (e.g., construction materials, sand and pearls), genetic and pharmaceutical products, ports and shipping), ‘regulating’ (e.g. carbon sequestration, shore - line stabilization, storm and flood protection, waste filtration), ‘supporting’ (e.g. soil and sediment formation, nutrient cycling) and ‘cultural’ (e.g. tourism, recreation, education) services (Jones 2013). However, mangroves are extremely threatened in Madagascar; several of the country’s largest mangrove ecosystems exhibited higher rates of loss than surrounding terrestrial forests (Jones 2013).

Coral reefs also have well-recognized value in terms of reducing impacts from climate change, both in terms of providing some protection from storm surge, and perhaps more importantly, providing critical sources of food and income that can help coastal populations cope with climate impacts (Cinner et al. 2009). However, coral reefs are themselves vulnerable to impacts form climate change. In 2005, 80% of the country’s coral reefs in the northeast of the island experienced bleaching, associated with warmer ocean waters (IISD 2011).

One study examined the resiliency of coral reefs to climate change in the Madagascar and Indian Ocean Island region (Maina et al. 2008). The authors identified the northwestern Indian Ocean and some central Indian Ocean Islands as highly susceptible, and the islands east of Madagascar as low vulnerability regions. Half of the strictly no-take zones in the region are situated in locations with medium to high susceptibility, indicating that these protections might not be sufficient if the underlying corals are vulnerable to climate impacts. The authors recommend targeting more resilient coral reefs for marine protected areas.

*Climate mitigation & deforestation*

Madagascar’s remaining forest cover also plays a key role in carbon sequestration and storage, which are important for mitigating the impacts of climate change. According to a study of the forested Ankeniheny-Zahamena Corridor (CAZ) (Portela et al. 2012):

Carbon sequestration values are very high in CAZ, suggesting that the area has high value as a continued carbon pool and sink. However, results also showed the potential for high releases of carbon if the area is managed unsustainably. Livelihoods in this region are often based on unsustainable natural resource management practices, such as *tavy* (slash and burn) agriculture and illegal logging, both of which are associated with deforestation in CAZ. This could tip the balance and quickly turn the area into a significant source of carbon emissions.

Unfortunately, 90% of Madagascar’s original forest cover is gone due to human activities (Hannah et al. 2008). Madagascar remains one of the countries with the highest rates of deforestation (ONE, DGF, FTM, MNP and CI 2013). Much of this deforestation is relatively recent: analysis of aerial photographs (c.1953) and Landsat images (c.1973, c.1990 and c.2000) indicates that forest cover decreased by almost 40% from the 1950s to c.2000 (Harper et al. 2008). This forest cover loss has real implications for climate change: globally, tropical deforestation releases 20 to 30% of anthropogenic greenhouse gases (Kremen et al. 2000).

A study of drivers of forest loss from 1990-2000 indicated that deforestation was correlated with roads and footpaths, and was not linked to population density or poverty (although the authors state that data shortcomings might explain the lack of a relationship to population or poverty) (Gorenflo et al. 2011). The authors also found that protected areas substantially slowed forest loss during the study period. The rate of deforestation within protected areas managed by Madagascar National Parks (MNP) was half the national rate (ONE, DGF, FTM, MNP and CI 2013).

Unfortunately, the effectiveness of protected areas to sustain forests may be declining. A recent study from Masoala National Park, the largest federal protected area in Madagsacar, found that the annual rate of forest change has increased, likely due to a 2009 coup d’etat and subsequent political crisis, which has resulted in increased illegal activities including logging of precious hardwoods in protected areas (Allnut et al. 2013).

Deforestation also has implications for biodiversity loss, of course. The remaining forest is highly fragmented, which means, among other threats, that there are few or no possibilities for species ranges to shift to adapt to climate change (Hannah 2008). A national-scale study of the impacts of climate change on the cost of forest conservation demonstrated that it is more cost-effective to maintain existing forest rather than invest in forest restoration, in order to conserve species (Busch et al. 2012). However, the costs and benefits of conserving forests don’t accrue to the same actors, or at the same scales. One case study from Madagascar (Kremen et al. 2000) showed that “conservation generated significant benefits over logging and agriculture locally and globally. Nationally, however, financial benefits from industrial logging were larger than conservation benefits. Such differing economic signals across scales may exacerbate tropical deforestation.”

*Cultural values & nature tourism*

Madagascar’s biodiversity and natural beauty is its largest draw for tourists, providing aesthetic and recreational values for the tourists themselves as well as livelihoods and a large portion of the country’s overall economic activity. 15% of Madagascar’s GDP is in tourism and nature tourism, providing 31,207 jobs in 2011 (Rabarison 2013). As of 2003, approximately 60,000 of Madagascar’s 200,000 annual visitors came expressly for tourism, and many others came for other reasons but included some touristic activities (Christie and Crompton 2003). National benefits of biodiversity conservation and nature tourism are valued atUS$5 per hectare of protected areas per year (Carret and Loyer 2003).

However, a study of one protected area, Ranomafana National Park, demonstrated that nature tourism created few work opportunities for local people and did not absorb job seekers who rapidly revert to survival techniques and more destructive use of resources, threatening the integrity of the forest and the long-term survival of nature tourism activities (Sarrasin 2013). Thus the author concludes that the role of nature tourism in the Malagasy economy, and the direct economic benefits of tourism at the local level, have been exaggerated.

The cultural identity of certain ethnic groups is also tied closely to their natural environment. As in many places, connections to the land (or sea) are fundamental aspects of well-being in Madagascar (Keller 2008). For example, the Vezo are fishing people of western Madagascar, and are considered “people of the sea, distinguished from the farmers around them by their economic specialism” (Astuti 2006). The Ankodida protected area in southeastern Madagascar includes a forest that is sacred to the Tandroy tribe because it is the former home of a precolonial Tandroy king (Gardner et al. 2008). The protected area is also inhabited by spirits that play an important role in the spiritual life of the tribe, as well as providing the bulk of household income for local populations.

The relationship between cultural values and conservation can be positive or negative. There is evidence that traditional protections or taboos can and have conserved certain ecosystems or species (Gardner et al. 2008, Jenkins et al. 2011). There is evidence that such traditional protections may be eroding, however (Jenkins et al. 2011). Also, the imposition of restrictions to human access and use of natural resources for conservation reasons can cause local hardship (Ferraro 2002, Golden et al. 2011) and actually undermine cultural connections to nature (Keller 2008).

**Literature Review (Table)**

B = biodiversity, W = water, CM = climate mitigation, CA = climate adaptation, F = food and non-timber forest products, C = cultural values

| **Citation** | **Ecosystem Service** | | | | | |
| --- | --- | --- | --- | --- | --- | --- |
|  | **B** | **W** | **CM** | **CA** | **F** | **C** |
| Ackermann, K. 2004. Utilisation of wild growing yams as supplementary nutrition and its impact on the dry forest ecosystem in north-western Madagascar. Swiss Forestry Journal 155: 80-88. | X |  |  |  | X |  |
| Allnut, T., G. Asner, C. D. Golden, G. Powell. 2013. Mapping recent deforestation and forest disturbance in northeastern Madagascar. Tropical Conservation Science 6: 1-15. |  |  | X |  |  |  |
| Andriamalala, G. and Gardner, C. J. 2010. L’utilisation du dina comme outil de gouvernance des ressources naturelles : leçons tirés de Velondriake, sud-ouest de Madagascar. [The use of dina as a tool for natural resource governance: lessons learned from Velondriake, southwestern Madagascar.] Tropical Conservation Science 3: 447-472. |  |  |  |  |  |  |
| Astuti, R. 2006. People of the sea: identity and descent among the Vezo of Madagascar. Cambridge University Press, Cambridge; New York. |  |  |  |  |  | X |
| Bakoariniaina, L.N., Kusky, T. and Raharimahefa, T. (2006) Disappearing Lac Alaotra: Monitoring catastrophic erosion, waterway silting, and land degradation hazards in Madagascar using Landsat imagery. Journal of African Earth Sciences 44: 241-252. |  | X |  |  |  |  |
| Barnes, D. K. A., and K. A. Rawlinson. 2009. Traditional coastal invertebrate fisheries in south-western Madagascar. Journal of the Marine Biological Association of the United Kingdom 89:1589–1596. | X |  |  |  | X |  |
| Barnes-Mauthe, M., K. L. L. Oleson, and B. Zafindrasilivonona. 2013. The total economic value of small-scale fisheries with a characterization of post-landing trends: An application in Madagascar with global relevance. Fisheries Research 147:175–185. Retrieved August 14, 2013, . |  |  |  |  | X |  |
| Barrett, C. B., P. P. Marenya, J. Mcpeak, B. Minten, F. Murithi, W. Oluoch-Kosura, F. Place, J. C. Randrianarisoa, J. Rasambainarivo, and J. Wangila. 2006. Welfare dynamics in rural Kenya and Madagascar. Journal of Development Studies 42:248–277. |  |  |  |  |  |  |
| Bernier, René and Paul A. DOROSH. 1993. Constraints on Rice Production in Madagascar: The Farmer’s Perspective. Cornell University Ilo Working Paper. February 1993. http://www.ilo.cornell.edu/images/wp34.pdf |  |  |  |  | X |  |
| Bertrand, A., B. Ramamonjisoa, and P. Montagne. 2010. Les filieres peri-urbaines d’approvisionnement en bois energie des grandes villes de Madagascar [Peri-urban supply lines of wood energy for major cities in Madagascar]. Pages 23–36 Arina, le charbon de bois a Madagascar : entre demande urbaine et gestion durable / Montagne Pierre (ed.), Razafimahatratra Serge (ed.), Rasamindisa Alain (ed.), Crehay Romain (ed.). CITE, Antananarivo. Retrieved from http://publications.cirad.fr/une_notice.php?dk=554584. | X |  |  |  |  |  |
| Bodin, Ö., M. Tengö, A. Norman, J. Lundberg, and T. Elmqvist. 2006. The Value Of Small Size: Loss Of Forest Patches And Ecological Thresholds In Southern Madagascar. Ecological Applications 16:440–451. | X |  |  |  | X |  |
| Boone, C., P. Glick, and D. E. Sahn. 2011. Household Water Supply Choice and Time Allocated to Water Collection: Evidence from Madagascar. Journal of Development Studies 47:1826–1850. Retrieved August 27, 2013, . |  | X |  |  |  |  |
| Brashares, J.S., C. Golden, K. Weinbaum, and G.V. Okello.2011. Economic and geographic drivers of wildlife consumption in rural Africa. Proceedings of the National Academy of Sciences, U.S.A. 108:13931-13936. | X |  |  |  | X |  |
| Buffle, P., M. Sassi, D. Sonetti, R. Fangareggi, and D. Cazzaniga. (n.d.). Payments for Ecosystem Services as a Means to Adapt to Climate Change in Madagascar. Retrieved from http://elanadapt.net/sites/default/files/siteimages/madagascar.pdf. |  |  |  | X |  |  |
| Burke, Lauretta, Katie Reytar, Mark Spalding and Allison Perry. 2011. Reefs at Risk Revisited. World Resources Institute. http://www.wri.org/publication/reefs-risk-revisited |  |  |  | X | X |  |
| Busby, J. W., Smith, T. G., White, K. L. and Strange, S. M. 2010. Locating climate insecurity: Where are the most vulnerable places in Africa? The Robert S. Strauss Center for International Security and Law, University of Texas, Austin. |  |  |  | X |  |  |
| Busch, J., R. Dave, L. Hannah, A. Cameron, A. Rasolohery, P. Roehrdanz, and G. Schatz. 2012. Climate Change and the Cost of Conserving Species in Madagascar. Conservation Biology. Retrieved May 1, 2012, from http://onlinelibrary.wiley.com/doi/10.1111/j.1523-1739.2012.01838.x/abstract. | X |  | X |  |  |  |
| Cardiff, S. G., Ratrimomanarivo, F. H., Rembert, G. and Goodman, S. M. 2009. Hunting, disturbance and roost persistence of bats in caves at Ankarana, northern Madagascar. African Journal of Ecology 47: 640-649. | X |  |  |  |  |  |
| Carret, J. C. and Loyer, D. 2003. Comment financer durablement le réseau d’aires protégées terrestres à Madagascar? Apport de l’analyse économique. [How to permanently finance the network of terrestrial protected areas in Madagascar? Contribution of Economic Analysis] Paper presented at Vth World Parks Congress, Durban, South Africa, 7-19 September 2003. | X | X |  |  |  | X |
| Carret, J. C., Rajaonson, B., Feno, P. J. and Brand, J. 2010. L’environnement à Madagascar: Un atout à préserver, des enjeux à maitriser. [The environment of Madagascar: an asset to preserve, challenges to master] World Bank Policy Note, Washington DC. | X |  |  |  |  |  |
| Chaperon, P. J. Danloux and L. Ferry. 1993. Fleuves et Rivieres de Madagascar. Orstom, Paris. 883 p. |  | X |  |  |  |  |
| Cheban, S. A., Rejo-Fienena, F. and Tostain, S. 2009. Etude ethnobotanique des ignames (Dioscorea spp.) dans la forêt Mikea et le couloir d’Antseva (sud-ouest de Madagascar). [Ethnobotanical study of yams (Dioscorea spp.) In Mikea forest and corridor Antseva (southwest Madagascar)] Malagasy Nature 2: 111-126. | X |  |  |  | X |  |
| Christie, I. T., and D. E. Crompton. 2003. Republic of Madagascar: Tourism Sector Study. Retrieved from http://www.worldbank.org/afr/wps/wp63.pdf. |  |  |  |  |  | X |
| Cinner, J. E., T. R. McClanahan, T. M. Daw, N. A. J. Graham, J. Maina, S. K. Wilson, and T. P. Hughes. 2009. Linking social and ecological systems to sustain coral reef fisheries. Current biology: CB 19:206–212. |  |  |  |  |  |  |
| Cinner, J., M. M. P. B. Fuentes, and H. Randriamahazo. 2009. Exploring Social Resilience in Madagascar’s Marine Protected Areas. Ecology and Society: 14. Retrieved August 14, 2013, from http://www.ecologyandsociety.org/vol14/iss1/art41/. |  |  |  |  |  |  |
| Conservation International 2011. Inventaire des Aires du Patrimoine Communautaire a Madagascar [Inventory of Community Heritage Areas in Madagascar]. Antananarivo, 2011. |  |  |  |  |  | X |
| Corson, C. 2011. From Rhetoric to Practice: How High-Profile Politics Impeded Community Consultation in Madagascar’s New Protected Areas. Society & Natural Resources:1–16. |  |  |  |  |  |  |
| Crowley, B. E., L. R. Godfrey, and M. T. Irwin. 2011. A glance to the past: subfossils, stable isotopes, seed dispersal, and lemur species loss in Southern Madagascar. American Journal of Primatology 73:25–37. | X |  |  |  |  |  |
| Damson S., Rejo-Fienena F. Tostain S. 2010. Étude ethnobotanique des ignames endémiques dans le Bas Mangoky (Sud-ouest de Madagascar) et essai de culture de quelques espèces. Dans : Les ignames malgaches, une ressource à prserver et à valoriser. [Ethnobotanical study of endemic yams in Lower Mangoky (Southwest Madagascar) and culture assay of some species. In: The Malagasy yams, a resource to preserve and enhance.] Conference proceedings of Toliara, Madagascar, 29-31 July 2009. Tostain S. Rejo-Fienena F. (eds). Pp. 60-82. | X |  |  |  | X |  |
| Dorosh, P., S. Haggblade, C. Lungren, T. Razafimanentena, B. Randriamiarana, and Zaza. 2003. Economic Motors for Poverty Reduction in Madagascar. SSRN Scholarly Paper, Social Science Research Network, Rochester, NY. |  |  |  |  |  |  |
| Dorosh, P., S. Haggblade, H. Rajemison, B. Ralantoarilolona, and K. Simler. 1998. Structure et Facteurs Déterminants de la Pauvreté à Madagascar. |  |  |  |  |  |  |
| Dostie, B., J. Randriamamonjy, and L. Rabenasolo. 1999. Cassava Production and Marketing Chains: the Forgotten Shock Absorber for the Vulnerable. Cornell University. Retrieved from http://www.cfnpp.cornell.edu/images/wp100engl.pdf. |  |  |  |  | X |  |
| Dostie, B., S. Haggblade, and J. Randriamamonjy. 2002. Seasonal poverty in Madagascar: magnitude and solutions. Food Policy 27:493–518. |  |  |  |  | X |  |
| Duclos, J.-Y., D. Sahn, and S. D. Younger. 2006. Robust Multidimensional Spatial Poverty Comparisons in Ghana, Madagascar, and Uganda. The World Bank Economic Review 20:91–113. |  |  |  |  |  |  |
| Duffy, R. 2006. Non-governmental organisations and governance states: The impact of transnational environmental management networks in Madagascar. Environmental Politics 15:731–749. |  |  |  |  |  |  |
| Fafchamps, M., and B. Minten. 2006. Crime, Transitory Poverty, and Isolation: Evidence from Madagascar. Economic Development and Cultural Change 54:579–603. |  |  |  |  |  |  |
| Fafchamps, M., and C. Moser. 2003. Crime, Isolation and Law Enforcement. Journal of African Economies 12:625–671. |  |  |  |  |  |  |
| Fenn, M., and F. Rebara. 2003. Present migration tendencies and their impacts in Madagascar’s spiny forest ecoregion. Nomadic Peoples 7:123–137. |  |  |  |  |  |  |
| Ferguson, B. 2010. Madagascar. in O. Springate-Baginski and E. Wollenberg, editors. REDD, forest governance and rural livelihoods The emerging agenda. Center for International Forestry Research. |  |  | X |  |  |  |
| Ferguson, B., and C. J. Gardner. 2010. Looking back and thinking ahead – where next for conservation in Madagascar? Madagascar Conservation & Development 5:75–76. |  |  |  |  |  |  |
| Ferraro Paul J. 2002. The local costs of establishing protected areas in low-income nations: Ranomafana National Park, Madagascar. Ecological Economics 43:261–275. |  |  |  |  |  |  |
| Freudenberger, K. 2010. Paradise Lost? Lessons from 25 years of USAID environment programs in Madagascar. International Resources Group, Washington DC. |  |  |  |  |  |  |
| Fritz-Vietta, N. V. M., H. B. Ferguson, S. Stoll-Kleemann, and J. U. Ganzhorn. 2011. Conservation in a Biodiversity Hotspot: Insights from Cultural and Community Perspectives in Madagascar. Pages 209–233 in F. E. Zachos and J. C. Habel, editors. Biodiversity Hotspots. Springer Berlin Heidelberg, Berlin, Heidelberg. Retrieved February 17, 2012, from http://www.springerlink.com/content/u13425868g112882/. |  |  |  |  |  |  |
| García, G., and S. M. Goodman. 2003. Hunting of protected animals in the Parc National d’Ankarafantsika, north-western Madagascar. Oryx 37:115–118. | X |  |  |  | X |  |
| Gardner, C. J., B. Ferguson, F. Rebara, and A. N. Ratsifandrihamanana. 2008. Integrating traditional values and management regimes into Madagascar’s expanded protected area system: the case of Ankodida. in J.-M. Mallarach, editor. Protected Landscapes and Cultural and Spiritual Values. IUCN, GTZ and Obra Social de Caixa Catalunya, Kasparek Verlag, Heidelberg. |  |  |  |  |  | X |
| Giri, C., E. Ochieng, L. L. Tieszen, Z. Zhu, A. Singh, T. Loveland, J. Masek, and N. Duke. 2011. Status and distribution of mangrove forests of the world using earth observation satellite data. Global Ecology and Biogeography 20:154–159. |  |  |  | X | X |  |
| Glick, P., and M. Razakamanantsoa. 2006. The distribution of education and health services in Madagascar over the 1990s : increasing progressivity in an era of low growth. Journal of African economies. - Oxford : Univ. Press, ISSN 0963-8024, ZDB-ID 11065680. - Vol. 15.2006, 3, p. 399-433. |  |  |  |  |  |  |
| Glick, P., S. D. Younger, and D. E. Sahn. 2006. An Assessment of Changes in Infant and under-Five Mortality in Demographic and Health Survey Data for Madagascar. SSRN Scholarly Paper, Social Science Research Network, Rochester, NY. |  |  |  |  |  |  |
| Golden, C. D. 2009. Bushmeat hunting and use in the Makira Forest, north-eastern Madagascar: a conservation and livelihoods issue. Oryx 43: 386-392. | X |  |  |  | X |  |
| Golden, C. D., L. C. H. Fernald, J. S. Brashares, B. J. R. Rasolofoniaina, and C. Kremen. 2011. Benefits of wildlife consumption to child nutrition in a biodiversity hotspot. Proceedings of the National Academy of Sciences, U.S.A. 108:19653-19656. | X |  |  |  | X |  |
| Golden, CD, BJR Rasolofoniaina, EJG Anjaranirina, L. Nicolas, L. Ravaoliny, and C. Kremen. 2012. Rainforest pharmacopeia in Madagascar provides high value for current local and prospective global uses. PLoS ONE 7(7): e41221. doi:10.1371/journal.pone.0041221 | X |  |  |  |  |  |
| Goodman, S. M. 2006. Hunting of Microchiroptera in south-western Madagascar. Oryx 40:225–228. | X |  |  |  | X |  |
| Goodman, S. M. and Raselimanana, A. 2003. Hunting of wild animals by Sakalava of the Menabe region: a field report from Kirindy-Mite. Lemur News 8: 4-6. | X |  |  |  | X |  |
| Gorenflo, L. J., C. Corson, K. M. Chomitz, G. Harper, M. Honzák, and B. Özler. 2011. Exploring the Association Between People and Deforestation in Madagascar. Pages 197–221 in R. P. Cincotta and L. J. Gorenflo, editors. Human Population. Springer Berlin Heidelberg, Berlin, Heidelberg. Retrieved December 22, 2011, from http://www.springerlink.com/content/q60n506892r48845/. | X |  | X |  |  |  |
| Haggblade, S. et al. 1999. Mécanismes amortisseurs qui jouent en faveur des ménages vulnérables. Retrieved from http://www.ilo.cornell.edu/images/wp109.pdf. |  |  |  | X |  |  |
| Hannah, L. et al. 2008. Climate change adaptation for conservation in Madagascar. Biology Letters 4:590 –594. | X |  | X |  |  |  |
| Harper, G. J., M. K. Steininger, C. J. Tucker, D. Juhn, and F. Hawkins. 2008. Fifty years of deforestation and forest fragmentation in Madagascar. Environmental Conservation 34:325–333. | X |  | X |  |  |  |
| Hockley, N. J., and R. Razafindralambo. 2006. A social cost-benefit analysis of conserving the Ranomafana-Andringitra-Pic d’Ivohibe corridor in Madagascar [Electronic resource]. Conservation International / USAID. Mission to Madagascar. |  |  |  |  |  |  |
| Horning, N. R. 2008. Strong Support for Weak Performance: Donor Competition in Madagascar. African Affairs 107:405–431. |  |  |  |  |  |  |
| International Institute for Sustainable Development. 2011. Planned Adaptation Action: Southern Africa: Botswana, Comoros, Lesotho, Madagascar, Malawi, Mauritius, Mozambique, Namibia, Seychelles, South Africa, Swaziland, Zambia and Zimbabwe. Hilary Hove, Daniella Echeverría, Jo-Ellen Parry, contributing authors. Adaptation Partnership. November 2011 http://sdwebx.worldbank.org/climateportalb/doc/USAIDProfiles/Africa_Southern_AfricaRegional_and_Country_Profiles_Final_with_new_template.pdf#page=95 |  |  |  | X |  |  |
| Irwin, M. T. et al. 2010. Patterns of species change in anthropogenically disturbed forests of Madagascar. Biological Conservation 143:2351–2362. Retrieved August 27, 2013, . | X |  |  |  |  |  |
| Jenkins, R. K. B., A. Keane, A. R. Rakotoarivelo, V. Rakotomboavonjy, F. H. Randrianandrianina, H. J. Razafimanahaka, S. R. Ralaiarimalala, and J. P. G. Jones. 2011. Analysis of Patterns of Bushmeat Consumption Reveals Extensive Exploitation of Protected Species in Eastern Madagascar. PLoS ONE 6:e27570. |  |  |  |  | X |  |
| Jenkins, R. K. B., A. Rabearivelo, C. T. C. W. M. Andre, R. Randrianavelona, and J. C. Randrianantoandro. 2009. The harvest of endemic amphibians for food in eastern Madagascar. Tropical Conservation Science 2:25–33. | X |  |  |  | X |  |
| Jones, B. 2012. Socio-economic Monitoring: A baseline assessment of the fishing villages of the Kirindy-Mite MPA. Blue Ventures Conservation Report. Retrieved August 14, 2013, from http://www.blueventures.org/conservation-reports/socio-economic-monitoring-a-baseline-assessment-of-the-fishing-villages-of-the-kirindy-mite-mpa.html. |  |  |  |  |  |  |
| Jones, J. P. G., F. B. Andriahajaina, E. H. Ranambinintsoa, N. J. Hockley, and O. Ravoahangimalala. 2006. The economic importance of freshwater crayfish harvesting in Madagascar and the potential of community-based conservation to improve management. Oryx 40:168–175. | X |  |  |  | X |  |
| Jones, T. 2013. Shining a light on Madagascar’s mangroves. Madagascar Conservation & Development 8:4–6. | X |  |  | X |  |  |
| Kari, S., and K. Korhonen-Kurki. 2013. Framing local outcomes of biodiversity conservation through ecosystem services: A case study from Ranomafana, Madagascar. Ecosystem Services 3:e32–e39. |  |  |  |  |  |  |
| Keller, E. 2008. The banana plant and the moon: Conservation and the Malagasy ethos of life in Masoala, Madagascar. American Ethnologist 35:650–664. |  |  |  |  |  | X |
| Kiefer, I., Lopez, P., Ramiarison, C., Barthlott, W. and Ibish, P. L. 2010. Development, biodiversity conservation and global change in Madagascar. In: Interdependence of Biodiversity and Development Under Global Change, P. L. Ibisch, A, Vega E. and T. M. Hermann (eds.), pp 58-81. CBD Technical Series No. 54, Secretariat of the Convention on Biological Diversity, Montreal. | X |  |  |  |  |  |
| Kramer, R. 1994. Cost and compensation issues in protecting tropical rainforests : case study of Madagascar. World Bank Environment Dept. Africa Technical Dept., [Washington D.C.]. |  |  |  |  |  |  |
| Kramer, R. A., D. D. Richter, S. Pattanayak, and N. P. Sharma. 1997. Ecological and Economic Analysis of Watershed Protection in Eastern Madagascar. Journal of Environmental Management 49:277–295. |  | X |  |  |  |  |
| Kremen, C. et al. 2008. Aligning Conservation Priorities Across Taxa in Madagascar with High-Resolution Planning Tools. Science 320:222 –226. | X |  |  |  |  |  |
| Kremen, C., J. O. Niles, M. G. Dalton, G. C. Daily, P. R. Ehrlich, J. P. Fay, D. Grewal, and R. P. Guillery. 2000. Economic Incentives for Rain Forest Conservation Across Scales. Science 288:1828 –1832. |  |  | X |  |  |  |
| Kull, C. A. 2004. Isle of fire: the political ecology of landscape burning in Madagascar. University of Chicago Press, Chicago. |  |  |  |  |  |  |
| Langley, J. M. 2006. Vezo Knowledge: Traditional Ecological Knowledge in Andavadoaka, southwest Madagascar. Blue Ventures. Retrieved from http://www.travelroots.com/downloads/bv-research-report-2006-langley-tek.pdf. |  |  |  |  |  |  |
| Le Manach, F., C. Gough, A. Harris, F. Humber, S. Harper, and D. Zeller. 2012. Unreported fishing, hungry people and political turmoil: the recipe for a food security crisis in Madagascar? Marine Policy 36:218–225. | X |  |  |  | X |  |
| Lyon, L. M., and L. H. Hardesty. 2005. Traditional Healing in the Contemporary Life of the Antanosy People of Madagascar. Retrieved August 27, 2013, from http://scholarspace.manoa.hawaii.edu/handle/10125/182. | X |  |  |  |  |  |
| Maina, J., V. Venus, T. R. McClanahan, and M. Ateweberhan. 2008. Modelling susceptibility of coral reefs to environmental stress using remote sensing data and GIS models. Ecological Modelling 212:180–199. | X |  |  | X |  |  |
| Ministry of Environment, Water and Forests [MEWF] (2010). Second National Communication to the UNFCCC. Accessed August 2012 from: http://unfccc.int/files/national_reports/non-annex_i_natcom/submitted_natcom/application/pdf/executive_summary_snc_madagascar_english.pdf |  |  |  | X |  |  |
| Minten, B. 2003. Compensation and cost of conservation payments for biodiversity. Cornell Food and Nutrition Policy Program, Cornell University, Antananarivo, Madagascar. Retrieved from http://www.ilo.cornell.edu/images/wp142.pdf. |  |  |  |  | X |  |
| Minten, B., and C. B. Barrett. 2008. Agricultural Technology, Productivity, and Poverty in Madagascar. World Development 36:797–822. |  |  |  |  | X |  |
| Minten, B., J.-C. Randrianarisoa, and C. B. Barrett. 2007. Productivity in Malagasy rice systems: wealth-differentiated constraints and priorities. Agricultural Economics 37:225–237. |  |  |  |  |  |  |
| Minten, B., L. Randrianarison, and J. F. M. Swinnen. 2005. Supermarkets, International Trade and Farmers in Developing Countries: Evidence from Madagascar. SSRN Scholarly Paper, Social Science Research Network, Rochester, NY. |  |  |  |  |  |  |
| Minten, B., R. Razafindralambo, B. Randriamiarana, Zaza, and B. A. Larson. 2002. Water Pricing, the New Water Law, and the Poor: An Estimation of Demand for Improved Water Services in Madagascar. SSRN Scholarly Paper, Social Science Research Network, Rochester, NY. |  | X |  |  |  |  |
| Mistiaen J.A., B. Özler, T. Razafimanantena, and J. Razafindravonona. 2002. "Putting Welfare on the Map in Madagascar." Africa Region Working Paper Series 34, World Bank, Washington DC, pp. 37. |  |  |  |  |  |  |
| Moser C., E. Ralison, J.F. Randrianjatovo, S. Ravelomanana. 2008. Enquête sur le suivi du recensement des communes de Madagascar Année 2007 (2007 Monitoring Census Survey of Madagascar Communes.) Final Report. February 2008. Fonds d’Intervention pour le Developpement (FID). |  |  |  |  | X |  |
| Moses, K. L., and S. Semple. 2011. Primary Seed Dispersal by the Black-and-White Ruffed Lemur (Varecia Variegata) in the Manombo Forest, South-East Madagascar. Journal of Tropical Ecology 27:529–538. | X |  |  |  |  |  |
| Muttenzer, F. 2010. Déforestation et droit coutumier à Madagascar: les perceptions des acteurs de la gestion communautaire des forêts. [Deforestation in Madagascar and customary law: perceptions of stakeholders in community forestry] Institut universitaire de hautes études internationales et du développement ; Karthala, Genève; Paris. |  |  |  |  |  |  |
| Norscia, I., and S. M. Borgognini-Tarli. 2006. Ethnobotanical reputation of plant species from two forests of Madagascar: A preliminary investigation. South African Journal of Botany 72:656–660. | X |  |  |  |  |  |
| Novy, J. W. 1997. Medicinal plants of the eastern region of Madagascar. Journal of Ethnopharmacology 55:119–126. | X |  |  |  |  |  |
| Panegos, Pauline. 2011. Le lien entre Aires Protégées et Services Environnementaux: Cas de Madagascar. pour l’obtention du Master II Economie et Gestion de l’Environnement. Université d’Antananarivo, Madagascar. pour l’obtention du Master II Economie et Gestion de l’Environnement. http://horizon.documentation.ird.fr/exl-doc/pleins_textes/divers13-04/010056054.pdf | X | X | X | X |  | X |
| Paternostro, S., J. Razafindravonona, and D. C. Stifel. 2001. Changes in Poverty in Madagascar: 1993-1999. SSRN Scholarly Paper, Social Science Research Network, Rochester, NY. |  |  |  |  |  |  |
| Peters, J. 1999. Understanding Conflicts between People and Parks at Ranomafana, Madagascar. Agriculture and Human Values 16:65–74. |  |  |  |  |  |  |
| Pollini, J. 2009. Agroforestry and the search for alternatives to slash-and-burn cultivation: From technological optimism to a political economy of deforestation. Agriculture, Ecosystems & Environment 133:48–60. |  |  |  |  |  |  |
| Pollini, J. 2010. Environmental degradation narratives in Madagascar: From colonial hegemonies to humanist revisionism. Geoforum 41:711–722. |  |  |  |  |  |  |
| Pollini, J. 2011. The Difficult Reconciliation of Conservation and Development Objectives: The Case of the Malagasy Environmental Action Plan. Human Organization 70:74–87. |  |  |  |  |  |  |
| Pollini, J., and J. P. Lassoie. 2011. Trapping Farmer Communities Within Global Environmental Regimes: The Case of the GELOSE Legislation in Madagascar. Society & Natural Resources 24:814–830. |  |  |  |  |  |  |
| Portela, R., Nunes, P.A.L.D., Onofri, L., Villa, F., Shepard, A. and G-M. Lange. 2012. Assessing and Valuing Ecosystem Services in Ankeniheny-Zahamena Corridor (CAZ), Madagascar: A Demonstration Case Study for the Wealth Accounting and the Valuation of Ecosystem Services (WAVES) Global Partnership. |  | X | X |  |  |  |
| Rabarison, Harison. 2013. Desk Study: Draft Report: Hotspot: Madagascar and Indian Ocean Islands and Outlying Islands: Hotspot Ecosystem Profile. Conservation International, Antananarivo, July 2013. | X | X | X | X | X | X |
| Rabearivony, J., E. Fanameha, J. Mampiandra, and R. Thorstrom. 2008. Taboos and social contracts: Tools for ecosystem management – lessons from the Manambolomaty Lakes RAMSAR site, western Madagascar. Madagascar Conservation & Development 3:7–16. | X |  |  |  |  |  |
| Raik, D. 2007. Forest Management in Madagascar: An Historical Overview. Madagascar Conservation & Development 2. Retrieved August 27, 2013, from http://www.ajol.info/index.php/mcd/article/view/44123. | X |  | X |  |  |  |
| Rajaspera, B., D. B. Raik, and H. Ravololonanahary. 2011. Developing a Resilient Co-Management Arrangement for Protected Areas: Field Experience From the Ankeniheny-Zahamena Corridor in Madagascar. Human Dimensions of Wildlife 16:244–258. |  |  |  |  |  |  |
| Rakotoarison, Hanitra Faratiana. 2003. Evaluation economique des benefices hydrologiques du Programme Environnement III a Madagascar. [Economic evaluation of the hydrological benefits of the Environmental Program III in Madagascar.] Universite D’antananarivo. Ecole Superieure des Sciences Agronomiques Departement Agro-Management Memoire de Fin D’etudes en vue de l’Obtention du Diplome d’Ingenieur Agronome Annee Universitaire 1998-2002. May 2003. |  | X |  |  |  |  |
| Randrianandrianina, F. H., P. A. Racey, and R. K. B. Jenkins. 2010. Hunting and consumption of mammals and birds by people in urban areas of western Madagascar. Oryx 44:411–415. | X |  |  |  | X |  |
| Randrianarisoa, C., and B. Minten. 2005. Getting the inputs right for improved agricultural productivity in Madagascar, Which inputs matter and Are the poor different? World Bank, Washington D.C. Retrieved from http://www.saga.cornell.edu/images/wp217.pdf. |  |  |  |  | X |  |
| Randrianarisoa, J. C., and B. Minten. 2001. Agricultural Production, Agricultural Land and Rural Poverty in Madagascar. SSRN Scholarly Paper, Social Science Research Network, Rochester, NY. |  |  |  |  |  |  |
| Randrianarivelojosia, M., V. T. Rasidimanana, H. Rabarison, P. K. Cheplogoi, M. Ratsimbason, D. A. Mulholland, and P. Mauclère. 2003. Plants traditionally prescribed to treat tazo (malaria) in the eastern region of Madagascar. Malaria Journal 2:25. | X |  |  |  |  |  |
| Rasoavahiny,, L., M. Andrianarisata,, A. Razafimpahanana, and A. N. Ratsifandrihamanana. 2011. Conducting an ecological gap analysis for the new Madagascar protected area system. Parks 17:12–21. | X |  |  |  |  |  |
| Rasolofo, M. V. 1997. Use of mangroves by traditional fishermen in Madagascar. Mangroves and Salt Marshes 1:243–253. | X |  |  |  | X |  |
| Ravelosoa, J. R., S. Haggblade, and H. Rajemison. 1999. Estimation des élasticités de la demande à Madagascar à partir d’un modèle AIDS. Repoblikan’i Madagasikara, Ministère des finances et de l’économie, Secrétariat général, Institut national de la statistique. |  |  |  |  |  |  |
| Razafimpahanana, A. et al. 2008. PRIORISATION : UNE APPROCHE POUR L’IDENTIFICATION DES ZONES IMPORTANTES POUR LA CONSERVATION A MADAGASCAR. [PRIORITIZATION: AN APPROACH TO IDENTIFY IMPORTANT AREAS FOR CONSERVATION IN MADAGASCAR] USAID report. Retrieved from http://www.usaid.gov/mg/so6_docs/priority_setting.pdf. | X |  |  |  |  |  |
| Razafindralambo, Ramy, Bart Minten, and Bruce Larson. 2004. Poverty and Household Water Demand in Fianarantsoa, Madagascar. http://www.csae.ox.ac.uk/conferences/2004-GPRaHDiA/papers/1p-Razafindralambo-CSAE2004.pdf |  | X |  |  |  |  |
| Réau, B. 2002. Burning for zebu: The complexity of deforestation issues in western Madagascar. Norsk Geografisk Tidsskrift - Norwegian Journal of Geography 56:219–229. | X |  |  |  | X |  |
| Robinson, G. and Pascal, B. 2009. From hatchery to community – Madagascar’s first village-based holothurian mariculture programme. SPC Beche-de-mer Information Bulletin 29: 38-43. |  |  |  |  | X |  |
| Rogers, H. M., L. Glew, M. Honzák, and M. D. Hudson. 2010. Prioritizing key biodiversity areas in Madagascar by including data on human pressure and ecosystem services. Landscape and Urban Planning 96:48–56. | X | X |  |  | X |  |
| Sarrasin, B. 2013. Ecotourism, Poverty and Resources Management in Ranomafana, Madagascar. Tourism Geographies 15:3–24. |  |  |  |  |  | X |
| Shyamsundar, P. and Kramer, R. A. 1996. Tropical forest protection: An empirical analysis of the costs borne by local people. Journal of Environmental Economics and Management 31: 129-144. |  |  |  |  |  |  |
| Stifel, D. C., and J.-C. Randrianarisoa. 2006. Agricultural policy in Madagascar: A seasonal multi-market model. Journal of Policy Modeling 28:1023–1027. |  |  |  |  |  |  |
| Stifel, D. C., B. Minten, and P. Dorosh. 2003. Transactions Costs and Agricultural Productivity: Implications of Isolation for Rural Poverty in Madagascar. SSRN Scholarly Paper, Social Science Research Network, Rochester, NY. |  |  |  |  |  |  |
| Stifel, D., F. Forster, and C. B. Barrett. 2010. The Evolution of Groupwise Poverty in Madagascar, 1999–2005. Journal of African Economies 19:559–604. |  |  |  |  |  |  |
| Stiles, D. 1998. The Mikea hunter-gatherers of southwest Madagascar: Ecology and socioeconomics. African Study Monographs 19:127–148. | X |  |  |  | X |  |
| Swartz, Wilf, Rashid Sumaila, and Reg Watson. 2012. Global Ex-vessel Fish Price Database Revisited: A New Approach for Estimating ‘Missing’ Prices. Environmental and Resource Economics. |  |  |  |  | X |  |
| The World Bank. 2013. Madagascar Country Environmental Analysis (CEA): Taking Stock and Moving Forward. Retrieved from http://www-wds.worldbank.org/external/default/WDSContentServer/WDSP/IB/2013/05/27/000333037_20130527112825/Rendered/PDF/779930WP0MDG0C00Box377320B00PUBLIC0.pdf. |  |  |  | X |  |  |
| Wendland, K. J., M. Honzák, R. Portela, B. Vitale, S. Rubinoff, and J. Randrianarisoa. 2010. Targeting and implementing payments for ecosystem services: Opportunities for bundling biodiversity conservation with carbon and water services in Madagascar. Ecological Economics 69:2093–2107. | X | X | X |  |  |  |
| World Food Program and UNICEF. 2011. Rural Madagascar Comprehensive Food and Nutrition Security and Vulnerability Analysis. http://www.wfp.org/content/madagascar-comprehensive-food-nutrition-security-vulnerability-analysis-2011 |  |  |  | X | X |  |
| **COUNT** | 46 | 13 | 12 | 13 | 34 | 9 |
