# Supplementary material for: Rapid Assessment of Ecosystem Service Co-Benefits of Biodiversity Priority Areas in Madagascar
Source: PLoS One. 2016 Dec 22;11(12):e0168575. doi: 10.1371/journal.pone.0168575 (PMC5179119; doi:10.1371/journal.pone.0168575)
Supplement: S4 Text — (DOCX) [file pone.0168575.s005.docx]

**S4 Text. Detailed methods and results**

Once the key ecosystem services had been identified through the literature review and expert consultation, we looked for existing information that could be used to assess the value of KBAs in providing these services. We collected spatial and non-spatial data on a large variety of biophysical and socioeconomic characteristics, threats, and existing land use and priorities. In general, the same data would be needed for applying the KBA+ framework in any geography. In Madagascar, low availability of up-to-date data at the national scale was overcome by using available global data.

Using existing data, past analyses, and limited new desktop analyses and modeling using GIS, we assessed the value of KBAs for the following key ecosystem services:

1. Provisioning services: Food
   1. Commercial fisheries: average landed values of fish catch
   2. Small-scale fisheries: number of food insecure people within 10 km of mangroves and coral reefs
   3. Wildlife hunting & non-timber forest products (NTFPs): number of food insecure people within 10 km of terrestrial & freshwater ecosystems (forests, mangroves, wetlands, and water bodies)
2. Provisioning services: Fresh water
   1. Relative importance for providing fresh water for domestic use
   2. Relative importance for providing fresh water for irrigation
   3. Relative importance for providing fresh water for hydropower dams
3. Regulating services: Climate mitigation
   1. Long-term carbon storage: average carbon stock per hectare and total carbon stock
   2. Potential avoided carbon emissions from deforestation
4. Regulating services: Disaster risk reduction and climate adaptation
   1. People whose vulnerability to climate change-driven increases in storm surges is reduced by mangroves
   2. People whose vulnerability to climate change-driven increases in floods is reduced by forests
5. Cultural values
   1. Nature tourism: Number of visitors to national parks in 2012 (data limited)
   2. Cultural/spiritual values (data limited)

This list does not include every ecosystem service identified as relevant in Madagascar; we limited our analysis to 1-3 key ecosystem services per category, based on expert input and data availability. Summarized methods for each service are included below.

*Provisioning: Food*

*Commercial fisheries: average landed values of fish catch*

For this analysis, we assumed that KBAs with higher levels of landed fish catch values were relatively more important for commercial fisheries. A global dataset of average landed fish value (Swartz et al. 2012) was overlaid with KBA boundaries and average landed value within each KBA was calculated.

The criteria for assessing “relative importance” of KBAs in terms of landed values of fish catch are:

- Does a given KBA provide landed fisheries values (yes/no)?
- Does a given KBA provide a landed fisheries value that is relatively larger when compared to other KBAs?

Assumptions & limitations

- This analysis includes landed fish catch values, which focuses on commercial fisheries. It assumes that there is a link between commercial fisheries and food security. This might be true, for fish that are consumed domestically and/or where incomes from commercial fishing support local food security. However, commercially caught fish might be exported and revenues might not support local income.
- There is a lack of data on subsistence level fisheries in Madagascar: at least one study indicates that total catches are actually twice the volume reported by national fisheries agencies, due to missing information about small-scale fishing (Le Manach et al. 2012).
- There is also a limitation in this analysis due to the different resolution of the data layers included: KBAs in Madagascar tend to be quite small, and the global FAO landed values dataset is coarser, thus some KBAs are smaller than a single grid cell in the FAO dataset while other KBAs intersect portions of multiple landed values cells. Therefore the average landed values per KBA should be interpreted with caution. This information might be more useful in identifying broad regions (clusters of KBAs) that have higher values, rather than comparing individual KBAs within the same region

*Small-scale fisheries: relative number of food insecure people within 10 km of mangroves and coral reefs*

For this analysis, we estimated the number of food-insecure people living within 10 km of mangrove and coral reef habitat that occurs within KBAs. We assumed that people who live closer to marine and coastal ecosystems are more likely to be benefitting from food and other resources from those ecosystems. We also assumed that people who are food insecure during part or all of the year would be particularly dependent on such resources to get them through the lean periods, as is described in the literature. Thus we used proximity to food-insecure people as an indicator of the importance of marine and coastal ecosystems.

Data on the location of mangroves and coral reefs came from two global datasets (Giri et al. 2011 and Burke et al. 2011, respectively.) Population data was based on LandScan 2011. Food insecurity rates were estimated using a commune-level census from 2007, which asked communities to self-assess food insecurity rates at the commune level (Moser et al. 2008). Focus groups in each commune were asked to estimate the percentage of people who were “poor” (defined as “Those who have problems with food safety seasonally, whether in a good or bad year”) and the percentage of people who were “destitute” (defined as “Those who do not have enough to eat throughout the year”).

The criteria for assessing “relative importance” for small-scale fisheries were:

- Does a given KBA contain marine habitats that are within 10 km of food insecure people (yes/no)?
- Does a given KBA contain marine habitats that are within 10 km of a relatively larger number of food insecure people, when compared to other KBAs?

Assumptions & limitations

- We assumed that certain ecosystem types (mangroves, coral reefs) provide food and other benefits (e.g. charcoal) to food-insecure populations
- We assumed that people living within 10 km of these ecosystems are able to access and benefit from those resources
- We assumed that people who are food insecure during part or all of the year would be particularly dependent on such resources to get them through the lean periods
- We reported results in terms of *relative* numbers of people (ranging from low to high) rather than absolute numbers, because the datasets we used are probably not precise enough to calculate absolute “counts” of people

*Wildlife hunting & non-timber forest products (NTFPs): relative number of food insecure people within 10 km of terrestrial & freshwater ecosystems*

For this analysis, we assumed that local populations probably benefit from natural terrestrial and freshwater ecosystems for wildlife hunting, collection of edible plants, medicinal plants, fuelwood/charcoal production, or other non timber forest products (NTFPs) as described by Golden et al. 2011, Brashares et al. 2011, Ackerman 2004, Damson et al. 2010, and others. We also assumed that people who are food insecure during part or all of the year would be particularly dependent on such resources to get them through the lean periods, as is also described in the literature. Thus we used proximity to food-insecure people as an indicator of the importance of terrestrial and freshwater ecosystems.

For this analysis, we relied on land cover data from Kew Royal Botanic Gardens (2007). The vegetation classes that we included are: water, mangroves, western dry forest, South western dry spiny forest-thicket, wetlands, western humid forest, humid forest, littoral forest, south western coastal bushland, western sub-humid forest, and tapia forest. Degraded forest types were excluded, as well as bare soil/rock, cultivated areas, and grasslands (we assumed most grassland in Madagascar is pasture.)

We excluded areas that were protected (data provided by CI-Madagascar). We then calculated the number of people who lived within 10 km of terrestrial and freshwater ecosystems using LandScan population data from 2008 multiplied by the estimated rate of food insecurity (Moser et al. 2008). We reported results in terms of relative numbers of people (ranging from low to high) rather than absolute numbers, because the numbers are useful as estimates but are probably not precise enough to calculate absolute “counts” of people.

The criteria for assessing “relative importance” for wildlife hunting & NTFPs are:

- Does a given KBA contain terrestrial or freshwater ecosystems within 10 km of food insecure people (yes/no)?
- Does a given KBA contain terrestrial or freshwater ecosystems that are within 10 km of a relatively large number of food insecure people, when compared to other KBAs?

Assumptions & limitations

- We assumed that terrestrial and freshwater ecosystems provide food and non-timber forest product benefits to food-insecure populations
- We assumed that people living within 10 km of these ecosystems are able to access and benefit from those resources
- We assumed that people who are food insecure during part or all of the year would be particularly dependent on such resources to get them through the lean periods
- We reported results in terms of *relative* numbers of people (ranging from low to high) rather than absolute numbers, because the datasets we used are probably not precise enough to calculate absolute “counts” of people

*Provisioning: Fresh water*

*Relative importance for providing fresh water for domestic use*

“Relative importance” of KBAs for providing fresh water for domestic (household) use was estimated using the average annual water availability in a KBA as a proportion of the overall water availability of a watershed, weighted (multiplied) by cumulative water demand downstream.

Water availability was estimated using surface water runoff calculated for current climate at 1 km^2^ resolution using version two of WaterWorld (Mulligan 2013). WaterWorld is a framework that incorporates global spatial datasets at 1 km^2^ and 1 hectare resolution, spatial models of biophysical and hydrological processes, and scenarios for climate and land use change. In its core, the FIESTA model is a process based spatially distributed model which uses variables such as vertical as well as wind driven horizontal precipitation, fog interception, infiltration rates and evapotranspiration losses to calculate water balance and surface runoff (Mulligan & Burke 2005).

The cumulative demand for fresh water for domestic use was estimated using the number of people living downstream (LandScan 2011) multiplied by average estimated annual domestic water use of 15.2 cubic meters per year per person (42.3 liters per day per person), and cumulatively summed upstream using surface water flow directions obtained from HydroSHEDS (Lehner et al. 2008). The annual per person domestic water use was based on a survey of 522 households in the city of Fianarantsoa, Madagascar (Razafindralambo et al. 2004). A buffer of 2.5 km was used to include water demand by people living alongside of major rivers (we used flow of >3 km^3^/yr to define these rivers).

Area of high “relative importance” in this analysis, is defined as areas that provide relatively more water (as a proportion of the overall water availability of a watershed) and have a relatively higher level of cumulative water demand (based on population size and per-capita water use). Thus it simultaneously highlights areas that are important for large populations depending on surface water supply from relatively smaller watersheds in which natural vegetation can plays large role in regulating water quality and quantity. This role becomes marginal in larger watersheds due to the water dilution effect.

For all freshwater analyses, continuous “wall-to-wall” maps covering the entire country were generated, then KBAs were “clipped” and average per-area values for each KBA were calculated.

Assumptions & limitations

- This analysis assumes that people are using surface water (from rivers and streams) for domestic water use. In Madagascar, this is often true, particularly for poorer households. However people also use water from wells (groundwater), piped water, or other water sources. For example, in a survey of 522 households in the city of Fianarantsoa (Razafindralambo et al. 2004):
  - “28% of households rely on private taps, 33% rely on public taps, and 22% use natural sources, with a few households using wells (6%) or a private connection in some other household (8%). Not surprisingly, the higher income categories rely on private connections (54% for income category 4 and 76% of income category 5). Households in the middle income category 3 rely most on public taps (44%), although some substantial portion rely on private connections (22%) and natural sources (30%). The poorest households rely on pubic taps (36%) and natural sources (54%).”
- This analysis assumes that every person requires 42.3 liters/day, which was the average per-person use. However, per-household use of water varied considerably with income:
  - “The lowest income-category [households] consume on average 13 liters per capita per day (for an average sized household), which is substantially below the WHO minimum recommendation of 20 liters a day per person. [Middle income-category households are] at the WHO minimum of 20 liters a day, and the higher income categories (higher for Fianarantsoa but still basically poor by international standards) are well above the 20 liters a day level standard”.
- Availability of hydrological data (water availability, water quality, water demand or other data) is limited in Madagascar. This analysis is based on existing global datasets. Ideally, it should be validated using national or sub-national hydrological data, when available.

*Relative importance for providing fresh water for irrigation*

Similarly as for water for domestic use, “relative importance” of a KBA for water for irrigation was estimated using the average annual water availability in a KBA as a proportion of the overall water availability of a watershed, weighted by estimated cumulative irrigation demand. A calculation of the cumulative surface water demand for irrigation was based on maps of areas of irrigable agriculture and estimated water demand per hectare per year, adjusted for annual rainfall. We used an average water demand of 2000 mm per year (Portela et al. 2012) corrected for half of annual rainfall. Three agricultural land classes, found in BD500 digital maps (FMT 1998), were included: 1) rice paddies, 2) “culture” (monoculture), and 3) “mosaic de culture” (mosaic of crops).

Assumptions & limitations

- There is not high quality (up to date, fine-scale) spatial data on the location of irrigated agriculture in Madagascar. We used land cover data from the BD500 dataset (FTM 1998) and assumed that the three classes: 1) rice paddies, 2) “culture” (monoculture), and 3) “mosaic de culture” (mosaic of crops) contain irrigable agriculture.
- We assumed that areas that had rice paddies, monoculture, or mosaic crops, where those areas had low precipitation, were dependent upon surface water for irrigation at least for half of the year. Most rice in Madagascar is irrigated, but some other crops (such as maize, cassava, and yams) are primarily rain-fed (World Food Programme and UNICEF 2011). However, such crops are often rotated seasonally (i.e. rice during the wet season, then other crops when the soil is drier) or grown in a mosaic within a floodplain. Irrigation in Madagascar typically comes from surface water sources (e.g. small irrigation dams and channels); other sources (such as wells or pipes) are rare.
- Actual data on water demand of different crops in Madagascar was not available for this analysis. For this analysis, demand was estimated based on a single global statistic on the per-hectare water demand of rice assuming two rice harvests per year (Portela et al. 2012). However, demand varies considerably depending on numerous factors: latitude, temperature, soils, elevation, variety of rice or other crop being grown, number of harvests per season, and other factors.
- The above described estimation is a gross assumption which may be an overestimate of the real demand by including water demand generated by the mosaic of crops category from the BD500 dataset. On the other hand, it adjusts the demand of the crops grown on the eastern side of the island where the rainfall is over 2000 mm per year.

*Relative importance for providing fresh water for hydropower dams*

Similarly as for water for domestic use, “relative importance” of KBAs in terms of providing fresh water for hydropower was estimated using the average KBA’s contribution to the overall water balance in each watershed, weighted by demand for water to generate hydropower downstream. Cumulative power in MWH generated by hydropower plants (JIRAMA 2013) was used as a proxy for actual water demand (JIRAMA 2013). This is because we were unable to obtain actual water use data.

Assumptions & limitations

- Half of Madagascar's electric power comes from hydropower generation ([reegle.info](http://www.reegle.info/countries/madagascar-energy-profile/MG#sources), no date). It is therefore safe to assume that the freshwater supply is critical for this sector. However, due to the lack of data on the actual water demand by each hydropower plant, we assumed that there is a positive linear correlation between produced electric power and water demand and used it as a proxy in the final analysis.
- In order to calculate the cumulative sum of power supplied by hydroelectric plants, we require that locations of these plants overlay directly with rivers supplying the water for their turbines. However, majority of the point locations supplied to us by JIRAMA did not align with spatial data on river locations. Therefore, we had to reassigning the locations by “snapping” them to the nearest rivers (more precisely to grid cells with greatest runoff within a 5 km radius). As a result, in some cases, locations of the hydroelectric plants may have been moved up to 5 km. However, we feel that this procedure could not have significantly affected the overall results, unless point locations were snapped to different watersheds. Better data on the location of hydroelectric dams could resolve this issue.
- We do not know how reliable the numbers for the total power (MWh) generated by hydroelectric dams are. Many of the hydroelectric dams listed in the table provided to us by JIRAMA have either missing data or values that seemed too high or too low. Following up with JIRAMA could solve this issue.

*Regulating: Climate mitigation*

*Long-term carbon storage: biomass carbon stock*

Forested areas contain biomass that provides value in terms of long-term carbon storage that can help mitigate the impacts of climate change. Some areas contain forest with comparatively high carbon density – therefore regardless of their size, these sites provide a higher number of “tons of carbon per hectare” (tC/ha).

Based on a global biomass dataset (Saatchi et al. 2011) and a 2010 forest cover dataset for Madagascar (ONE, DGF, FTM, MNP and CI 2013, including Tapia forest, mangrove, and forest classes) was used to estimate the *average* *biomass carbon stock*, measured in tC/ha, for each KBA.

The criteria for assessing “relative importance” for long-term carbon storage are:

- Does a given KBA contain biomass carbon (yes/no)?
- Does a given KBA contain relatively more biomass carbon (average or total) than other KBAs?

Assumptions & limitations

- Calculations of carbon storage are based on a global dataset. Ideally, this data would be validated using ground-based sampling of biomass carbon stock.
- Forest cover data have a resolution of 28.5m. Biomass data have a resolution of 1 km. Biomass data are estimates for the entire grid cell, not just the forested part, and thus represent the average biomass of forest and non-forest cover within that cell. Thus, the biomass data are under-estimates if interpreted as values for forest only. This is a limitation of global datasets but only becomes a significant concern at the site level in fragmented-forest landscapes.
- For this analysis, we used biomass values at 1 km and multiplied them by the areas forest cover within the 1-km cell. This results in a conservative estimate of forest biomass for partially-forested cells, which has the largest impact in the more fragmented landscapes that are mostly in the drier eastern and southern zones.

*Potential avoided carbon emissions from deforestation*

For this analysis, we calculated the deforestation rate within each KBA from 2005-2010, based on a historic deforestation analysis of Madagascar (ONE, DGF, FTM, MNP and CI 2013). The deforestation rate was multiplied by the forest area within the KBA and divided by 5 (years) to calculate the future deforestation rate in hectares per year. The hectares per year deforestation rate is multiplied by the average biomass carbon stock (tC) of the KBA, and then converted into CO_2_ equivalents (CO_2_e) in order to get an estimate of the “potential avoided carbon emissions from deforestation.” This refers to their *maximum* potential for emissions reductions (assuming deforestation is completely stopped, compared to a business-as-usual scenario based on the historical rate); feasibility studies would be needed to better estimate their actual potential for Reduced Emissions from Deforestation and Degradation (REDD+).

The criteria for assessing “relative importance” of KBAs in terms of potentially avoided carbon emissions are:

- Does a given area have a potential value in terms of avoided emissions from deforestation (yes/no)?
- Does the area have a relatively higher potential value in terms of avoided emissions, when compared to other KBAs?

Assumptions & limitations

- For now, we are using historic deforestation rate within the KBA (a percentage based on area deforested) as a proxy for potential future deforestation. However, this assumes that future deforestation will be at exactly the same rate as historic deforestation, which may or may not be true.
- This analysis is based on a global biomass carbon layer; ideally, this data would be validated with ground-based sampling of forest biomass carbon.

*Regulating: Disaster risk reduction and climate adaptation*

*Relative number of people vulnerable to climate change-driven increases in storm surges that are near mangroves*

There is mounting evidence that mangroves provide protection from storm surges generated by cyclones (Jones 2013), the frequency and/or intensity of which are projected to increase in the future in most ocean basins under climate change (IISD 2011, World Bank 2013). For this analysis, we used a global dataset that maps the number of people vulnerable to storm surges (the UNEP PREVIEW Global Risk Data Platform [http://preview.grid.unep.ch](http://preview.grid.unep.ch/)). We also used global data on the location of mangroves (Giri et al. 2011). We omitted patches smaller than 1 hectare, buffered all remaining mangrove habitats by 2 km, then identified all people classified as being at risk of storm surge that fell within 2 km of mangroves, and hence who potentially derive some degree of protection (although see assumptions listed below). We then identified all KBAs that contain mangroves that are potentially protecting vulnerable people.

The criteria for assessing “relative importance” of KBAs for storm surge protection are:

- Does a given KBA contain mangroves within 2 km of people who are vulnerable to storm surge (yes/no)?
- Does a given KBA contain mangroves within 2 km of a relatively large number of vulnerable people, when compared with other KBAs?

Assumptions & limitations

- This analysis assumes that mangroves are capable of providing some degree of protection from cyclone-driven storm surges. The actual degree of protection afforded will depend on many characteristics of both the storm event and the biophysical context – e.g. wind speed, wind direction, duration of the storm event, structural characteristics of the mangroves, bathymetry, beach topography and other factors.
- This analysis assumes that people within 2 km of mangroves receive some form of protection from those habitats - the actual distance will depend on the factors listed above, and therefore may be smaller or greater.
- The dataset of people vulnerable to storm surges is coarse when compared to the mangrove dataset, thus it is possible that the vulnerable population is further from the mangroves than estimated, or in an unprotected position (e.g. adjacent).
- For all three reasons, this analysis might over- or under-estimate the number of people protected by mangroves. Thus like above, we reported results in terms of relative numbers of people (ranging from low to high) rather than absolute counts.

*Potential flood risk reduction*

For this analysis, we were interested in where ecosystems (particularly forests) may be providing flood regulation services. Similarly as for water for domestic use, “relative importance” of a KBA for mitigation of floods was estimated using the average annual water availability in a KBA as a proportion of the overall water availability of a watershed with a forested area, weighted by estimated cumulative sum of people downstream vulnerable to flooding. For forested areas, we used the 2010 forest cover (ONE, DGF, FTM, MNP and CI 2013). Estimates of people vulnerable to flooding were adopted from the GRID population database showing people physical exposure to flooding (UNEP 2013).

Assumptions & limitations

- The main premise for the method used here was that forested areas with relatively more runoff, located upstream of people vulnerable to flooding, are more important for mitigation of floods. In other words, if this forested area is deforested, the flooding effect may worsen for the people with physical exposure to flooding.
- The regulation of floods by forests has been demonstrated at small spatial scale from at least one location in Madagascar (Kramer 1997); however, it depends on the forest characteristics (e.g., primary forests provide more regulation than secondary forests, and both provide more regulation than agricultural land cover). It also depends on the flood magnitude; forests can better regulate small- to medium-sized floods, and are less effective at large (100- to 200-year) events.
- This analysis assumes that people downstream of forests benefit from those flood regulation services. This is based simply on location and not other factors such as the ability of people to move, engineering or infrastructural solutions, or other variables.

*Cultural values*

*Nature tourism: Number of visitors to national parks in 2012 (data limited)*

For this analysis, we used data on the number of visitors to National Parks (data from Madagascar National Parks) as a proxy for overall value for nature tourism.

The criteria for assessing “relative importance” for nature tourism are:

- Did a given KBA have visitors in 2012 (yes/no/data deficient)?
- Did a given KBA have a relatively large number of visitors in 2012, when compared to other KBAs?

Limitations

- This analysis only includes data on 32 protected areas managed by Madagascar National Parks and is for only a single year (2012).
- Many other KBAs likely also have nature tourism value, and the number of visitors likely varies year to year.

*Cultural/spiritual values (data limited)*

It is known that many sites throughout Madagascar have important cultural and spiritual values, but there has been no comprehensive, national inventory. There is an inventory of the cultural values of selected protected areas (Conservation International 2011). However, data is only available for 14 out of 220 total KBAs. Similarly, there is an inventory of the cultural (religious and social) values of 51 important plant areas, based on expert opinion (Missouri Botanical Garden 2013).

However, we chose not to include these datasets, because they are not comprehensive for the entire country, and therefore make it appear that some sites have cultural values while others do not. Additional investments in research are required to better understand the value of all KBAs for providing cultural and spiritual services.

*Multi-criteria Analysis*

We were also interested in examining areas that were important for multiple services. Multiple ecosystem services from terrestrial and freshwater ecosystems were combined in a multicriteria analysis based on several of the above results: 1) biomass carbon stock, 2) number of food-insecure people with access to terrestrial/freshwater ecosystems, 3) relative importance for providing fresh water for i) domestic use, ii) irrigation, iii) hydropower, 4) relative importance for flood risk, and 5) nature tourism. We were unable to run a similar analysis for marine/coastal ecosystems, because we had too little data.

For this analysis we used IDRISI Selva software (Eastman 2012) to scale the variables and assign them individual weights. All of the input datasets were prepared so that they had the same resolution and extent. The first step in the analysis was to make sure that all of the variables were in the same scale. For our analysis we chose to execute a simple linear stretch to fit the values for each variable between the range of 0 and 255. Then each variable was put into the Multi-Criterion Evaluation (MCE) module in IDRISI and assigned a weight.

The weights given to each of the values in the multi-criteria analysis are shown in the table below. Weights were established with expert input. We decided to weight biomass carbon stock, food provision, and fresh water equally (30 out of 100) as these data are available for the entire country. For fresh water, we combined the four freshwater services (domestic use, irrigation, hydropower, and flood protection) so that they collectively added up to 30. We weighted nature tourism less (10 out of 100) because the data are available for only national parks. We tested slight variations in the weights and found similar results. The multi-criteria analysis was also repeated, excluding carbon, in order to focus on places important for “local” terrestrial & freshwater ecosystem services (food provision, nature tourism, and freshwater.)

*Weights given to each of the terrestrial/freshwater ecosystem services included in the multi-criteria analysis.*

| **Variable** | **Weight (out of 100)** |
| --- | --- |
| Total biomass carbon stock (tC) | 30 |
| Food provision (# of food insecure people within 10 km of terrestrial & freshwater ecosystems) | 30 |
| Nature tourism (# of visitors to Madagascar National Parks in 2012) | 10 |
| Relative importance for fresh water (FW) (total): | 30: |
| Relative importance of FW for domestic use | 7.5 |
| Relative importance of FW for irrigation | 7.5 |
| Relative importance of FW for hydropower | 7.5 |
| Relative importance of FW for flood protection | 7.5 |
| TOTAL | 100 |

**Results**

Below are results for several key ecosystem services (food provision, water provision, climate mitigation, disaster risk reduction/climate adaptation, and cultural values). Tabular results of the average ecosystem service values of each KBA is provided in a separate Excel file (SI5).

*1. Provisioning: Food*

*1.1 Commercial fisheries: average landed values of fish catch*

Our analysis show that 21 coastal/marine KBAs provide landed fish values (*Figure 1*). Certain KBAs in the northeast, northwest, and west of Madagascar exhibited relatively higher values, including Antogil Bay, Barren Islands, Iranja-Ankazoberavina-Russes bays, Ambodivahibe Bay, and PK32-Ranobe. These sites could be prioritized for conservation investment and carefully managed to avoid overharvest (see Appendices for a complete list of sites).


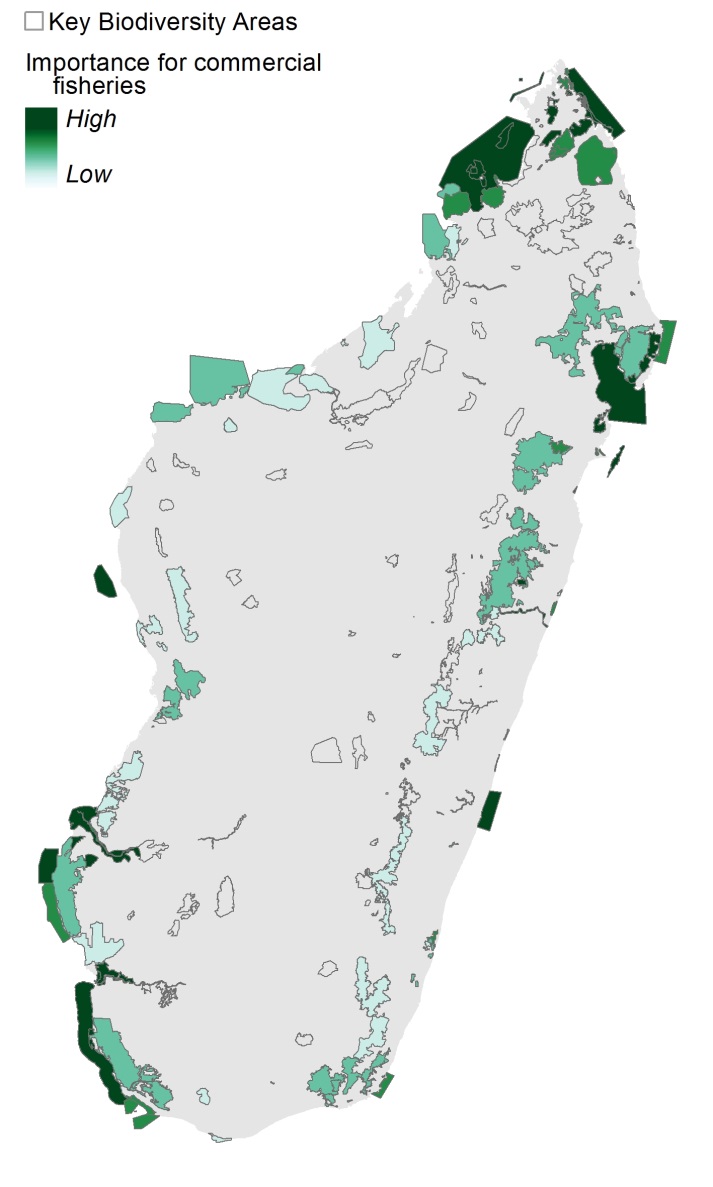


*Figure 1. Landed value of fish in KBAs, expressed as US$/ km^2^/yr (data: Swartz et al. 2012)*

*1.2 Small-scale fisheries: relative number of food-insecure people near coastal/marine ecosystems*

Many coastal/marine KBAs contain ecosystems (coral reefs and mangroves) that may serve as important sources of food to food-insecure populations (*Figure 2*). We identified a number of KBAs (42 out of 221) that contain ecosystems and are near (within 10 km) populations of food-insecure people. Examples include Sainte Marie Island (Ambohidena), Three Bays complex, Antogil Bay, Southwestern Coastal Wetlands and Nosy Manitse Future SAPM Marine, and Ampasindava/Rigny Bay (Est). These sites could be prioritized and carefully managed to avoid overharvest. (See Appendices for a complete list of sites.)

*
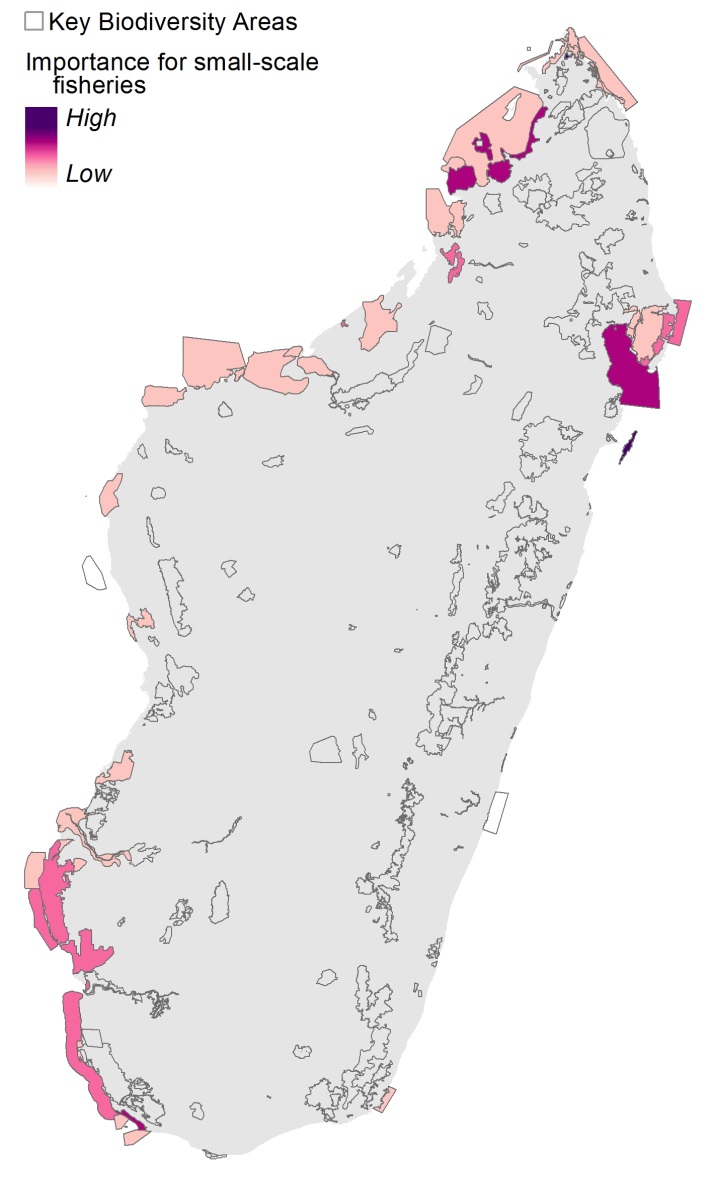
*

*Figure 2. Relative number of food insecure people living within 10 km of mangroves and coral reefs (mangrove data from Giri et al. 2011, coral reef data from WRI Reefs at Risk Revisited (Burke et al. 2011); population data from LandScan; food insecurity data from Moser et al. 2008)*

*1.3 Wildlife hunting & non-timber forest products (NTFPs): relative number of food-insecure people near terrestrial & freshwater ecosystems*

All terrestrial KBAs contain ecosystems (forests, mangroves, wetlands, and water bodies) that may serve as sources of food or non-timber forest products (NTFPs) to food-insecure populations (*Figure 3*). Most KBAs (193 out of 221) contain ecosystems that are near (within 10 km) food-insecure people. Examples include: Nankinana (Ambodibonara-Masomeloka), Manjakatompo-Ankaratra Massif, Namorona-Faraony River, Anja communuty Reserve, and Ankavia-Ankavana River (Antalaha). These sites might be prioritized if there is an interest in investing in sites that are potentially providing food and NTFPs to local communities. Such sites should be carefully managed to avoid overharvest. Mangroves were included in this analysis as well as the analysis above, as they cross the terrestrial/marine boundary.


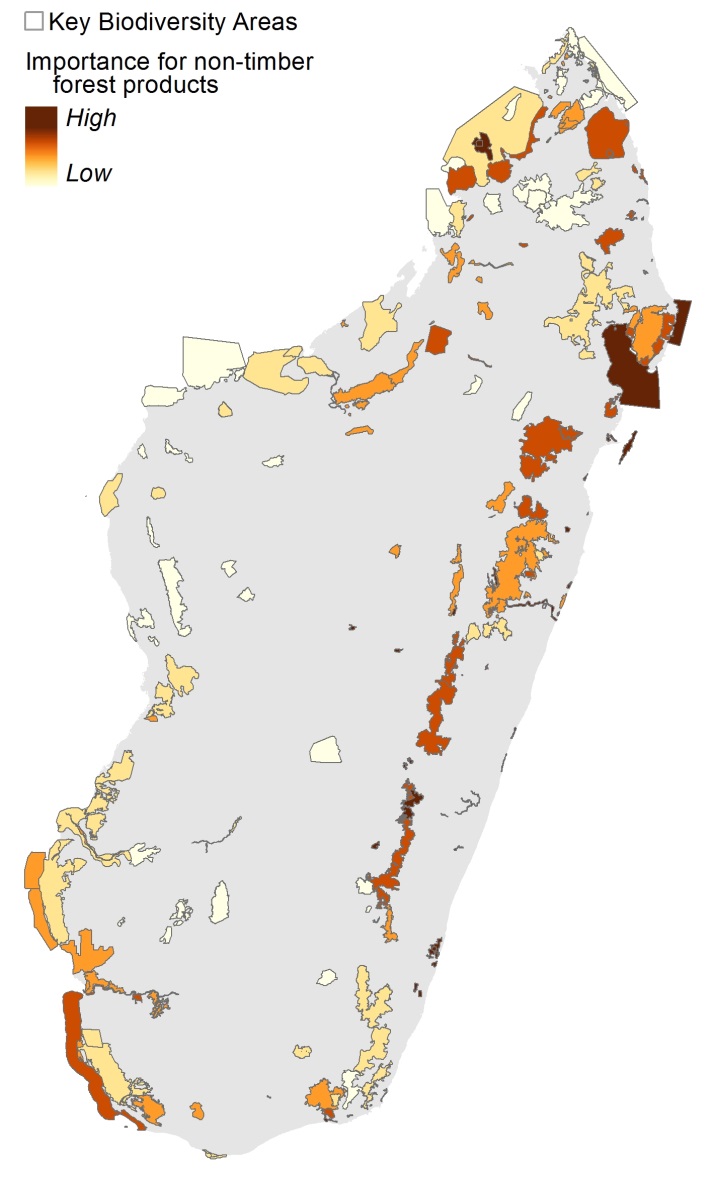


*Figure 3. Relative number of food insecure people living within 10 km of terrestrial & freshwater ecosystems (ecosystems data from Kew Royal Botanic Gardens 2007; population data from LandScan; food insecurity data from Moser et al. 2008)*

*2. Provisioning: Fresh water*

*2.1 Relative importance for providing fresh water for domestic use*

Most KBAs (203 of 221) are upstream of people and are likely to provide fresh water for drinking and other domestic uses (*Figure 4* and *Figure 5*). “Relative importance” for domestic fresh water was estimated using the average annual water availability in a KBA as a proportion of the overall water availability of a watershed, weighted by estimated water demand downstream (see Methods). KBAs in the highlands, upstream of the largest numbers of people, and KBAs in the arid northeast and southwest, where water is most scarce, appear to be relatively more important. Throughout the rest of the country, the importance of KBAs for providing water is variable.


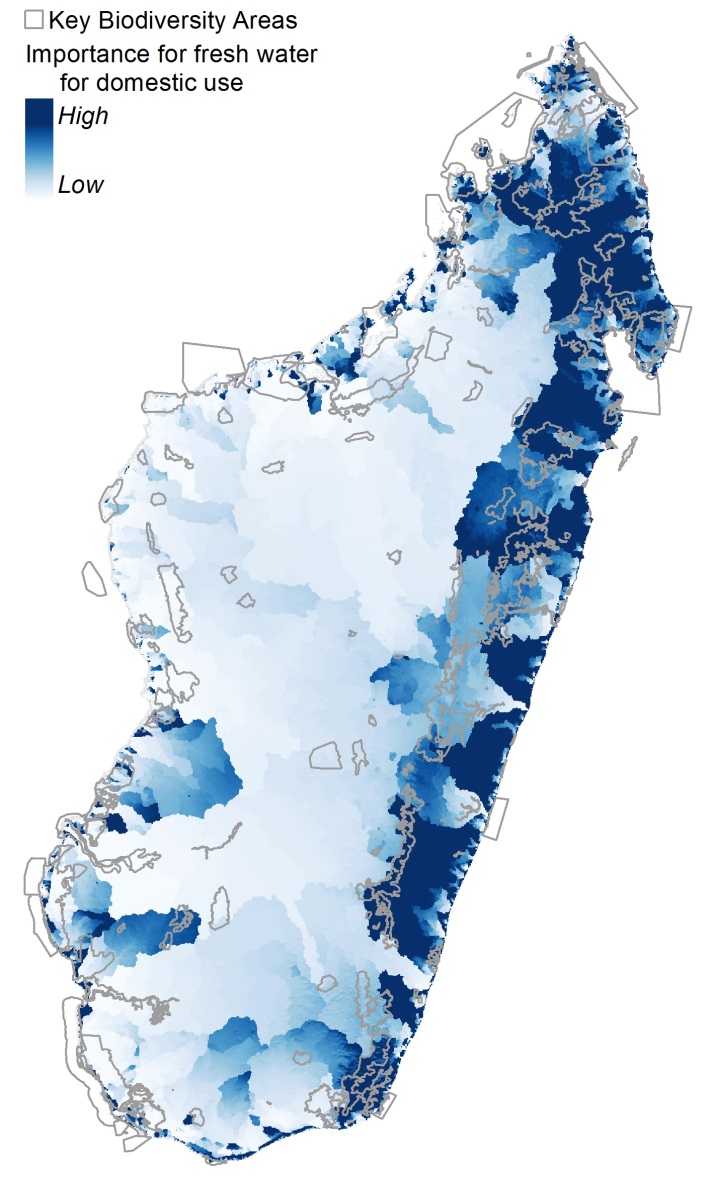


*Figure 4. Relative importance for fresh water for domestic use. (Data: WaterWorld (Mulligan 2013), LandScan)*

*
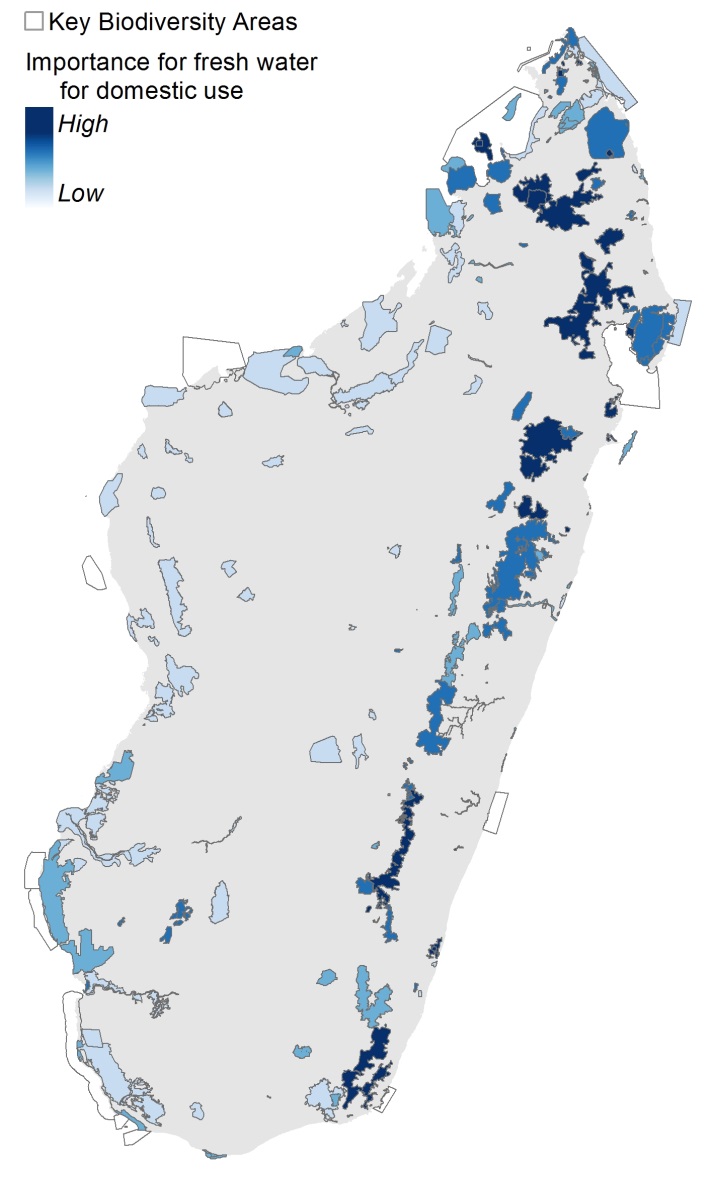
*

*Figure 5. Relative importance of KBAs for fresh water for domestic use.*

*2.2 Relative importance for providing fresh water for irrigation*

Similarly, “relative importance” of a KBA for provision of fresh water for irrigation was estimated using the average annual water availability in a KBA as a proportion of the overall water availability of a watershed, weighted by estimated irrigation demand (see Methods). Most KBAs (184 out of 221) are upstream of irrigated agricultural areas. Those with the highest relative importance are again located in the eastern highlands, where the largest number of people and highest concentration of irrigated rice agriculture occurs (*Figure 6*). But there are also relatively important areas in the east, north, and western part of Madagascar, regions characterized by larger areas of irrigated rice, as well as areas of higher aridity and lack of rain.

*
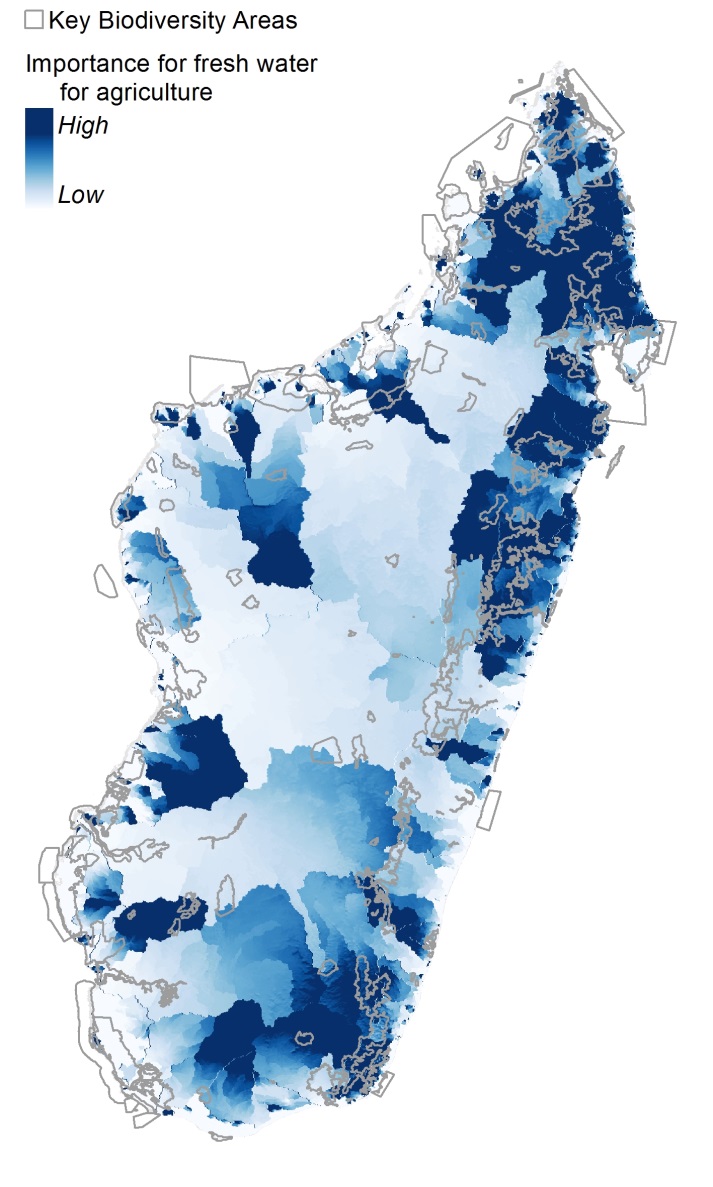

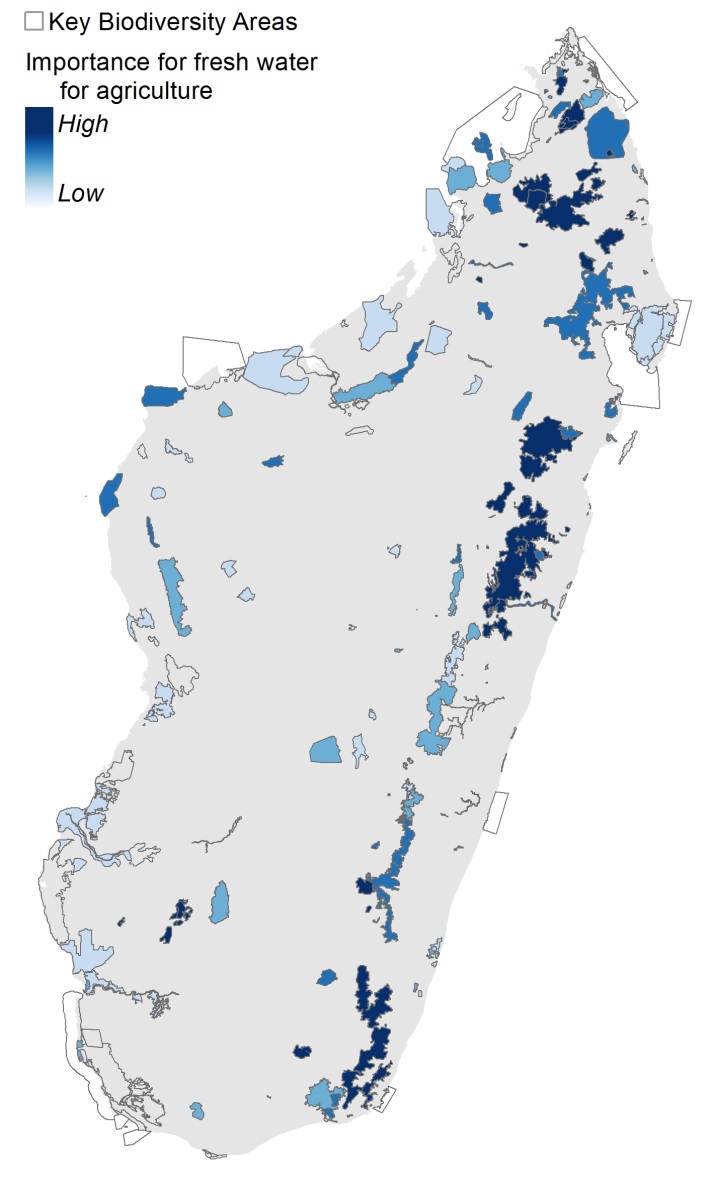
*

*Figure 6. Important areas for fresh water for irrigation (left), average importance of KBAs for fresh water for irrigation (right). (Data: Mulligan 2013 (WaterWorld), BD 500)*

*2.3 Relative importance for providing fresh water for hydropower dams*

Relative importance of KBAs in terms of providing fresh water for hydropower was estimated using the KBA’s contribution to the overall water balance in each watershed, weighted by demand for hydropower downstream (see Methods). Our analysis indicates that 38 KBAs are upstream of hydropower dams. Several KBAs in the east, north, and northwest appear to be relatively more important for hydropower (*Figure 7*). Examples include: Angavokely Forestry Station, Tsarasaotra Lake, Ankafobe, Manjakatompo-Ankaratra Massif, and Efatsy (Farafangana).


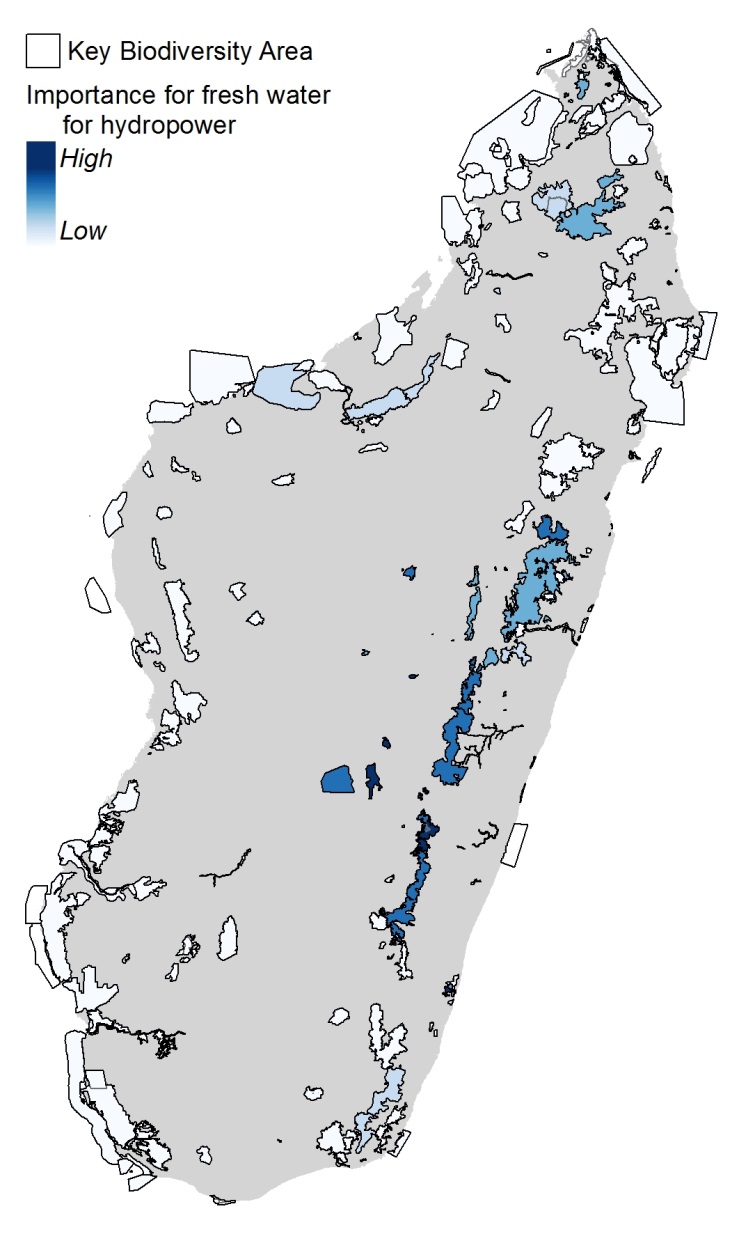


*Figure 7. Relative importance of KBAs for fresh water for hydropower dams (Data: Mulligan 2013 (WaterWorld), JIRAMA)*

*3. Regulating: Climate mitigation*

*3.1 Long-term carbon storage: average biomass carbon stock per hectare*

Virtually all of Madagascar’s remaining forest is contained within KBAs; thus these areas in relative terms contain significant value in terms of forest biomass carbon stock compared to the rest of the land (*Figure 8*). All terrestrial, forested KBAs (180 out of 221 total KBAs) contain varying amounts of biomass carbon stock.


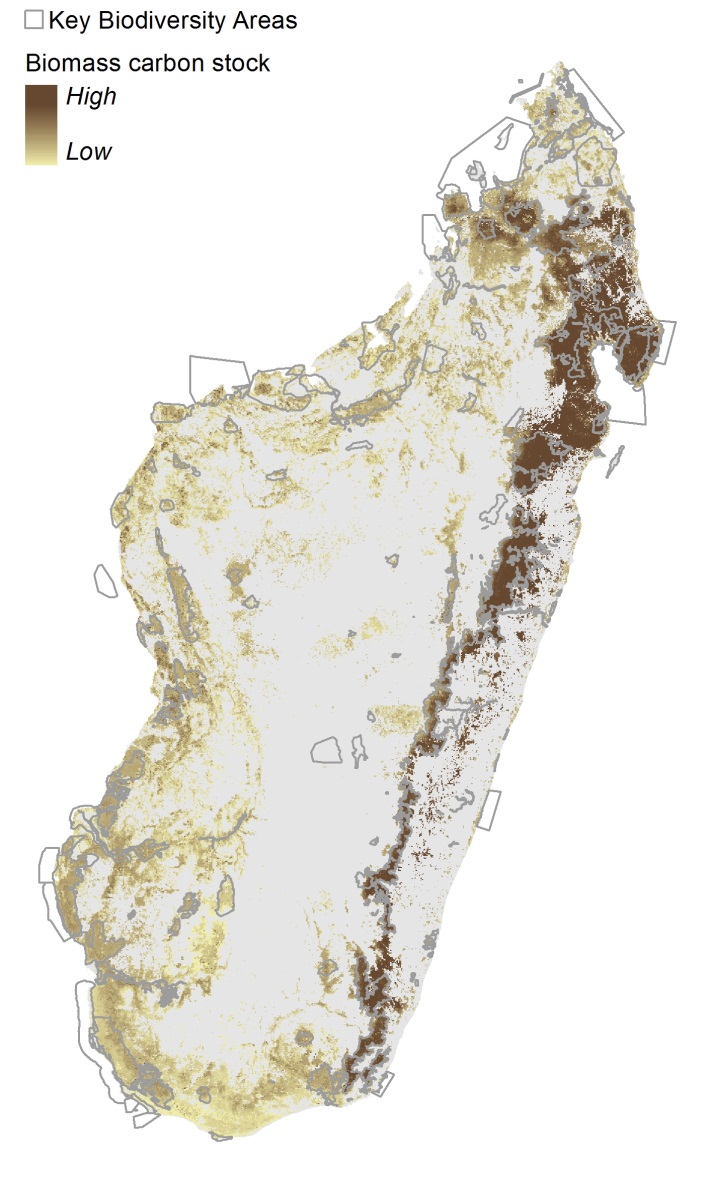


*Figure 8. Total biomass carbon in Madagascar, overlaid with Key Biodiversity Areas (KBAs). Most of the remaining forest is contained within a KBA, and therefore most of the remaining biomass carbon stock exists within KBAs. (Data source: Saatchi et al. )*

Some KBAs contain forest with comparatively high biomass carbon density as measured in tC/ha. The highest values are found in KBAs containing humid forest, particularly in the eastern highlands (*Figure 9*). Examples include Mananara-North National Park, Vohibe Ambalabe (Vatomandry), Ambatovaky Special Reserve, Analamay-Mantadia Corridor, and Masoala National Park.

*
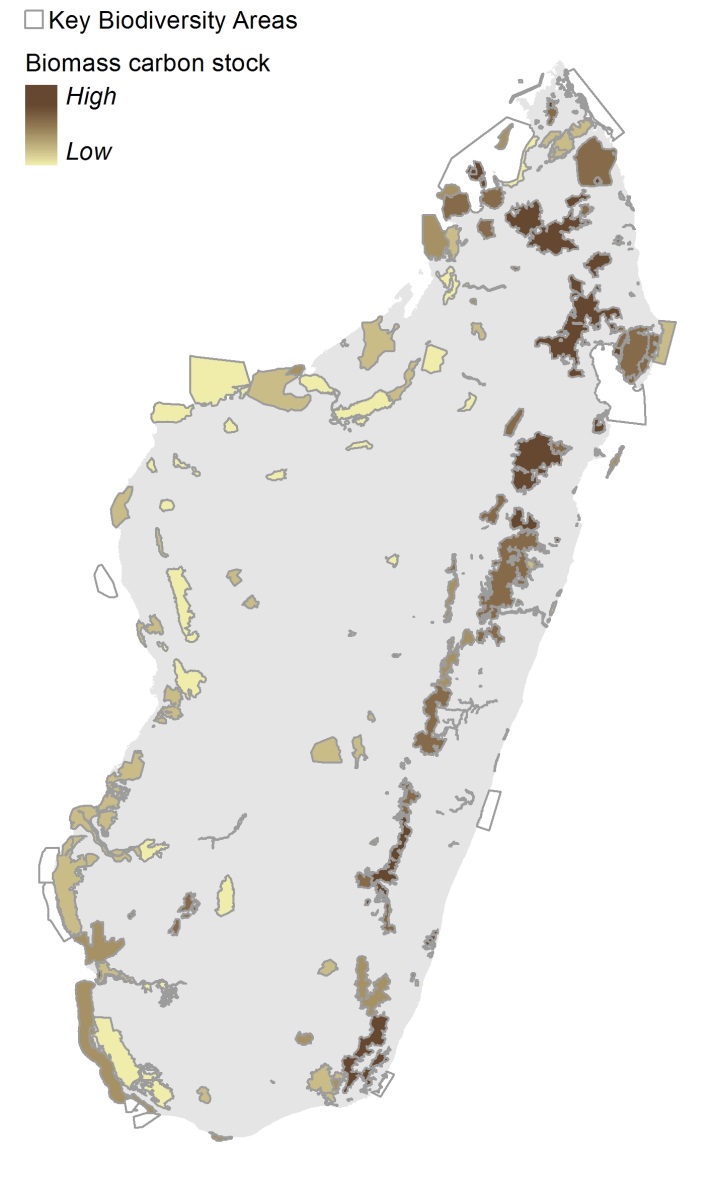
*

*Figure 9. Average biomass carbon per hectare within KBAs (tC/ha). (Data source: Saatchi et al. )*

*3.2 Potential avoided carbon emissions from deforestation*

Many KBAs (92 of 221) contain forest and have experienced historic deforestation. If conserved, these sites may have the highest estimated maximum potential for avoiding future carbon emissions from deforestation (*Figure 10*). This “estimated maximum potential” is based on the assumption that deforestation is completely stopped. Feasibility studies must be conducted if there is an interest in estimating the *actual potential* of sites for Reduced Emissions from Deforestation and Degradation (REDD+). Examples of KBAs with relatively higher estimated levels of potential avoided emissions are: PK32-Ranobe, Bidia-Bezavona Classified Forest, Ankeniheny-Lakato Future SAPM, Zahamena-Ankeniheny SAPM, and Mahafaly Plateau North Future SAPM.

*
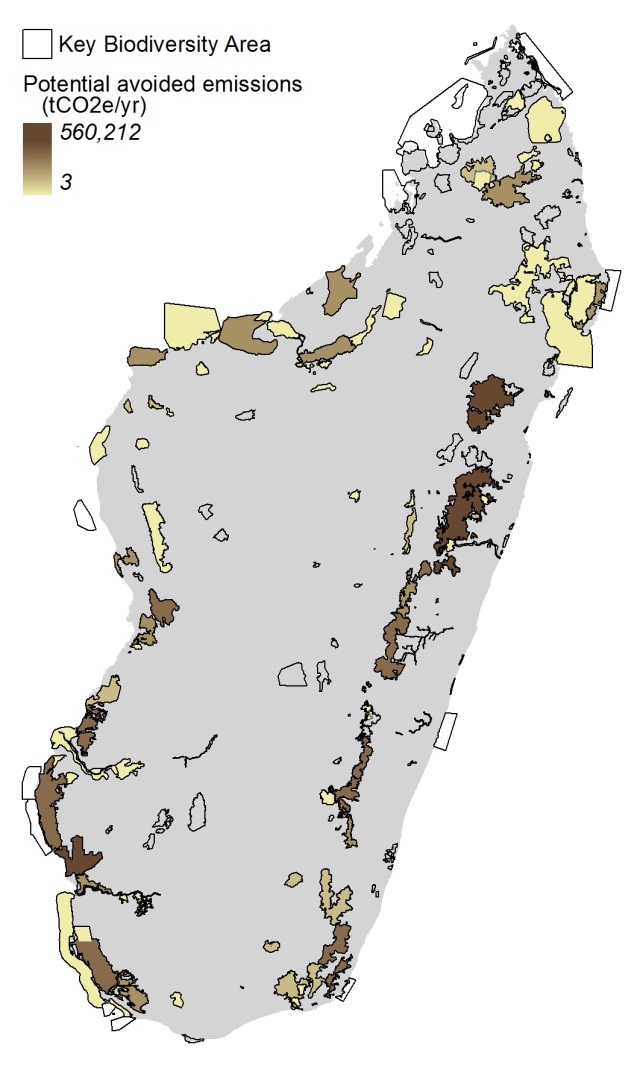
*

*Figure 10. Potential avoided emissions within KBAs, estimated based on historic deforestation rates within KBAs. (Historic deforestation data from Conservation International and biomass data from Saatchi et al. )*

*4. Regulating: Disaster risk reduction & climate adaptation*

*4.1 Number of people vulnerable to climate change-driven increases in storm surges that are near mangroves*

Twenty-eight KBAs contain mangroves within two km of people that are considered vulnerable to storm surges, based on historical cyclone events (*Figure 11*). This analysis uses historical occurrence of cyclones as a proxy for future risk, and assumes that proximity to mangroves provides some protection. Examples of KBAs that contain mangroves within two km of people who are vulnerable to cyclone surge include Amoron'i Onilahy et Onilahy River, Three Bays complex, PK32-Ranobe, Mikea Forest, and Diégo Bay. In Madagascar, cyclones primarily hit from the east and north; however remaining mangrove habitat exists primarily in the west. More research is required to understand the actual protection provided by mangroves, and the potential for mangrove restoration in the eastern part of the country.


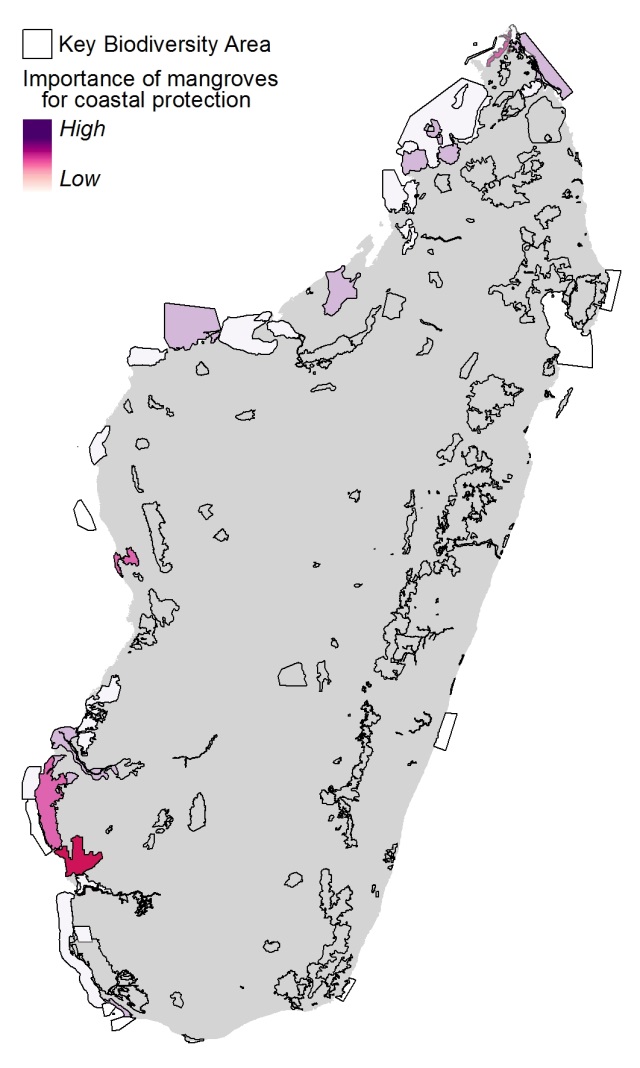


*Figure 11. Importance of mangroves for coastal protection, based on the number of people vulnerable to storm surge located within 2 km of mangroves (Data sources: Human exposure to cyclone surge data from UNEP GRID, data on mangroves from Giri et al. 2011).*

*4.2 Potential flood risk reduction*

Relative importance of KBAs for flood risk reduction was estimated based on identification of forest areas within KBAs that have a relatively high contribution to the overall water balance in each watershed, weighted by the number of people vulnerable to flooding downstream (*Figure 12*). Our analysis indicates that 123 out of 221 KBAs have potential flood reduction benefits. KBAs in the eastern and northeastern highlands showed up as relatively more important for flood risk reduction. Examples include: Angavokely Forestry Station, Anjanaharibe Sud Special Reserve, Ambohipiraka, Analalava-Analabe-Betanantanana (Ambatosoratra), and Zahamena National Park and Strict Reserve. This analysis assumes that forested areas provide some protection from flooding. There is evidence that forests provide some protection from small- and medium-sized floods; however, more research is required to better understand the role of forests in reducing floods in Madagascar.


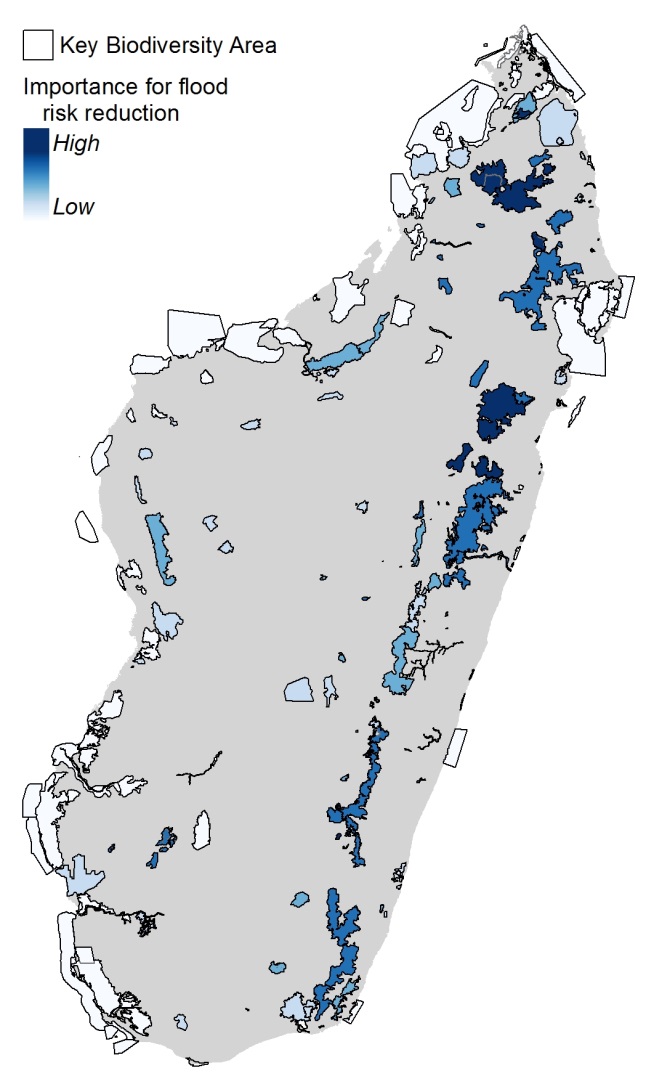


*Figure 12. Importance of KBAs for flood risk reduction, based on relative importance of an area to regulate water weighted by number of people vulnerable to flooding downstream (Data: human physical exposure to floods from UNEP PREVIEW Global Risk Data Platform, water balance data from Mulligan 2013 (WaterWorld).*

*5. Cultural values and ecotourism*

*5.1 Ecotourism: Number of visitors to national parks in 2012 (data limited)*

Data on ecotourism was only available for 32 protected KBAs managed by Madagascar National Parks. KBAs that had the largest number of visitors in 2012 include Isalo National Park, Mantadia National Park and Analamazaotra Special Reserve, Ranomafana National Park, Nosy Be and Satellites Islands (Nosy Tanihely), and Ankarana Special Reserve (*Figure 13*). Note that this data is limited to a single year. However, most ecotourism in Madagascar is centered on the national park system, thus while this dataset is incomplete, it is probably accurate to conclude that these national parks have relatively high values for ecotourism, when compared to other sites.


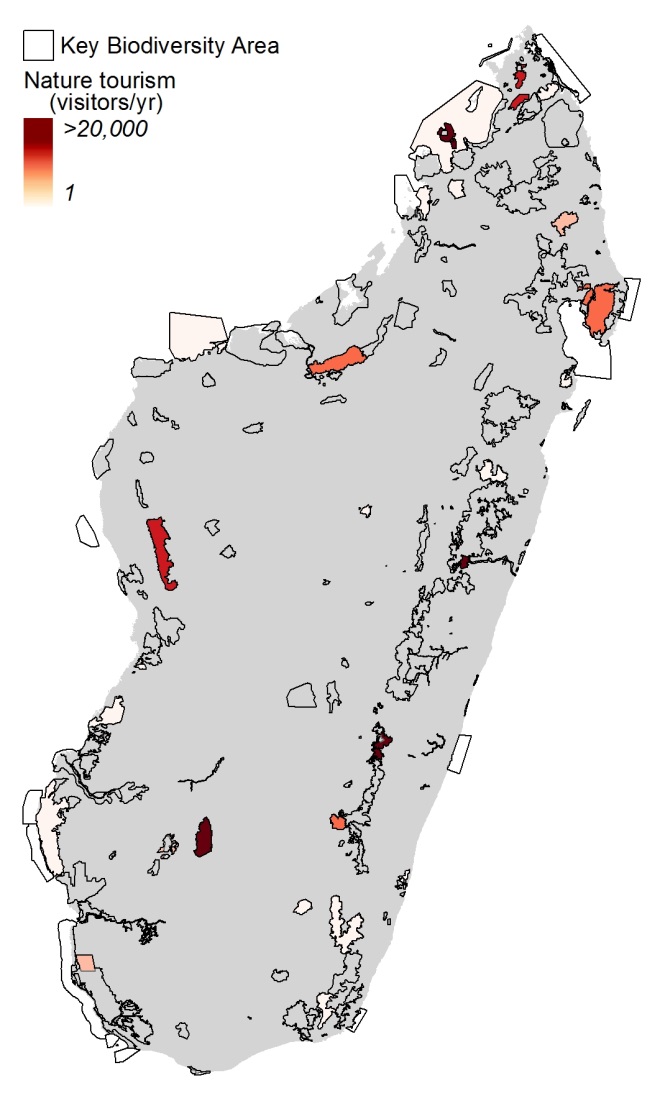


*Figure 13. Number of visitors to protected KBAs managed by Madagascar National Parks in 2012 (data: Madagascar National Parks). Note that just because a site is data deficient does not mean that there were no visitors.*

*5.2 Cultural/spiritual values (data limited)*

For this ecosystem service, data was only available for 14 out of 221 KBAs. These 14 sites were included in an inventory of community heritage areas of Madagascar (Conservation International 2011). The sites included: Ambodivahibe Bay, Andrafiamena, Bongolava Classified Forest (Marosely), Fandriana Marolambo Corridor, Ibity Future SAPM, Itremo Vakinakaratra Future SAPM, Manjakatompo-Ankaratra Massif, Montagne des Francais, Nosivolo Wetland, Vondrozo Classified Forest and surrounding areas, Zahamena National Park and Strict Reserve, and Zahamena-Ankeniheny SAPM. However; many sites throughout Madagascar have important cultural values, but were not included in this inventory. Thus a map of sites of known cultural/spiritual importance was not included because any such map would be incomplete. Additional investments in research are required to better understand the value of KBAs for providing cultural and spiritual services.

*6. Multiple Terrestrial/Freshwater Ecosystem Services*

Multiple ecosystem services from terrestrial/freshwater ecosystems were combined in a multi-criteria analysis based on several of the above results: 1) biomass carbon stock, 2) number of food-insecure people with access to terrestrial/freshwater ecosystems, 3) relative importance for providing fresh water for i) domestic use, ii) irrigation, iii) hydropower, 4) relative importance for flood risk, and 5) ecotourism (*Figure 14*). The highest value areas were found in the northeast and eastern highlands, with additional high-value areas on the southeastern side of the island. Examples include: Zahamena National Park and Strict Reserve, Mananara-North National Park, Andohahela National Park - Parcel I, Mantadia National Park and Analamazaotra Special Reserve, and Marojejy National Park. Note that this analysis includes only terrestrial and freshwater services, it does not include coastal protection, commercial fisheries, or small-scale fisheries. This map should be presented in combination with the above maps of coastal/marine services for a more complete picture. Note that areas important for providing multiple services are not necessarily “more important” than areas that are important for a single service. Thus this analysis may help to combine the above analyses, but it should not be presented in isolation.


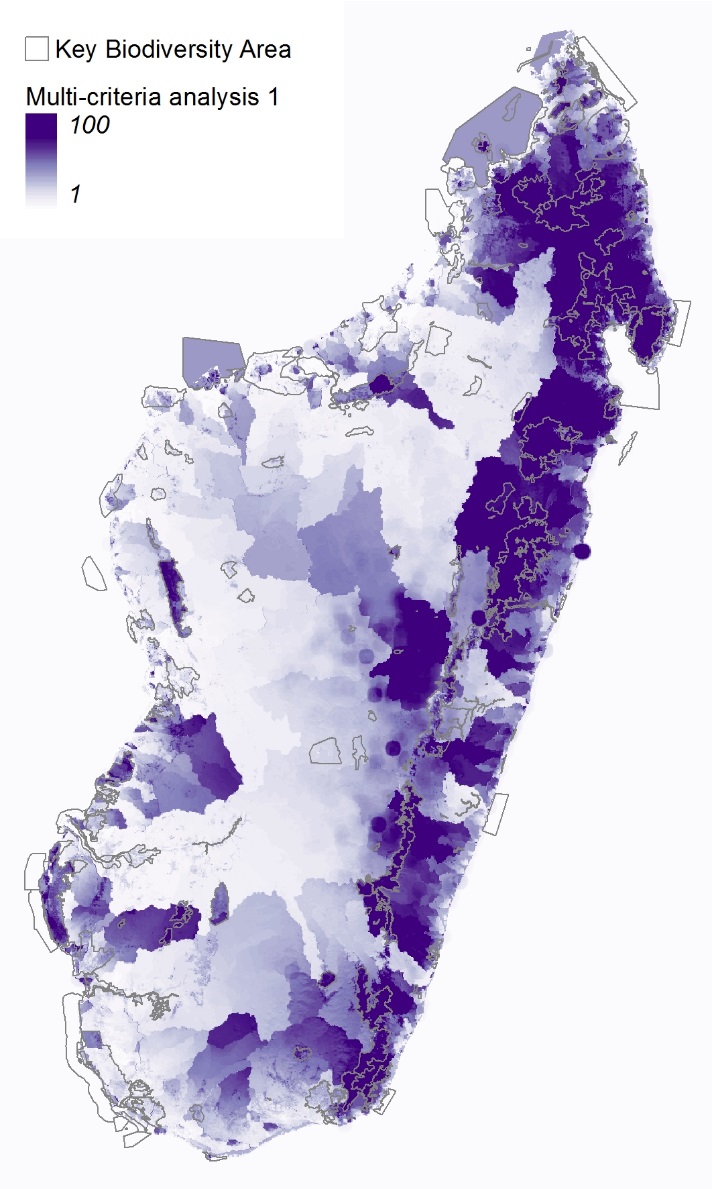


*Figure 14. Results of a multi-criteria analysis of terrestrial and freshwater ecosystem services (carbon, food, freshwater, and ecotourism).*

The second multi-criteria analysis excluded carbon in order to focus on “local” ecosystem services. Results indicate that again, areas in the eastern and northeastern Madagascar are important for multiple terrestrial & freshwater services, but also highlights some regions in the northwest and southwest (*Figure 15*). Examples of sites that are important for multiple “local” terrestrial and freshwater ecosystem services include: Zahamena National Park and Strict Reserve, Tsarasaotra Lake, Marojejy National Park, Angavokely Forestry Station, and Ankavia-Ankavana River (Antalaha). Again, this analysis excluded coastal/marine services, and this map should be presented in combination with the above maps for a more complete representation of ecosystem services in Madagascar.


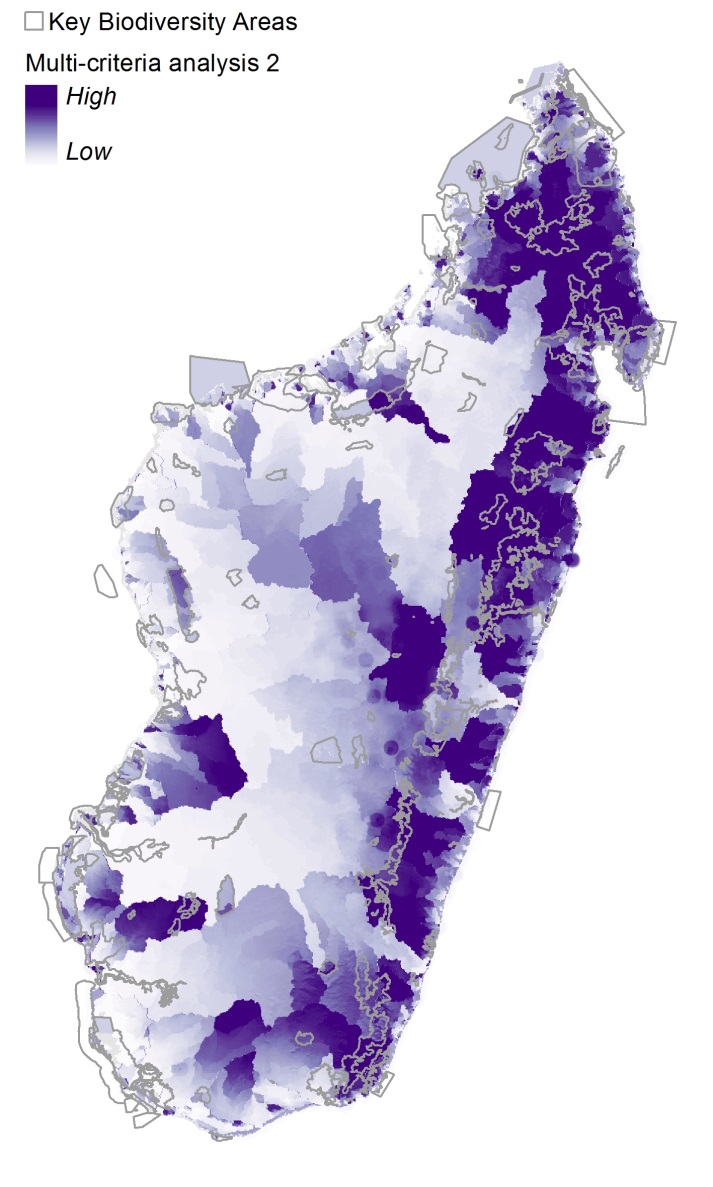

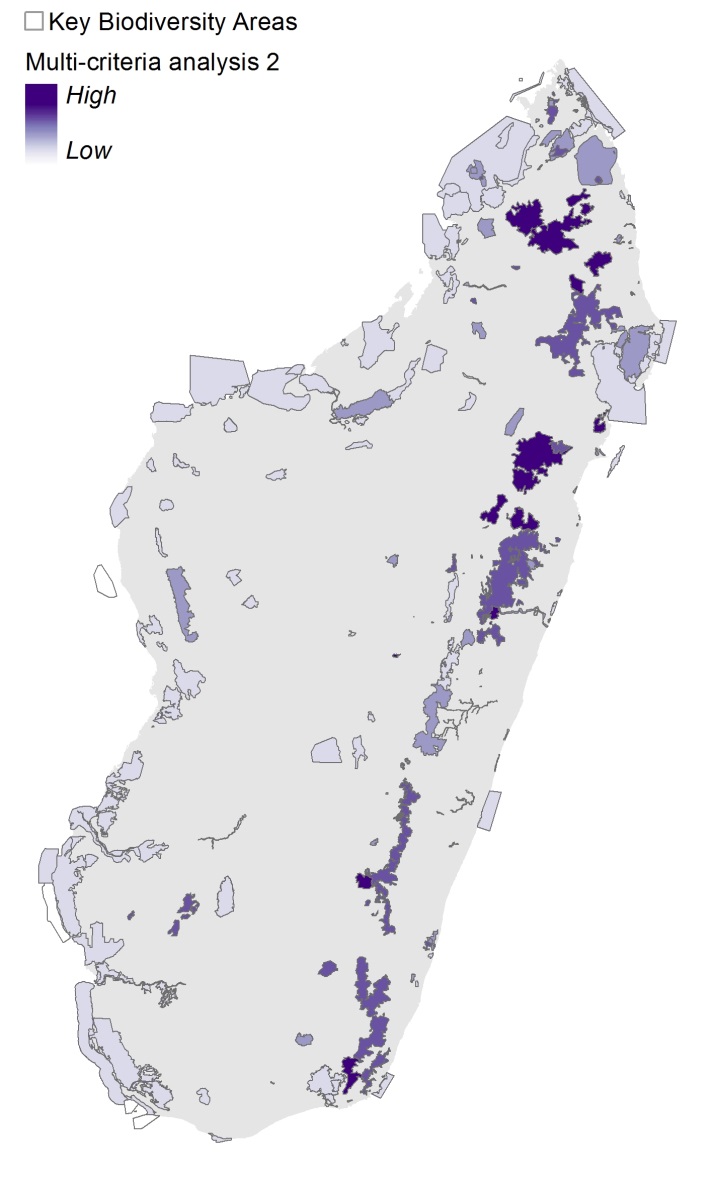


*Figure 15. Multi-criteria analysis of “local” ecosystem services: freshwater, food provision, and ecotourism*
