# Supplementary material for: Rapid Assessment of Ecosystem Service Co-Benefits of Biodiversity Priority Areas in Madagascar
Source: PLoS One. 2016 Dec 22;11(12):e0168575. doi: 10.1371/journal.pone.0168575 (PMC5179119; doi:10.1371/journal.pone.0168575)
Supplement: S5 Text — Results of different weights. (DOCX) [file pone.0168575.s006.docx]

# S5 Text. Multi-Criteria Analyses: Results of different weights

*Multi-criteria analysis: Weights 1*

| **Variable** | **Weight** |
| --- | --- |
| Biomass (tC) | 25 |
| # of food insecure people | 25 |
| Ecotourism | 10 |
| Relative importance of FW for irrigation | 10 |
| Relative importance of FW for domestic | 10 |
| Relative importance of FW for flood protection | 10 |
| Relative importance of FW for hydropower | 10 |


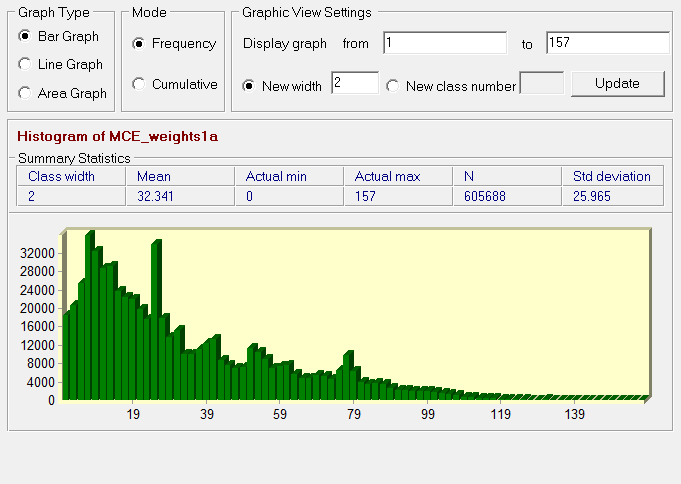

*Multi criteria analysis: Weights 2*

| **Variable** | **Weight** |
| --- | --- |
| Biomass (tC) | 30 |
| # of food insecure people | 30 |
| Ecotourism | 10 |
| Relative importance of FW for irrigation | 10 |
| Relative importance of FW for domestic | 10 |
| Relative importance of FW for flood protection | 5 |
| Relative importance of FW for hydropower | 5 |

*
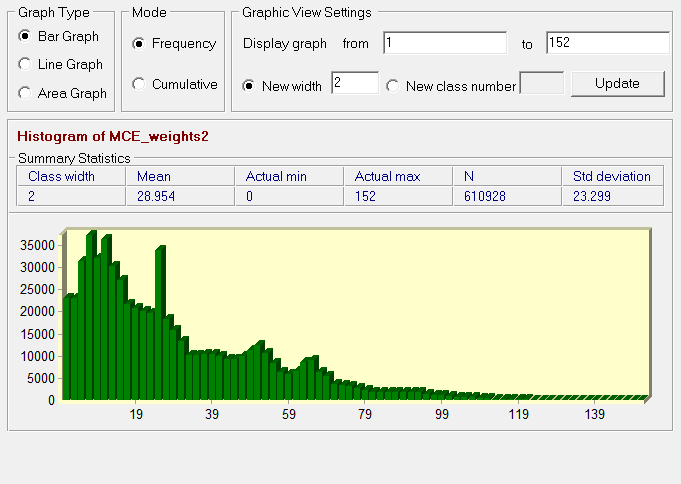
*

**

*Multi-criteria analysis: Weights 3 (SELECTED FOR ANALYSIS MCA1)*

| **Variable** | **Weight** |
| --- | --- |
| Biomass (tC) | 30 |
| # of food insecure people | 30 |
| Ecotourism | 30 |
| Relative importance of FW for irrigation | 7.5 |
| Relative importance of FW for domestic | 7.5 |
| Relative importance of FW for flood protection | 7.5 |
| Relative importance of FW for hydropower | 7.5 |

*
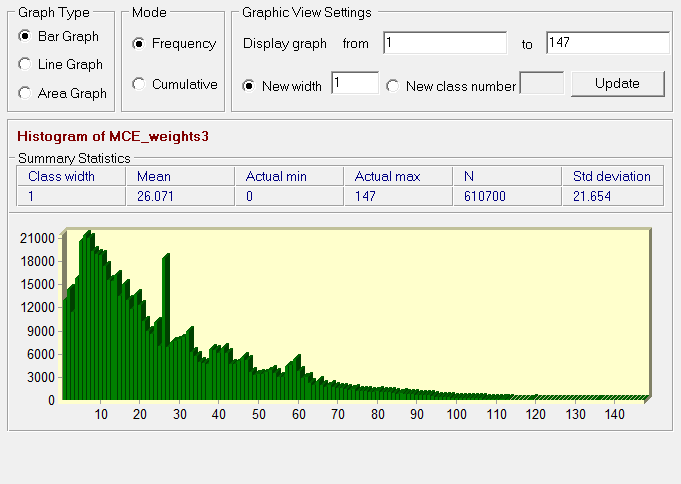
*

**

*Multi-criteria analysis: Weights 4 (carbon excluded)*

| **Variable** | **Weight** |
| --- | --- |
| Biomass (tC) | 0 |
| # of food insecure people | 30 |
| Ecotourism | 10 |
| Relative importance of FW for irrigation | 15 |
| Relative importance of FW for domestic | 15 |
| Relative importance of FW for flood protection | 15 |
| Relative importance of FW for hydropower | 15 |

*
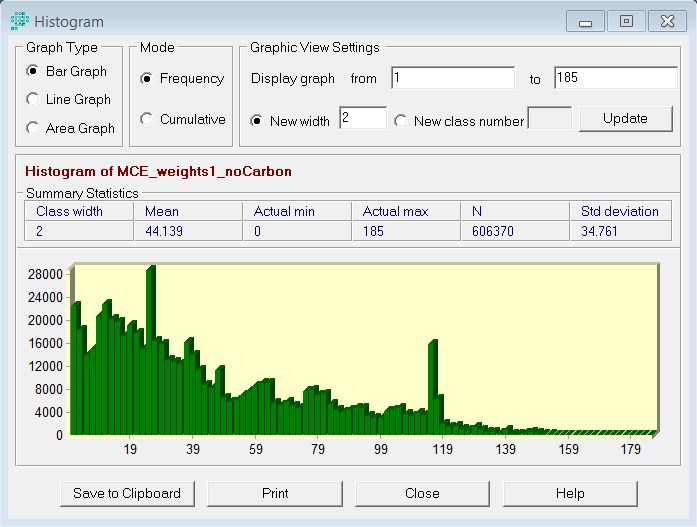
*

**

*Multi-criteria analysis: Weights 5 (carbon excluded)*

| **Variable** | **Weight** |
| --- | --- |
| Biomass (tC) | 0 |
| # of food insecure people | 45 |
| Ecotourism | 10 |
| Relative importance of FW for irrigation | 11 |
| Relative importance of FW for domestic | 11 |
| Relative importance of FW for flood protection | 11 |
| Relative importance of FW for hydropower | 11 |

*
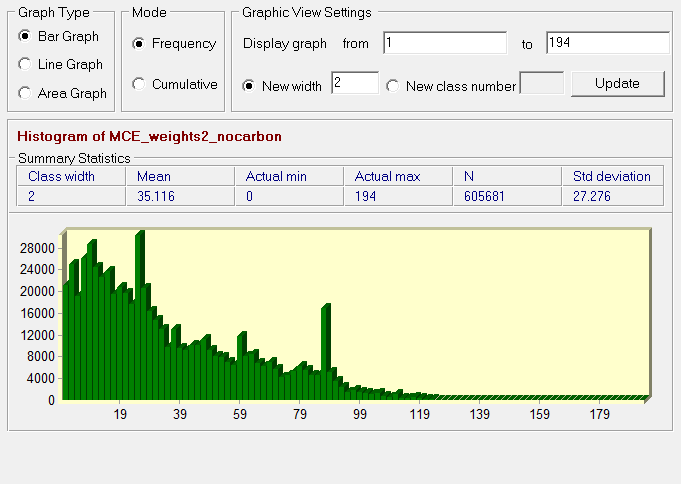
*

**

*Multi-criteria analysis: Weights 6 (carbon excluded)*

| **Variable** | **Weight** |
| --- | --- |
| Biomass (tC) | 0 |
| # of food insecure people | 30 |
| Ecotourism | 30 |
| Relative importance of FW for irrigation | 10 |
| Relative importance of FW for domestic | 10 |
| Relative importance of FW for flood protection | 10 |
| Relative importance of FW for hydropower | 10 |

*
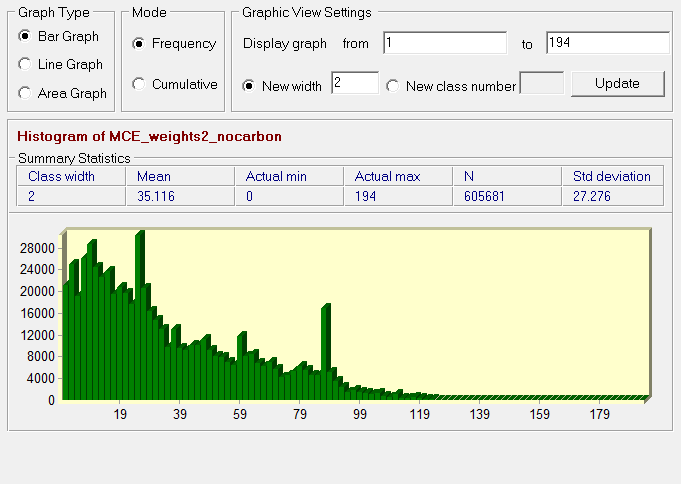
*

**
